# Emphasizing the role of oxidative stress and Sirt-1/Nrf2 and TLR-4/NF-κB in *Tamarix aphylla* mediated neuroprotective potential in rotenone-induced Parkinson’s disease: *In silico* and *in vivo* study

**DOI:** 10.1371/journal.pone.0339010

**Published:** 2026-01-06

**Authors:** Dalia H. Abu-Baih, Abeer H. Elmaidomy, Hesham A. Abou-Zied, Nahla Abdelghany Hussien, Manar I. Rasekh, Entesar A. Saber, Sayed Fouad El-sheikh Ali, Mostafa E. Rateb, Omnia Magdy Hendawy, Faisal H. Altemani, Abdullah H. Altemani, Gerhard Bringmann, Usama Ramadan Abdelmohsen, Omnia Hesham Abdelhafez

**Affiliations:** 1 Department of Biochemistry and Molecular Biology, Faculty of Pharmacy, Deraya University, New Minia City, Minia, Egypt; 2 Deraya Center for Scientific Research, Deraya University, New Minia City, Minia, Egypt; 3 Department of Pharmacognosy, Faculty of Pharmacy, Beni-Suef University, Beni-Suef, Egypt; 4 Department of Medicinal Chemistry, Faculty of Pharmacy, Deraya University, Minia, Egypt; 5 Department of Medical Science, Histology and Cell Biology, Faculty of Pharmacy, Deraya University, New Minia City, Minia, Egypt; 6 Department of Anatomy, Faculty of Medicine, Minia University, Minia, Egypt; 7 Natural and Medical Sciences Research Center, University of Nizwa, Nizwa, Oman; 8 Department of Pharmacology, College of Pharmacy, Jouf University, Skaka, Saudi Arabia; 9 Department of Clinical Pharmacology, College of Medicine, Beni-Suef University, Beni-Suef, Egypt; 10 Department of Medical Laboratory Technology, Faculty of Applied Medical Sciences, University of Tabuk, Tabuk, Saudi Arabia; 11 Department of Family and Community Medicine, Faculty of Medicine, University of Tabuk, Tabuk, Saudi Arabia; 12 Institute of Organic Chemistry, University of Würzburg, Würzburg, Germany; 13 Department of Pharmacognosy, Faculty of Pharmacy, Deraya University, New Minia, Egypt; University College London, UNITED KINGDOM OF GREAT BRITAIN AND NORTHERN IRELAND

## Abstract

Parkinson’s disease (PD) presents as a progressive deterioration of dopaminergic neurons, a process closely associated with increased oxidative damage due to accumulated reactive oxygen species, leading to weakened antioxidant defenses and ultimately neuronal dysfunction. Currently, no definitive approach exists to counteract the degeneration of dopaminergic neurons in PD. The use of *Tamarix aphylla* as a protective agent against Parkinson’s disease is not well studied yet. In this study, a rotenone-induced rodent model was utilized to examine the neuroprotective potential of *T. aphylla* extract. The chemical composition of *T. aphylla* leaves was analyzed through LC-HR-ESI-MS profiling, identifying 13 metabolites from various chemical categories. Furthermore, the research incorporated the STRING database and Cytoscape software to perform a protein-protein interaction (PPI) analysis, pinpointing essential hub proteins involved in neuroprotection and inflammation in PD. Molecular docking and a 150 ns molecular dynamics simulation were performed to assess the interaction of plant-derived compounds with the Sirt-1 catalytic domain. Compound **12**, one of the bioactive compounds found in *T. aphylla*, exhibited strong binding affinity and stability throughout the 150 ns simulation, highlighting its role as a neuroprotective agent. This study underscores the fusion of computational and experimental techniques to investigate natural neuroprotective compounds, providing potential therapeutic strategies for PD treatment by influencing key pathways linked to oxidative damage and neuroinflammation.

## Introduction

Parkinson’s disease (PD) is recognized as the second most prevalent neurodegenerative disorder, marked by the gradual loss of dopaminergic neurons located in the substantia nigra pars compacta (SNpc) and the formation of Lewy bodies, which primarily contain abnormal alpha-synuclein (α-Syn) aggregates [1,2]. The origin of PD is veiled in a complex network of interconnected pathological mechanisms, involving mitochondrial dysfunction, oxidative stress, and neuroinflammatory pathways, with α-Syn accumulation playing a key role in the onset of neurodegeneration. Under normal conditions, α-Syn maintains a delicate equilibrium between its free monomeric form and structured tetramers, preventing aggregation [3]. While some symptoms can be alleviated through existing treatments, the ongoing search for effective interventions to slow down or halt disease progression remains unresolved [3]. However, in disorders associated with synucleinopathies such as PD, native α-Syn transforms into harmful structures—oligomers, protofibrils, or fibrils—which contributes to a detrimental shift in neural integrity [3]. Under physiological milieu, α-Syn navigates a delicate equilibrium between its unstructured monomeric form and structured tetramers, maintaining a precarious balance that deters aggregation [4]. However, in disorders associated with synucleinopathies such as PD, native α-Syn transforms into harmful structures—oligomers, protofibrils, or fibrils—which contributes to a detrimental shift in neural integrity [5,6]. Currently, no definitive approach exists to prevent the degeneration of dopaminergic neurons in PD. Conventional treatments, including dopamine agonists and L-3,4-dihydroxyphenylalanine (L-DOPA), function merely as symptom-relieving therapies rather than providing a long-term disease-modifying solution [7].

The activation of the Sirt-1/Nrf2 axis has emerged as a promising therapeutic pathway, demonstrating neuroprotective potential by enhancing mitochondrial efficiency, reducing oxidative damage, strengthening antioxidant defenses, and promoting cellular survival [8]. Disruptions in the precise regulation of the Sirt-1/Nrf2 signaling cascade have been linked to the progression of neurodegenerative diseases, including PD. Ongoing studies exploring the intricate relationship between oxidative stress, the Sirt-1/Nrf2 pathway, and PD are shedding light on viable therapeutic targets that offer hope for managing and alleviating this debilitating condition [9,10].

Considering the crucial impact of oxidative stress and neuroinflammation in triggering the degeneration of dopaminergic neurons, research is now shifting toward natural compounds recognized for their health benefits, therapeutic potential, and pharmacological significance. This growing interest is fueled by compelling evidence supporting the efficacy of various natural substances in addressing oxidative stress and neuroinflammation, thereby contributing to positive outcomes in the field of neurodegenerative diseases, including PD [11,12]. In efforts to combat oxidative stress and inflammation-induced dopaminergic neurodegeneration, the application of natural bioactive compounds with strong antioxidant and anti-inflammatory properties presents a promising strategy to protect dopaminergic neurons and slow the relentless advancement of disease progression [13].

Among the diverse pharmacological agents utilized to replicate the pathological characteristics of PD—including reserpine, haloperidol, MPTP, paraquat, and rotenone—the latter stands out due to its classification as an environmental toxin capable of mimicking the neurodegenerative and behavioral manifestations associated with this disorder [14,15]. Taking advantage of its fat-soluble nature, rotenone effectively crosses the blood-brain barrier, exerting harmful effects by selectively inhibiting complex I of the mitochondrial electron transport chain.

This disruption ultimately resulted in the accumulation of reactive oxygen species (ROS), a reduction in glutathione levels, and the consequent loss of dopaminergic neurons. Additionally, exposure to rotenone initiates a series of molecular and cellular changes characterized by heightened oxidative stress and neuroinflammation, facilitated by microglia and astrocytes activation, along with the downregulation of proteasomal function. These pathological processes collectively contribute to the accumulation of α-Syn-containing nigral aggregates, a distinctive neuropathological marker of PD [16,17].

Natural products have revealed their biological importance in treatment of various diseases especially neurological disorders [18–20]. In this context, *Tamarix aphylla* (Tamaricaceae) is a medium-sized tree found in different regions in Africa, Middle East, and Western Asia [21–23]. It has been used for different purposes in folk medicine, as an anti-spasmodic, wound healing, and abscess-treatment agent [24]. The plant is rich with different metabolites such as tannins, flavonoids, and phenolics [25]. Different investigations have revealed its biological importance as an antioxidant, anti-inflammatory, antimicrobial, and wound healing remedy [22].

In this research, the impact of *T. aphylla* in mitigating rotenone-induced Parkinsonian symptoms has been investigated. Furthermore, it has been distinctly demonstrated that the extract safeguards dopaminergic neurons from oxidative stress-triggered inflammation, α-Syn aggregation, and cell death. The function of the Sirt-1/Nrf2 signaling pathway in providing neuroprotection against nigrostriatal dopaminergic neuron degeneration was also examined.

## Materials and methods

### Plant material

The plant *T. aphylla* was collected from the desert of Minia Governorate. The leaves were identified by Dr. Abd ElHalim A. Mohammed (Department of Flora and Phytotaxonomy Research, Dokki, Cairo, Egypt). A voucher specimen (2022-BuPD 91) has been deposited at the Department of Pharmacognosy, Faculty of Pharmacy, Beni-Suef University, Egypt.

### Plant extraction

The leaves (2.0 kg) were dried in the shade and macerated in methanol (3 × 7 L, 7 d each) at room temperature. The liquid methanolic extract was concentrated under reduced pressure at 45˚C utilizing a rotary evaporator (Buchi Rotavapor R-300, Cole-Parmer, Vernon Hills, IL, USA) giving 300 g total extract.

### LC-HR-ESI-MS

The crude methanolic extract from *T. aphylla* leaves was prepared at a concentration of 1 mg/mL for mass spectrometry analysis. The methanolic extract was analyzed by a metabolic study using LC-HR-ESI-MS in accordance with Shamikh et al., 2014 [26]. The data obtained from the analyzed methanolic extract were dereplicated using the DNP database [27].

### Computational studies

#### Network of interacting proteins.

The main goal of this study was to investigate how dereplicated compounds extracted from *Tamarix aphylla* contribute to neuroprotection in the context of Parkinson’s disease. To achieve this, the research employed the STRING database to uncover critical molecular interactions that may play a role in mediating these protective effects. It was assumed that if the confidence value was greater than 0.4, the interaction existed. Cytoscape and the CytoHubba plug-in were used for network mapping and analysis to reveal critical hub genes that bear implication in the processes of neuroprotection and inflammation. Subsequently, the research highlighted crucial proteins and biological targets that could help counteract the degeneration of dopamine-producing nerve cells associated with Parkinson’s disease, offering promising new avenues for therapeutic development.

#### Prediction of therapeutic targets for *Tamarix aphylla.*

This study leveraged the SwissTargetPrediction platform (http://www.swisstargetprediction.ch/) to forecast likely protein targets of bioactive compounds obtained from *T. aphylla*. Structural analysis and comparison with established interaction profiles led to the prediction of 100 candidate protein targets for the investigated compounds. This approach had a dual impact, enhancing the specificity of target prediction while also proving beneficial for assessing therapeutic strategies for PD. The integration of three-dimensional structural data not only deepened molecular-level insights but also contributed to the bioengineering of prototypes for drug repurposing, facilitating the discovery of novel neuroprotective targets through the platform.

#### Gene ontology (GO) and Kyoto Encyclopedia of Genes and Genomes (KEGG) based functional enrichment analysis.

The neuroprotective role of *Tamarix aphylla* in a Parkinsonian model was explored through functional enrichment, involving GO-based classification and KEGG-mediated pathway mapping of the predicted molecular targets. The results were grouped into three functional domains: biological roles, cellular localization, and molecular-level activities. ShinyGO was used for analysis with a stringent FDR threshold (< 05), and results were visualized via SRplot. The results provided valuable insights into the functional roles of genes within inflammation- and apoptosis-related pathways linked to Parkinson’s disease, thereby supporting the identification of novel therapeutic candidates.

#### Docking studies.

Molecular docking studies were utilized to simulate the interaction between proteins involved in PD-associated pathways and potential neuroprotective metabolites identified from *T. aphylla*. Molecular docking was conducted using AutoDock 4.2 (https://autodock.scripps.edu/), and the resulting ligand–target complexes were visualized and analyzed using the Discovery Studio Visualizer (BIOVIA, Dassault Systèmes), a free academic tool available for non-commercial research and educational use. The tool can be freely accessed at the Dassault Systèmes BIOVIA website: https://discover.3ds.com/discovery-studio-visualizer-download. The study identified key metabolites with the potential to interact effectively with proteins involved in Parkinson’s disease pathology, notably Sirt-1. Resveratrol, a recognized neuroprotective agent and Sirt-1 modulator, was employed as a reference ligand in the docking simulations [28]. Protein models were sourced from the RCSB Protein Data Bank (http://www.rcsb.org/) and subjected to structural refinement, including hydrogen atom addition and charge adjustment, to ensure accurate interaction analysis. This docking analysis reinforced network pharmacology findings by verifying potential neuroprotective interactions.

#### *In silico* molecular dynamics.

GROMACS 2023 was employed to carry out molecular dynamics simulations, aiming to confirm docking predictions and analyze the conformational stability of protein–ligand interactions over time [29]. Protein structures, such as Sirt-1, were processed and refined using UCSF Chimera to ensure optimal geometry for downstream molecular simulations [30], To optimize the system, hydrogen atoms were incorporated into the protein structure. The CHARMM36 force field was used for proteins, whereas CGenFF was applied to the ligand molecules. A solvated simulation box was built using the TIP3P water model, maintaining a minimum spacing of 1 nm from the solute in every direction [31]. Sodium and chloride ions were introduced to balance the charge of the system, achieving a final concentration of 150 mM. The simulation initiated with energy minimization through the steepest descent algorithm, followed by NVT and NPT ensemble equilibration at 300 K and 1.0 bar. A 150-ns production phase was conducted without constraints to capture trajectory data for further evaluations, including RMSD and binding energy analysis. This detailed MD simulation provided substantial evidence supporting the stability and therapeutic potential of *T. aphylla* compounds in alleviating PD pathology.

#### Animals.

From the animal facility at Deraya Center for Scientific Research, 48 healthy Sprague-Dawley male rats (280–320 g) were procured. The study received ethical approval from the ethics committee of Deraya Center for Scientific Research (Approval No: DCSR-01024–04), and all procedures involving the animals complied with the ARRIVE guidelines [32]. Care was taken to ensure minimal discomfort for the animals. The rats were housed under regulated conditions, maintaining a 12-h light/dark cycle at a temperature range of 22–25°C and a relative humidity of 50 ± 5%, with unrestricted availability of food and water.

#### Experimental grouping and animal administration.

After a 1-week acclimatization period, rats were allocated into four groups (12 rats/ group): Group 1: a control group, group 2: a *T. aphylla* group, group 3: a rotenone group, and group 4: a *T. aphylla* + rotenone group. The control group and the rotenone-treated group were given 0.5% CMC through intragastric (ig) administration once per day. The groups of *T. aphylla* and *T. aphylla* + rotenone rats were given a dose of 125 mg/kg of *T. aphylla* daily through oral administration [33]. One h after the administration of 0.5% CMC or *T. aphylla*, the rats in the rotenone group and in the *T. aphylla* + rotenone group received subcutaneous injections of 2 mg kg − 1·d − 1 rotenone dissolved in sunflower oil once per day for 28 d [34]. Additionally, the control group received an injection of the same volume of the solvent. Following a 24-h period of administering the last rotenone injection, rats were anesthetized using 80 mg/kg ketamine and then euthanized by decapitation. Sections of the brain were isolated and stored for further examination.

#### Hematoxylin and eosin routine staining.

Paraffin-embedded beeswax tissue blocks were sectioned to a thickness of 4 μm using a sliding microtome. The resulting specimens were carefully mounted onto glass slides, after which the paraffin was removed. A staining procedure utilizing hematoxylin and eosin (H&E) was then applied. An unbiased researcher subsequently examined the samples using a light microscope equipped with a camera [35].

#### Immunohistochemistry.

For immunohistochemistry, paraffin sections were prepared at a thickness of 5 μm, adhering to the protocol established by Johansson et al. [36]. Immunohistochemical staining was conducted following the manufacturer’s instructions, utilizing a TH rabbit monoclonal antibody at a dilution of 1:200 (Abcam, Cambridge, UK; catalog number ab75875).

#### Oxidative stress assessment.

To quantify oxidative stress in brain tissues, we measured the levels of key antioxidant biomarkers, including catalase (CAT), reduced glutathione (GSH), and superoxide dismutase (SOD), alongside the oxidative marker malondialdehyde (MDA). These analyses were conducted by available assay kits (Biodiagnostics, Giza, Egypt) [37,38].

#### Quantitative real-time polymerase chain reaction.

RNA was isolated from brain tissues using TRIzol reagent following standard protocols. The isolated RNA was then used to synthesis cDNA through a reverse transcription process utilizing a specialized enzyme kit. The qRT-PCR experiment was conducted via the Maxima SYBR Green qRT-PCR kit, with amplification carried out on the Step One PCR Detection System. Expression levels of target genes was quantified applying the comparative CT method using GAPDH as an internal control [39]. The primer sequences, as obtained from NCBI and used in qRT-PCR, are detailed in S1 Table.

#### Enzyme-linked immunosorbent assay (ELISA).

Tyrosine hydroxylase, α-Syn, TLR-4, NF-κB, Sirt-1, and Nrf2 levels were detected in the brain tissues using ELISA commercially available kits.

#### Statistical analysis.

Data were presented as the mean ± SD. The significant differences among the values were analyzed by employing one way ANOVA and Tukey’s multiple comparisons test using Graph Pad version 7 with a statistical significance at *p* < 0.05.

## Results

### LC-HR-ESI-MS profiling (Fig 1) of *T. aphylla* extract

**Fig 1:**
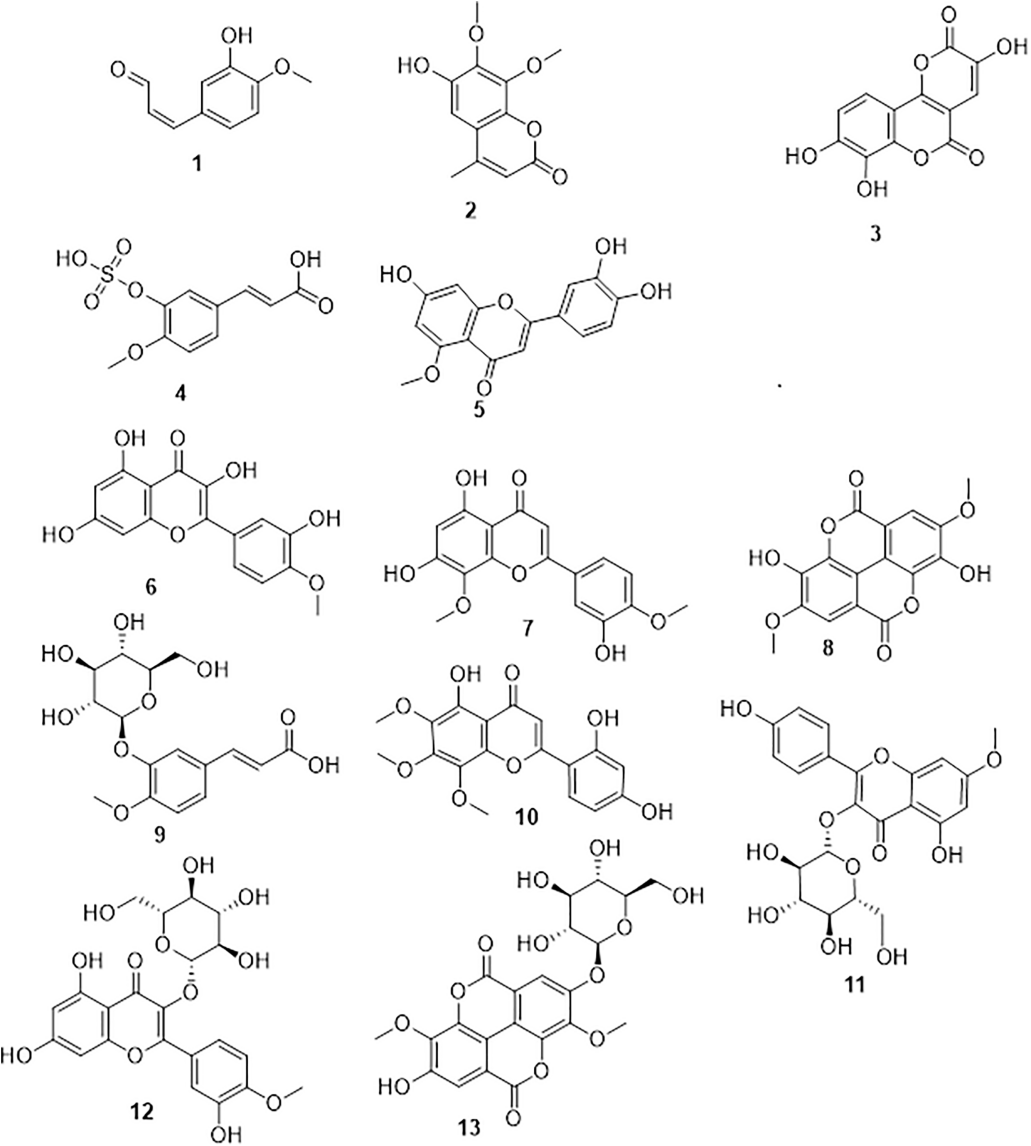
Dereplicated metabolites of *T. aphylla* leaves extract.

Metabolomic analysis was conducted through LC-HR-ESI-MS to analyze the metabolic pool of the crude extract. Various classes of compounds were detected, including flavonoids and tannins, which were the prominent metabolites present in the crude extract (Fig 1, S2 Table). These metabolites were identified tentatively through comparison with the literature, while thirteen compounds were reported.

In this context, the prominent ion peak at *m/z* 177.055 with chemical formula C_10_H_10_O_3_ was recognized as isoferulaldehyde (**1**), isolated earlier from *T. nilotica* [40]. The prominent ion peak at *m/z* 235.060 in accordance with the forecasted chemical formula C_12_H_12_O_5_ was identified as troupin (**2**), which was first reported from *T. troupii* [41]. Further, the mass ion peak of m/z 261.007 having the chemical formula C_12_H_6_O_7_ was recognized to be 3,7,8-trihydroxy-2H,5H-pyrano[3,2-c][1]benzopyran-2,5-dione (**3**) [42]. Further, the chemical formula C_10_H_10_O_7_S, for isoferulic acid-3-sulphate (**4**) was detected from the mass ion peak at *m/z* 273.007; it had, earlier, been detected in the extract of *T. nilotica* [43]. In addition, the mass ion peak at *m/z* 301.070 for the proposed chemical formula C_16_H_12_O_6_ was pinpointed as 3’,4’,7-trihydroxy-5-methoxyflavone (**5**), which had formerly been obtained from *Tamarix* sp. [44]. The mass ion peak at *m/z* 315.050 for the proposed chemical formula C_16_H_12_O_7_ was identified as 3,3’,5,7-tetrahydroxy-4’-methoxyflavone (**6**). This flavonoid had previously been isolated from a *Tamarix* sp. [45]. Moreover, the mass ion peak at *m/z* 329.069, for the predicted formula C_17_H_14_O_7_, was identified as tamaridone (**7**), which had previously been isolated from *T. dioica* [46]. The 2,7-di-methoxylellagic acid (**8**) for the expected molecular formula C_16_H_10_O_8_ was dereplicated from the mass ion peak at *m/z* 329.032; it had earlier been isolated from *T. gallica* [47]. Aphyllin (**9**), with the molecular formula C_16_H_20_O_9_, was dereplicated from the mass ion peak at *m/z* 355.103, which had earlier been characterized from *T. aphylla* [48]. Additionally, the metabolite tamadone (**10**) was dereplicated from its mass ion peak at *m/z* 359.076 following the chemical formula C_18_H_16_O_8_; it had previously been isolated from *T. dioica* [49]. In addition, the metabolite following the chemical formula C_22_H_22_O_11_ and mass ion peak *m/z* 463.123 was identified to be rhamnocitrin 3-glucoside (**11**), a compound isolated from *Tamarix* sp. [50]. Moreover, tamarixin (**12**), with the predicted molecular formula C_22_H_22_O_12_, was dereplicated from the mass ion peak at *m/z* 477.104, it had previously been obtained earlier from *Tamarix* sp. [48]. Similarly, the metabolite ellagic acid 3,3′-dimethyl ether 4-O-*β*-D-glucopyranoside (**13**), with the chemical formula C_22_H_20_O_13_, was dereplicated from the mass ion peak at *m/z* 491.097; this metabolite had earlier been isolated from *T. nilotica* [40].

### Computational studies

#### Therapeutic targets for PD.

A dataset of 65 key proteins linked to PD was meticulously curated from the PharmGKB and NCBI-GEO databases (S3 Table). These proteins include crucial targets such as NLRP3, caspase-1, Sirt-1, and TNF-α, which play pivotal roles in neuroinflammation, oxidative stress, and apoptotic pathways involved in PD. This data set strengthens the relevance of these proteins as therapeutic targets. To further explore therapeutic targets for PD using compounds derived from *T. aphylla*, we employed the SwissTargetPrediction platform. This platform uses an advanced algorithm to predict potential protein targets based on the structural similarities of the dereplicated metabolites of *T. aphylla* (compounds **1**–**13**) to known bioactive molecules. The platform predicted potential interactions between the compounds and several protein targets, as detailed in the Supplementary Materials (S4–S16 Tables). From these predictions, we selected the 55 most relevant proteins associated with PD for further analysis. These identified proteins were incorporated into a protein-protein interaction (PPI) network to better understand their roles and interconnections in neuroprotective processes. This approach allowed us to pinpoint key proteins that could serve as potential therapeutic targets, paving the way for developing neuroprotective strategies against PD using *T. aphylla* secondary metabolites.

#### Protein network construction for PD interaction with dereplicated compounds from *T. aphylla.*

**Fig 2:**
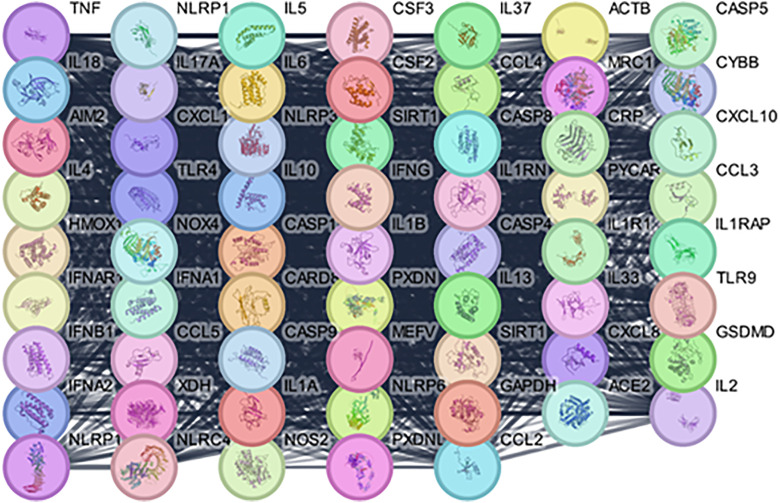
PPI network for identified phytoconstituents from *T. aphylla* against PD.

To construct the protein-protein interaction (PPI) network in our study, proteins related to PD and the identified metabolites in *T. aphylla* were incorporated into the STRING database, version 12.0 (https://string-db.org). This enabled the development of preliminary PPI networks, revealing direct and functional relationships between the compounds and key proteins involved in neuroinflammation, oxidative stress, and apoptosis-critical processes in PD pathology. The PPI network was visualized using Cytoscape software, version 3.10.1. Using the Analyzer feature of Cytoscape, we built a comprehensive protein interaction network including 61 nodes and 1245 interaction linkages, resulting in average node connectivity of 40.82. This network reflected the intricate molecular relationships between key PD markers, such as NLRP3, caspase-1, Sirt-1, and TNF-α, and the metabolites derived from *T. aphylla*. The complexities of this network were described in Fig 2, which highlights the comprehensive analysis of protein interactions related to studying the neuroprotective effects of *T. aphylla* compounds. These insights helped to identify potential therapeutic targets and offer a clearer understanding of the molecular mechanisms where these compounds may exert protective effects against PD.

#### Hub genes identification.

**Fig 3:**
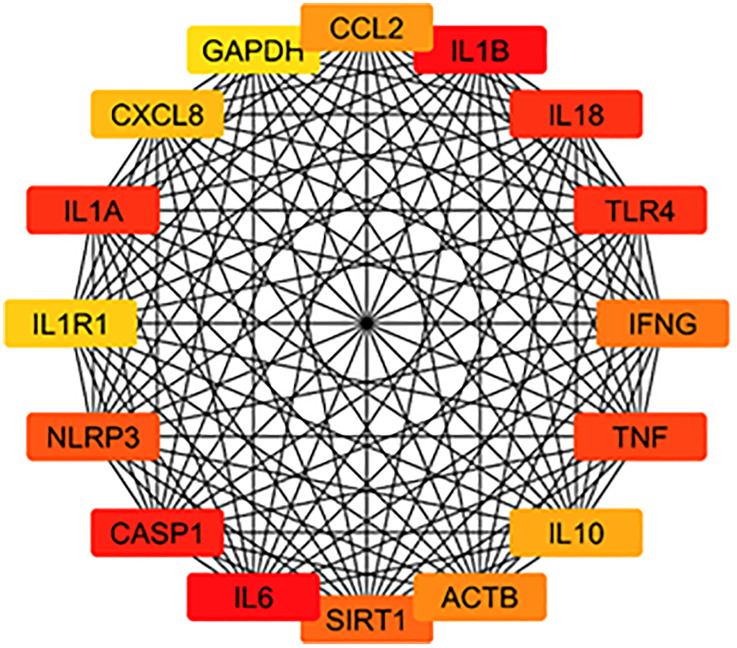
Key hub genes associated with PD.

Using the CytoHubba plugin in Cytoscape, we identified key hub genes within the *P*rotein-*P*rotein interaction (PPI) network related to the neuroprotective effects of identified phytoconstituents from *T. aphylla* in PD. These hub genes, including TLR-4, NLRP3, CASP1, IL1B, IL6, IL18, Sirt-1, TNF, IL10, IFNG, CCL2, CXCL8, IL1A, IL1R1, and ACTB, exhibit high connectivity within the network and of significant importance (Fig 3). These genes are critical in regulating neuroinflammation, apoptosis, and oxidative stress, which are central processes in PD. Key roles include cytokine production (e.g., IL1B, IL6, TNF), immune signaling (e.g., TLR-4, NLRP3), apoptosis regulation (e.g., CASP1, Sirt-1), and actin cytoskeleton organization (e.g., ACTB). The identification of these hub genes provides insight into potential molecular targets for therapeutic intervention in the treatment of PD using compounds from *T. aphylla*.

#### Analysis of overrepresented gene ontology terms.

**Fig 4:**
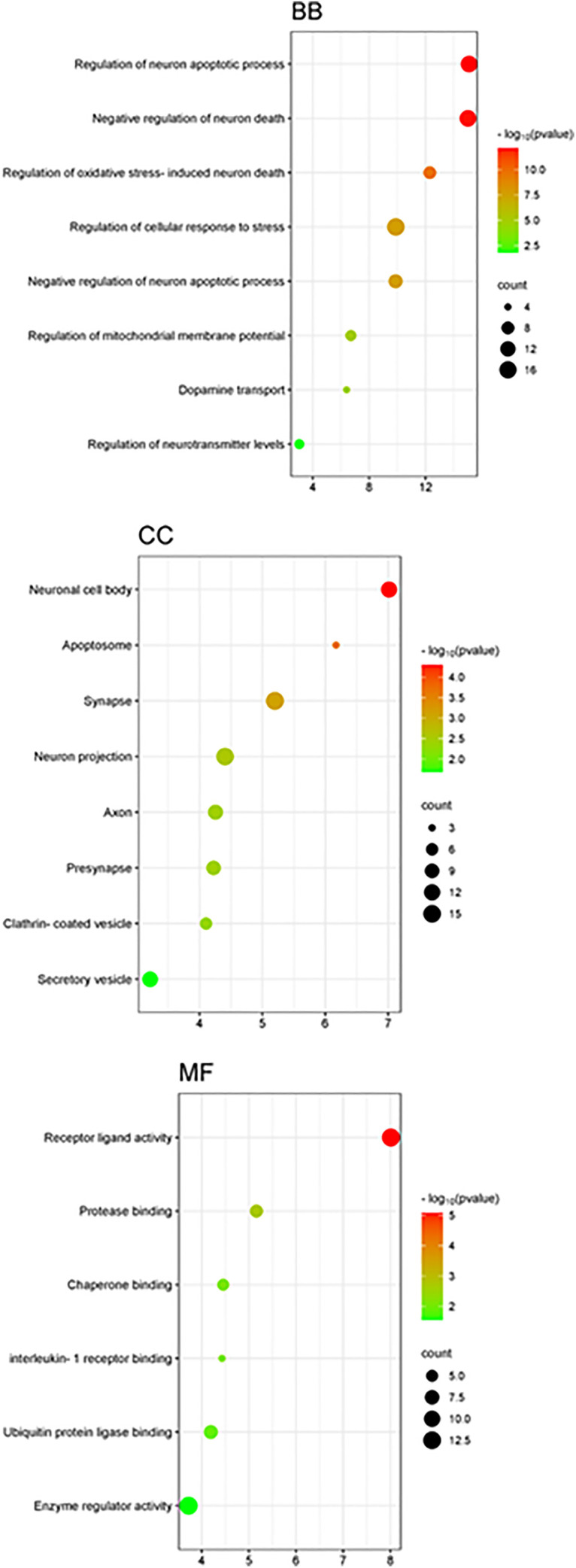
Combined Gene Ontology (GO) enrichment analysis showing the top significant terms across three categories: Biological Process (BB), highlighting neuron survival and stress-response pathways; Cellular Component (CC), emphasizing neuronal structures such as synapse, axon, and cell body; and Molecular Function (MF), illustrating key binding and regulatory activities including receptor–ligand and protease interactions.

The gene ontology (GO) enrichment analysis for our study on the neuroprotective effects of identified phytoconstituents from *T. aphylla* in the context of PD was performed using ShinyGO v0.80. Results indicated significant involvement of proteins in several categories. In the category Biological Process (BP) category (Fig 4), overrepresented terms involve “Positive regulation of interleukin-1 beta production,” “Regulation of NLRP3 inflammasome complex assembly,” “Regulation of I-κB kinase/ NF-κB signaling,” and “ROS metabolic process”. The implicated biological processes are essential for the regulation of inflammation and oxidative stress, both of which are key drivers of neuronal dysfunction and loss in Parkinson’s disease. Notably, dysregulation of NF-κB signaling and activation of inflammasomes are closely associated with heightened neuroinflammatory states and oxidative damage, underscoring their relevance in PD pathogenesis. In the category Cellular Component (CC) (Fig 4), significant terms involve “NLRP3 inflammasome complex”, “Caspase complex,” “Extracellular region,” and “Extracellular space.” These components are associated with key inflammatory processes, particularly the NLRP3 inflammasome, which has been implicated in the neuroinflammatory cascades contributing to dopaminergic neuronal death in PD. The presence of caspase complexes highlights their role in apoptosis, further underscoring the importance of these mechanisms in neurodegeneration. The Molecular Function (MF) enrichment analysis (Fig 4) revealed prominent GO terms such as interleukin-1 receptor binding, cysteine-type endopeptidase activity linked to apoptotic processes, NAD(P)+ nucleosidase activity, and FAD binding. These molecular functions are crucial for modulating inflammatory responses and oxidative stress, both of which are central to PD pathology. The involvement of interleukin-1 signaling and caspase activation suggests a focus on neuroinflammatory pathways and neuronal apoptosis, hallmarks of PD. These GO term analyses align with our aim to delineate the molecular mechanisms through which the dereplicated phytoconstituents from *T. aphylla* confer neuroprotection against PD. The detailed results and their implications are presented in Supplementary S17 Table.[Subxref17]

#### Examination of predominant KEGG pathways.

**Fig 5:**
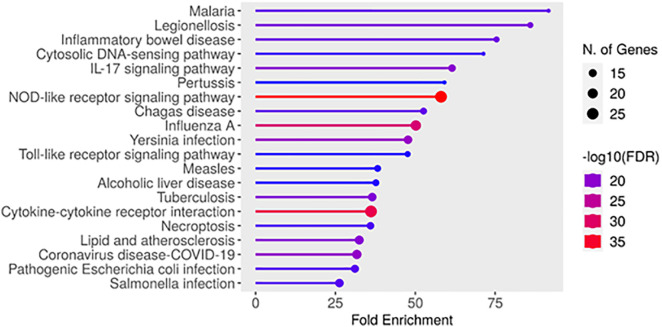
Bar plot illustrates the significant KEGG pathways, enriched by identified phytoconstituents from *T. aphylla*, showcasing their potential neuroprotective effects in PD.

Analysis of KEGG pathways highlighted several significantly enriched signaling routes associated with the neuroprotective activity of *T. aphylla* metabolites in Parkinson’s disease (Fig 2; S18 Table). These include the NOD-like receptor, IL-17, toll-like receptor, and necroptosis pathways, as well as cytokine–cytokine receptor interactions. Each of these is intricately linked to key processes such as neuroinflammation, programmed cell death, and immune modulation, underscoring the therapeutic relevance of the identified compounds. The bar plot in Fig 5 emphasized these enriched pathways and their significant roles in mitigating neurodegeneration and promoting neuroprotection in PD. These pathways also highlighted potential therapeutic targets, offering insights into new strategies for combating neuroinflammation and oxidative stress associated with PD.

**Fig 6:**
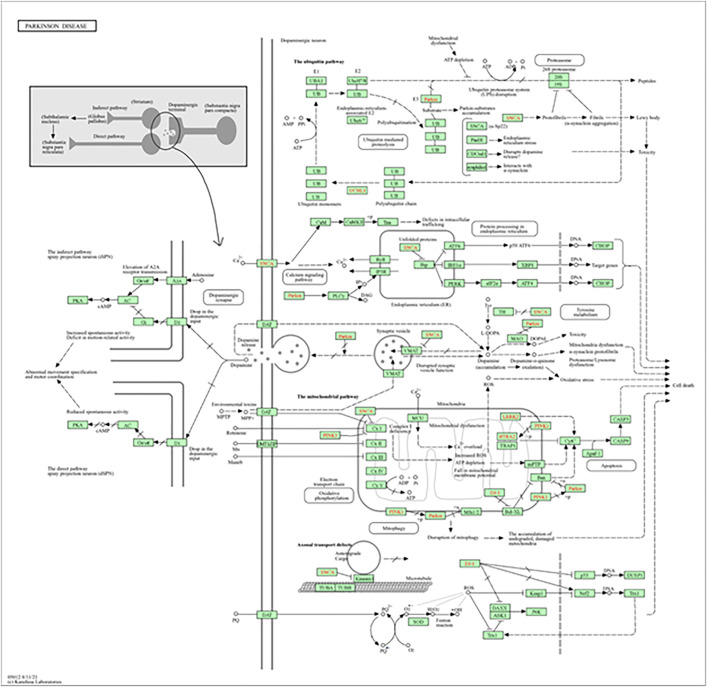
The NOD-like receptor signaling pathway in PD.

Among the examined pathways, the “NOD-like receptor signaling pathway” is particularly significant in the context of neuroinflammation and PD. This pathway outlines how NOD-like receptors (NLRs) interact with cellular components, leading to the activation of inflammatory responses and neurodegenerative processes. Fig 6 outlines the molecular framework of neuroinflammatory progression in PD, emphasizing NLRP3 inflammasome activation as a central trigger for IL-1β and IL-18 release. These cytokines amplify inflammatory signaling, contributing to dopaminergic neuron degeneration. The pathway also captures the involvement of caspases in inflammasome-mediated pyroptosis, linking immune activation to inflammatory cell death. This mechanism is crucial in understanding how chronic neuroinflammation might lead to cell death and disease progression in PD. Furthermore, the NOD-like receptor pathway interacts with the NF-κB signaling cascade, which is essential for regulating immune responses and inflammation. In PD, the dysregulation of NF-κB signaling can lead to persistent inflammatory states, further contributing to neuronal injury. By targeting these critical points within the NOD-like receptor pathway, bioactive compounds from *T. aphylla* could mitigate neuroinflammation and reduce neuronal damage, offering potential therapeutic strategies for neuroprotection in PD. The ability of these compounds to modulate inflammatory responses and interfere with the progression of neurodegenerative processes highlights their therapeutic potential.

#### Molecular modeling with the Sirt-1 catalytic domain (Fig 7).

**Fig 7:**
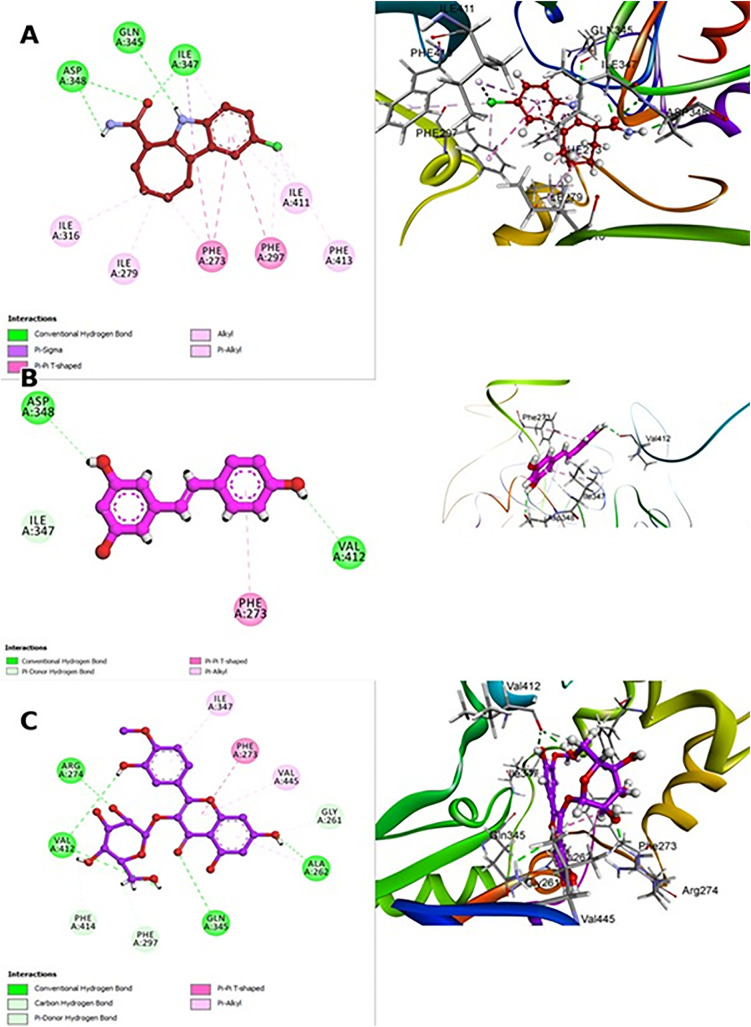
Molecular docking interactions of the co-crystallized ligand (A), resveratrol (B), and compound 12 (tamarixin) (C) with the Sirt-1 catalytic domain. Tamarixin exhibited the highest docking score (–7.98 kcal/mol) and most stable binding configuration (RMSD: 0.70 nm), surpassing both resveratrol and the co-crystallized ligand. These findings support the potential of tamarixin (**12**) as a superior neuroprotective agent targeting Sirt-1.

In this study, molecular docking simulations were performed to evaluate the interactions between dereplicated phytoconstituents from *T. aphylla* and the Sirt-1 catalytic domain, aiming to identify compounds capable of modulating Sirt-1 activity. Sirt-1 (Sirtuin 1), a NAD^+^-dependent deacetylase, is integral in neuroprotection, cellular responses to oxidative stress, and mitochondrial function—processes particularly relevant in the context of PD [51]. By targeting Sirt-1, these compounds could potentially offer neuroprotective effects, making them promising candidates for therapeutic interventions in neurodegenerative diseases like PD. The study aimed to screen various phytoconstituents (compounds **1**−**13**) dereplicated from *T. aphylla* for their ability to bind to the Sirt-1 catalytic domain, with the goal of identifying compounds that can modulate Sirt-1 activity and offer protective effects in the setting of PD (S19 Table). The crystal structure of the Sirt-1 catalytic domain was retrieved from the RCSB Protein Data Bank (PDB ID: 4i5i) and used for molecular docking studies [52]. To validate the docking results and provide a comparative reference, resveratrol was used as a positive control. The docking score for resveratrol was –7.12 kcal/mol, with an RMSD of 0.82 nm, and formed hydrogen bonds with residues including ASP348 and VAL412 (Fig 7b).

In comparison, compound **12**, which is identified as tamarixin (an *O*-methylated flavonoid derivative, molecular formula C₁₆H₁₂O₆), demonstrated a superior docking score of –7.98 kcal/mol and more stable interactions, reflected by an RMSD of 0.70 nm. Compound **12** (tamarixin) appeared as the best hit due to its strong binding affinity, small root mean square deviation (RMSD), and significant interactions with critical active-site residues. These favorable factors indicated a stable binding conformation that enhances its therapeutic potential. Key residues involved in this interaction included GLN345, ILE347, PHE273, and ARG274 (Fig 7c). Key residues within the Sirt-1 active site stabilized the ligand via hydrogen bonding and hydrophobic interactions, both of which played a pivotal role in maintaining high-affinity binding. The 3D molecular model provided a spatial visualization of compound **12** (tamarixin) within the Sirt-1 catalytic domain, showcasing how it aligned with the active site and formed crucial contacts that enhanced its inhibitory potential. This spatial conformation was stabilized through a network of hydrogen bonds (highlighted in green) and Pi-Pi T-shaped interactions (in pink), which contribute to its strong binding affinity. A comparison with the co-crystallized ligand of Sirt-1, also included in the docking study, highlighted the superior performance of compound **12**. The co-crystallized reference ligand achieved a docking score of –6.82 kcal/mol and an RMSD of 0.80; however, compound **12** surpassed it in both binding strength and interaction stability, indicating a potentially more favorable binding profile. The co-crystallized ligand formed hydrogen bonds within the Sirt-1 active site, but these were less robust compared to those formed by compound **12** (Fig 7a). By influencing Sirt-1, compound **12** may promote neuroprotection in PD through improved mitochondrial function, oxidative stress reduction, and enhanced neuronal stability.

The docking study results suggested that compound **12** (tamarixin) has strong potential in modulating Sirt-1 activity than both standard control (resveratrol) and the co-crystallized ligand, providing a promising avenue for further research and drug development.

#### Molecular dynamics for compound 12 with the Sirt-1 catalytic domain.

**Fig 8:**
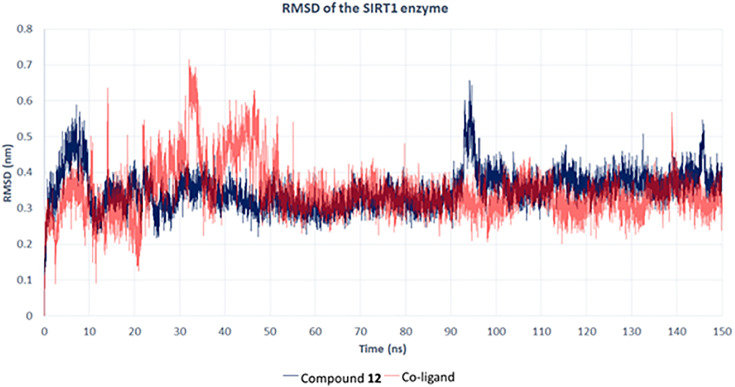
RMSD analysis of compound 12 (tamarixin) with the Sirt-1 catalytic domain, in comparison with the co-ligand.

In this study, we assessed the interaction stability of compound **12** (tamarixin) with the Sirt-1 catalytic domain using molecular dynamics (MD) simulations. Compound **12** (tamarixin) from *T. aphylla* was identified as a promising modulator of Sirt-1 activity through molecular docking studies. MD simulations were used to further evaluate the stability and interaction dynamics of this compound with the Sirt-1 enzyme over a 150-ns simulation period. Root mean square deviation (RMSD) is a key metric used to measure the stability of a protein-ligand complex during the MD simulation. The RMSD values reflect the average displacement of atoms from their initial positions as the simulation progresses, providing insight into how the compound behaves when bound to the target enzyme. The RMSD plot (Fig 8) shows the stability of compound **12** (tamarixin) within the Sirt-1 catalytic domain (blue line), as compared to that of the co-crystallized ligand (red line). RMSD measures the conformational changes in the enzyme-ligand complex over time, providing insights into the binding stability. Throughout the simulation, compound **12** (tamarixin) maintained stable interactions with the enzyme, exhibiting an average RMSD value of about 0.45 nm. The co-crystallized ligand, by contrast, showed higher fluctuations, with an average RMSD value around 0.55 nm, indicating less stability in comparison.

**Fig 9:**
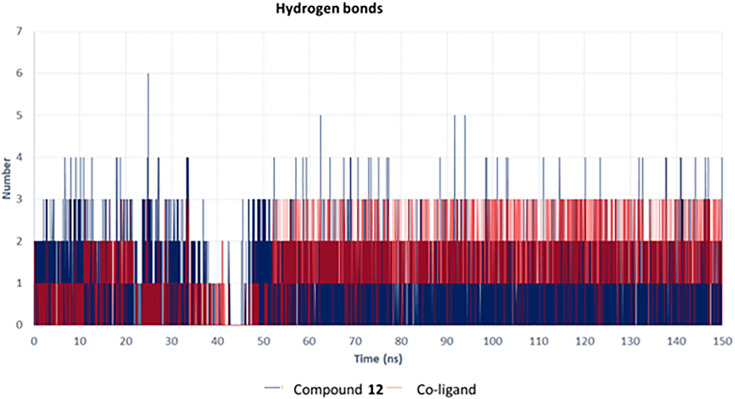
Hydrogen bond analysis of compound 12 (tamarixin) with the Sirt-1 catalytic domain, in comparison with the co-ligand.

Fig 9 presents the hydrogen bonding profile observed during the 150 ns MD simulation, comparing compound **12** (tamarixin) with the native co-crystallized ligand in their interactions with the Sirt-1 catalytic domain. Given the critical role of hydrogen bonds in complex stability and binding strength, the ability of tamarixin to maintain two to six hydrogen bonds throughout the simulation period highlights its enhanced interaction profile and suggests a more stable binding mode relative to the reference ligand. On the contrary, the co-ligand (red) showed fewer hydrogen bonds on average, often fluctuating between one to three bonds. This increased hydrogen bonding by compound **12** (tamarixin) aligned well with the predictions from our initial docking studies, where multiple hydrogen bonds were predicted with critical residues such as ARG274, ALA262, and GLN345.

**Fig 10:**
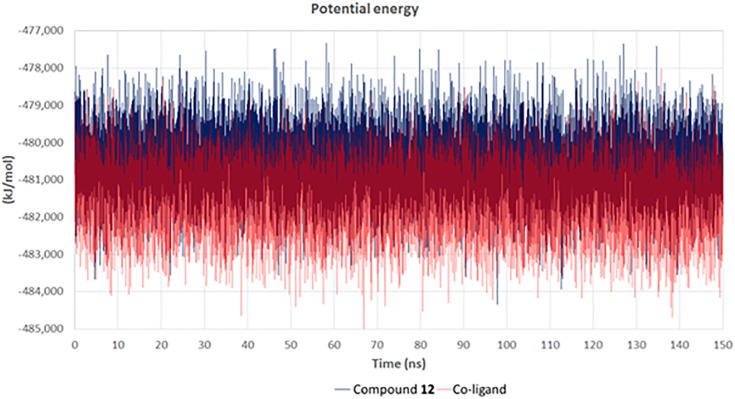
Potential energy analysis of compound 12 (tamarixin) with the Sirt-1 catalytic domain.

Fig 10 illustrates the potential energy trajectory, serving as an indicator of system equilibration and structural stability over the course of the molecular dynamics simulation. The simulation, which ran for 150 ns, showed the potential energy values for compound **12** (tamarixin) (blue) and the co-ligand (red) bound to the Sirt-1 catalytic domain. Throughout the simulation, compound **12** (tamarixin) maintained a relatively stable potential energy profile, with values hovering consistently between −479,000 and −483,000 kJ/mol. The stable energy values indicated that the binding of compound **12** (tamarixin) to the Sirt-1 catalytic domain did not disrupt the stability of the system, suggesting a favorable interaction. The co-ligand, while also showing stability, had a slightly more fluctuating potential energy profile, which might indicate a less stable binding compared to compound **12** (tamarixin).

The stability in MD analysis observed in compound **12** (tamarixin) reinforced the docking analysis, where compound **12** (tamarixin) was found to have a strong affinity and stable interactions within the active site of the Sirt-1 catalytic domain. These findings supported the hypothesis that compound **12** (tamarixin) could effectively modulate Sirt-1 activity, offering potential neuroprotective effects, particularly in the context of PD.

#### Histopathological examination.

**Fig 11:**
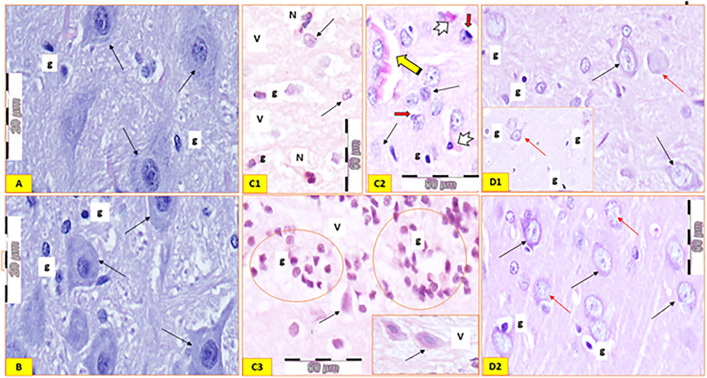
Histological findings of H&E-stained corpus striatum “CS” of all studied groups: GI, A), Control group; showing cluster of branched multipolar neurons with moderate-dense basophilic cytoplasm, large pale nuclei and infrequently branching dendrites (linear arrows). Neuroglia cells (g). GII, B), (extract *Tamarix* only); showing neurons with normal picture of intense basophilic cytoplasm and large vesicular nuclei (linear arrows). Neuroglia cells (g). GIII, C1 & 2 &3), Parkinsonism group; Many neurons appear shrunken surrounded by perineuronal hallows (arrows). Notice the increased interstitial spaces, dilated blood vessels (stripped arrow) and aggregation of acidophilic Lewy bodies (tailed arrows). The neuropil has many vacuoles (V) and inflammatory cells mainly neutrophils (N). Large focal nodules of neuroglial cells (gliosis) are clearly seen (empty circles). G IV, D1 & D2), (rotenone + *Tamarix* extract): CS tissue sections showing numerous neuronal normal pictures of moderate to intense basophilic cytoplasm and vesicular nuclei (linear black arrows) while other cells appear shrunken with small dense nuclei and surrounded by empty hallows (linear yellow arrows). Neuroglia cells (g). (H&E staining 1000 & 400).

Using H&E, the *corpus striatum* of the control rats had a striped appearance formed primarily of interconnected neurons cluster and diverging fibers. The neurons appeared multipolar or bipolar, with occasionally branching dendrites, with the remaining other types of interneurons. Their cytoplasm was moderate to intense basophilic, with large pale nuclei appearing almost rounded. Intercellular neuropils showed neuroglia cells that had small sizes and darker nuclei (Fig 11A). CS tissue sections of group 2 rats stained with H&E appeared roughly matched the image of the control group (Fig 11B). Tissue sections of the rotenone group revealed more interstitial gaps between the neurons, which varied in size but mostly were shrunken and deeply stained but most of them had darkly stained nuclei and pale cytoplasm indicating chromatolysis. Neuronal processes also seemed to be retracted or deleted (Fig 11 C1). Peri-neuronal hallows surrounded several shrinking neurons. Nonetheless, a few viable neurons with basophilic cytoplasm and weakly stained nuclei were to be seen. Additionally observed were inflammatory cells with pigmented substances, such as neutrophils and macrophages (Fig 11 C2). The primary neurodegenerative characteristics of the neuropil were vacuolations, aggregates of eosinophilic, rounded, and elongated bodies (Lewy bodies) and large focal nodules of neuroglial cells (gliosis) were clearly observed (Fig 11 C2 and C3). The rotenone + *Tamarix* treated group revealed marked improvement of the histological picture with many neurons regaining their granular basophilic cytoplasm, rounded pale and conspicuous nucleoli, but others remained shrunken, with small dense nuclei and empty hallows around them. Neuroglial cells seemed to have increased (Fig 11; D1 and D2).

#### *T. aphylla* protects dopaminergic neurons from rotenone-induced neurodegeneration.

**Fig 12:**
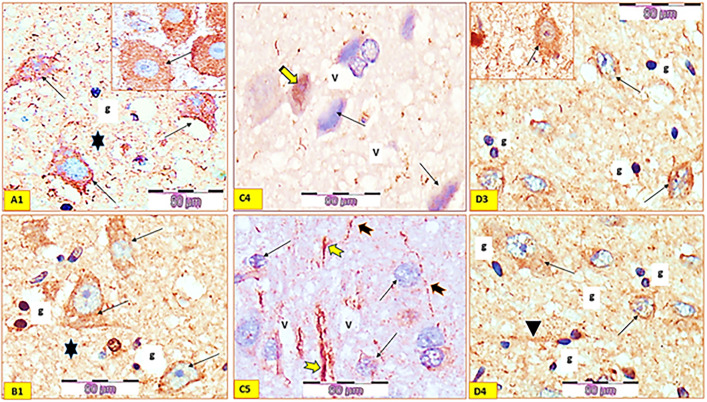
Histological findings of tyrosine hydroxylase (TH) immune stained tissue sections of corpus striatum “CS” of all studied groups: GI, A1), control group; showing cluster of strong positive immune-reactive neurons (linear arrows) among the predominant interneurons (stars). (GII, B1), (extract *Tamarix* only); showing strong positive immune-reactive neurons among the predominant interneurons (arrows). (GIII, C4 & 5), Parkinsonism group; neurons showing very faint immunoreactivity (linear arrows) and some neurons appear with peri-nuclear aggregates of dense inclusions (stripped arrow). Beaded (tailed black arrows) or swollen processes (tailed yellow arrows) and vacuolated neuropil (V) are seen. G IV, D3 & D4), (rotenone + *Tamarix* extract). showing strong cytoplasmic expression of immune-reactivity and mild immune-reactive neuropil in comparison with the control group. The neuropil showing enlarged nerve cell processes (arrow head) (tyrosine hydroxylase immune-staining x400).

We used TH immunohistochemistry on experimental animal brain slices to determine whether *T. aphylla* may shield dopaminergic neurons from rotenone damage. In the *corpus striatum* of the control group, TH staining revealed a cluster of very positive TH immune-reactive neurons (Fig 12 A1). CS tissue slices TH immune-staining showed much the same image as the control group in the *Tamarix-*alone group (Fig 12 B). TH immunostaining of CS of the rotenone group showed a number of pathological changes, e.g., very faint immunoreactivity and the immune-positive neurons were rarely seen. Additionally, beaded or enlarged processes and perinuclear clusters of dense inclusions were seen inside the immune-positive neurons. Neuropil had some regions with reduction in the intensity of the immunoreactivity and other negative immunoreactive areas. Also, dilated blood vessels and multiple vacuolations in the neuropil were observed (Fig 12 C4, C5). TH-immunostaining of group 4 showed strong cytoplasmic expression of immune-reactivity and mild immune-reactive neuropil compared to the control group. Enlarged nerve cell processes looked diffusely distributed alongside the somatic-dendritic compartment (Fig 12; D3, 4).

#### Effect of *T. aphylla* on α-Syn and TH expression.

**Fig 13:**
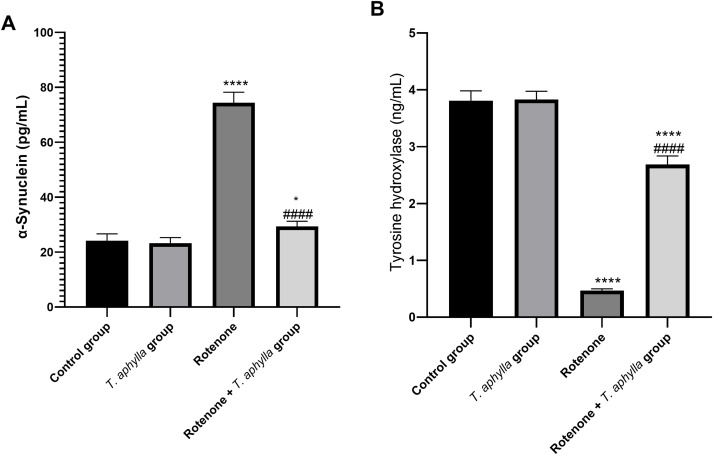
Assessment of (A): α-synuclein and (B): tyrosine hydroxylase in the midbrain rats. ANOVA test was used, followed by Tukey’s post hoc test. N = 6. Values are expressed as mean ± SD. * *p* < 0.05 compared to control, # *p* < 0.05 compared to the rotenone-treated group.

ELISA was conducted to evaluate the existence of α-Syn, a distinctive marker of PD, within the experimental cohorts. Administration of rotenone via injections notably escalated the α-Syn levels to 74.4 pg/mL, in contrast to the control rat levels (Fig 13A). Conversely, treatment with *T. aphylla* demonstrated a reduction in α-Syn expression compared to the rotenone-exposed group, with levels measuring at 29.3 pg/mL.

Also, the level of tyrosine hydroxylase was assessed using ELISA. As presented in Fig 13B, its level was markedly suppressed (0.47 ng/mL) in rotenone-treated rats. On the contrary, the level of tyrosine hydroxylase was markedly elevated in *T. aphylla* treated rats (2.86 ng/mL).

#### Administration of *T. aphylla* contracted lipid peroxidation and enhanced GSH, SOD, and CAT levels in rotenone-treated rats.

**Fig 14:**
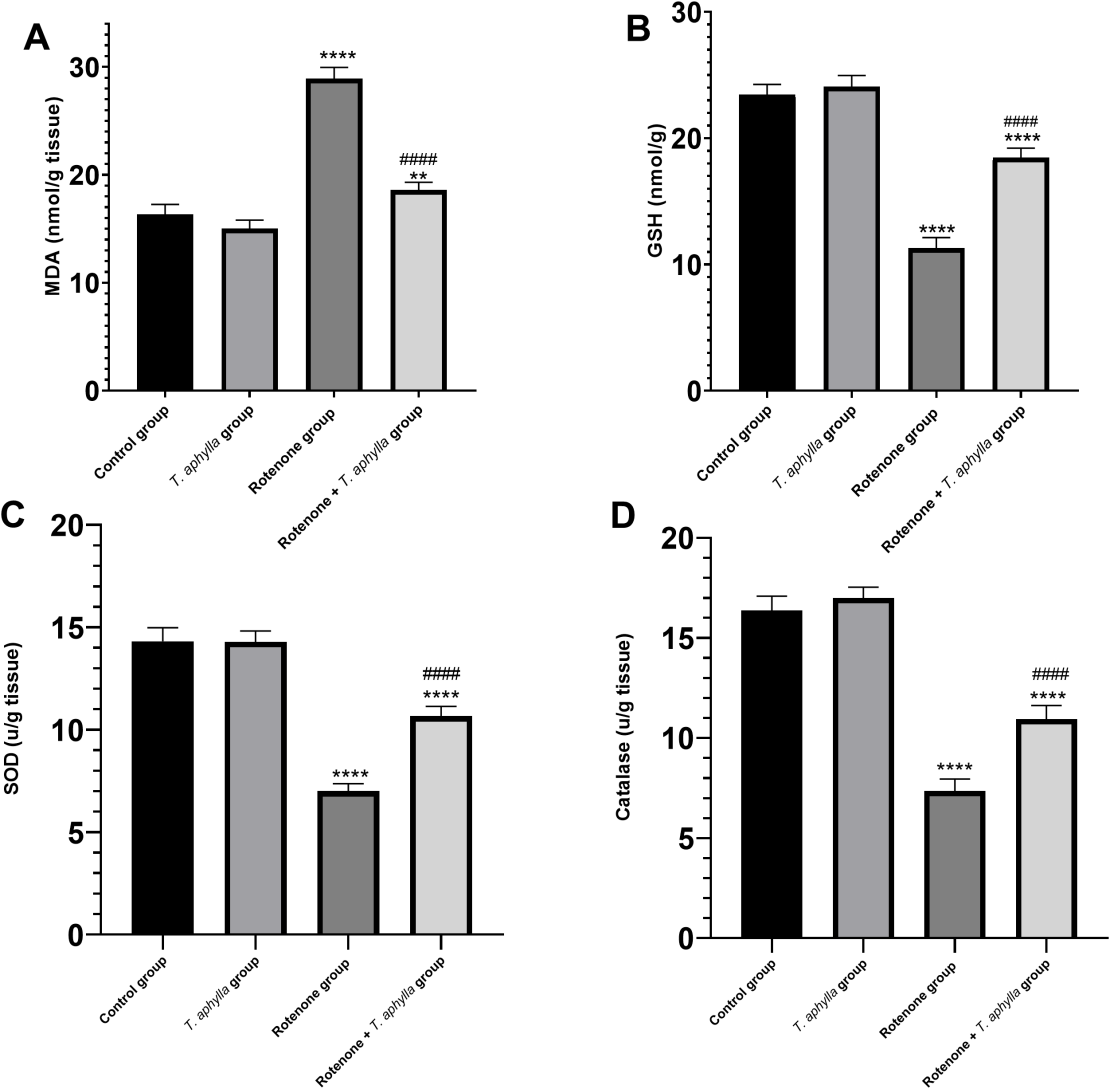
Assessment of (A): MDA, (B): GSH, (C): SOD, and (D): CAT in the midbrain rats. ANOVA test was used, followed by Tukey’s post hoc test. N = 6. Data are expressed as mean ± SD. * *p* < 0.05 compared to control, # *p* < 0.05 compared to the rotenone-treated group.

Evaluation of lipid peroxidation through the measurement of MDA levels stands as a crucial biochemical parameter for delineating the pathogenic cascade underlying oxidative stress-induced cellular dysfunction. Administration of rotenone induced a notable (*p* < 0.05) elevation in MDA concentrations in rotenone-treated rats (28.9 nmol/g tissue) in comparison to the untreated control rats (Fig 14A). Concurrently, rotenone exposure also led to a noteworthy (*p* < 0.05) decline in GSH levels (11.3 nmol/g) (Fig 14B). Administration of *T. aphylla*, by contrast, markedly (*p* < 0.05) ameliorated lipid peroxidation (MDA: 18.6 nmol/g tissue) and bolstered GSH levels (18.45 nmol/g) when compared to animals exposed to rotenone.

To explore the antioxidant potential of *T. aphylla*, we evaluated the levels of SOD as well as CAT. Our data unveiled that rotenone significantly (*p* < 0.05) escalated oxidative stress, evidenced through marked reduction in SOD (7 U/g tissue) and CAT (7.35 U/g tissue) levels relative to the control group. However, treatment with *T. aphylla* substantially (*p* < 0.05) elevated SOD (10.67 U/g tissue) (Fig 14C) and CAT (11 U/g tissue) (Fig 14D) activities in comparison to rotenone-exposed subjects.

#### *T. aphylla* suppresses expression of pro-inflammatory cytokines.

**Fig 15:**
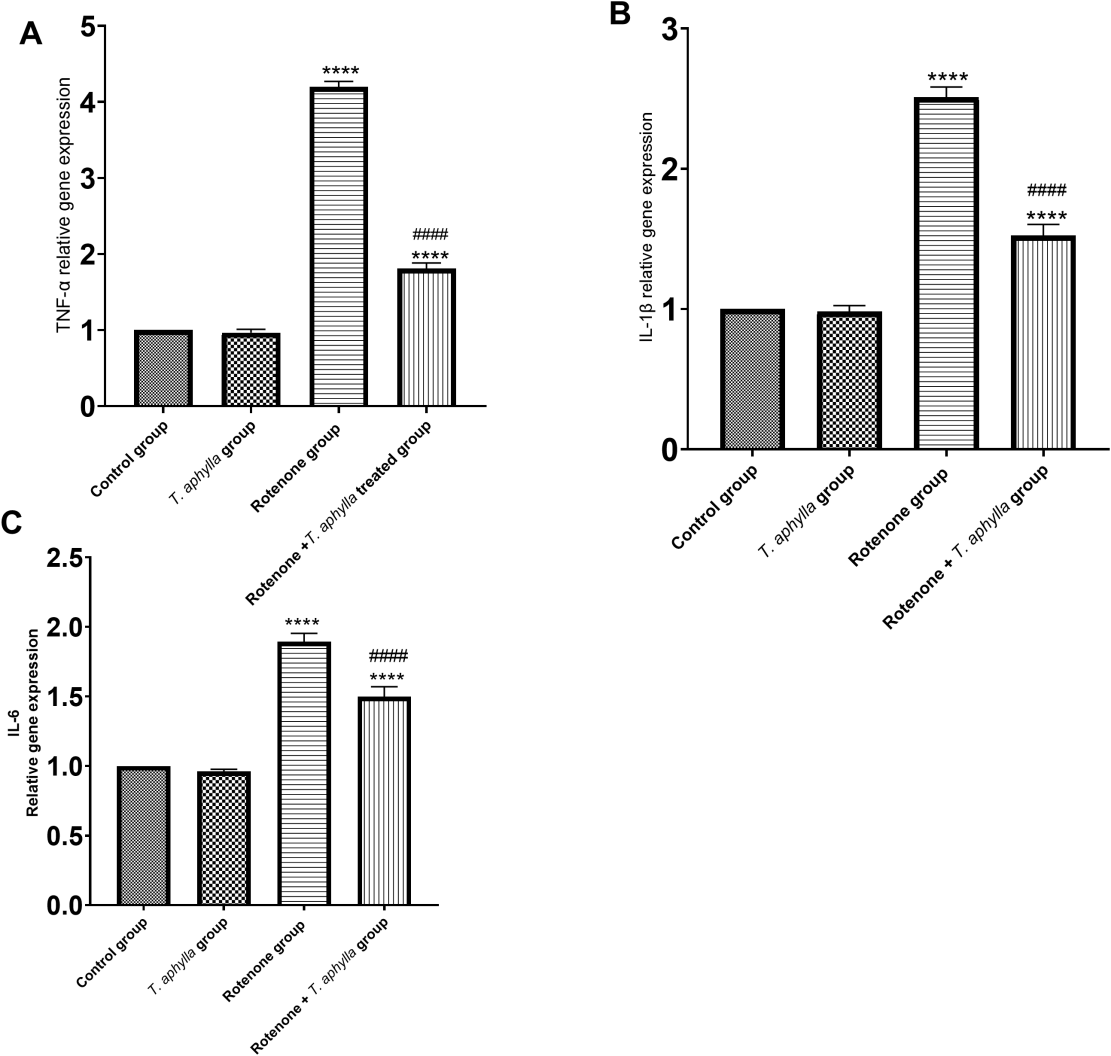
Real-time PCR analysis of (A): *TNF-α*, (B): *IL-1β* and (C): *IL-6* gene expression in experimental animals. Expressions were calculated relative to the control sample after normalizing GAPDH (housekeeping gene). ANOVA test was used, followed by Tukey’s post hoc test. N = 6. Data are expressed as mean ± SD. * *p* < 0.05 compared to control, # *p* < 0.05 compared to the rotenone-treated group.

*TNF-α, IL-1β* as well as *IL-6* serve as pivotal mediators in the initiation and perpetuation of inflammation. To elucidate the impact of these proinflammatory cytokines, we scrutinized their levels in the experimental subjects. Following rotenone injection, a surge in *TNF-α* (4.2-fold change), *IL-1β* (2.5-fold change), and *IL-6* (1.89-fold change) (Fig 15A–C) was observed within the brain tissues. Intriguingly, treatment with *T. aphylla* in rotenone-exposed animals significantly attenuated (*p* < 0.05) the levels of *TNF-α* (1.81-fold change), *IL-1β* (1.52-fold change), and *IL-6* (1.49-fold change) in the rotenone-treated cohort.

#### Effect of *T. aphylla* on the Sirt-1/Nrf2 pathway.

**Fig 16:**
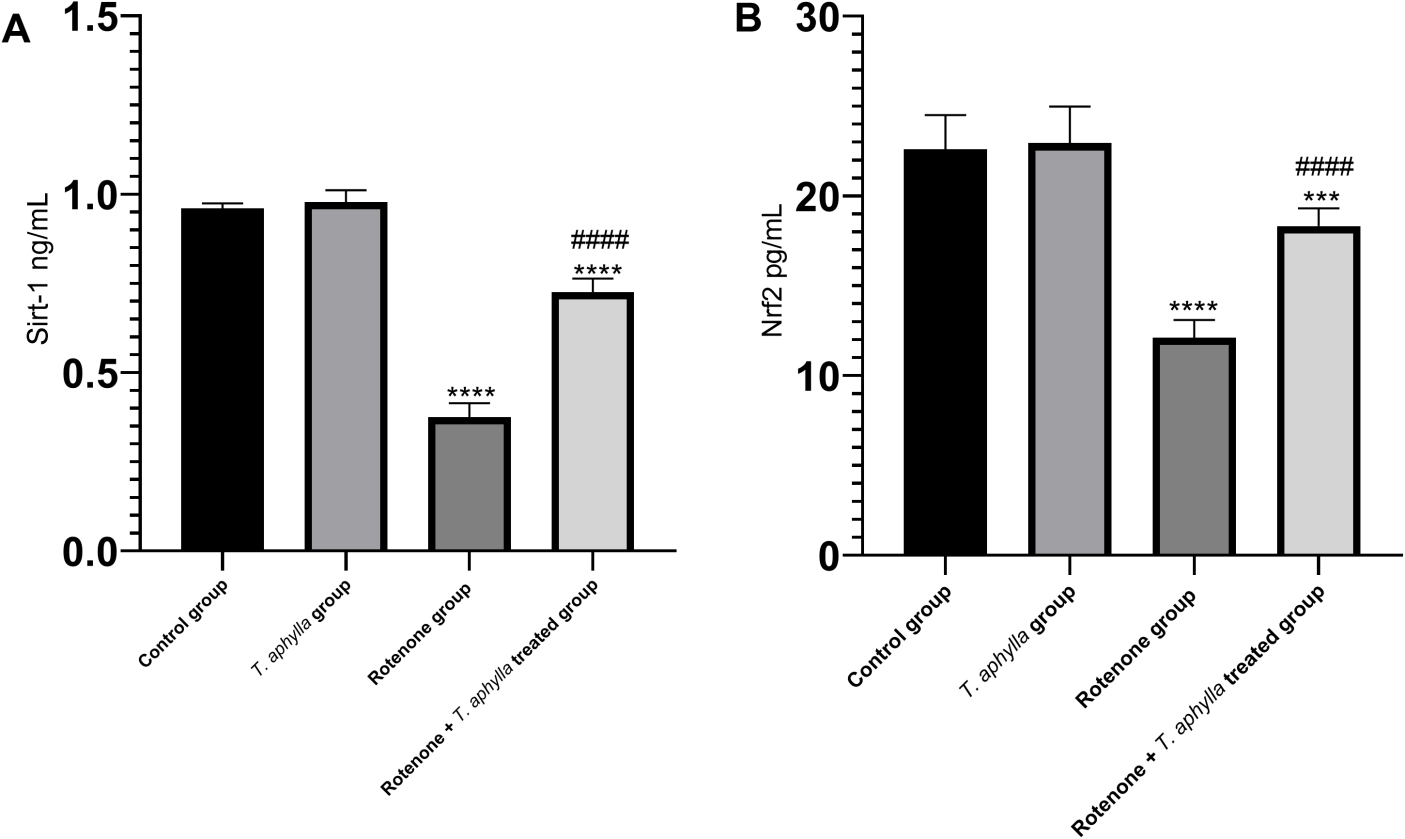
Assessment of (A): Sirt-1 and (B): Nrf2 in experimental groups. ANOVA test was used, followed by Tukey’s post hoc test. N = 6. Values are expressed as mean ± SD. *** *p* < 0.001, **** *p* < 0.0001 compared to control, #### *p* < 0.0001 compared to the rotenone-treated group.

The influence of *T. aphylla* on the oxidative disturbances induced by rotenone was assessed by investigating the changes in the Sirt-1Nrf2 pathway. In contrast to the control rats, the tissues of rats exposed to rotenone displayed a significant exacerbation of oxidative imbalances. This was notably characterized by a marked decrease (*p* < 0.05) in the levels of Sirt-1 and Nrf2 compared to the control cohort, as illustrated in Fig 16. Interestingly, the introduction of *T. aphylla* led to a remarkable improvement in Sirt-1 and Nrf2 expression (*p *< 0.05) in comparison to the group exposed to rotenone.

#### Effect of *T. aphylla* on the TLR-4/NF-κB pathway.

**Fig 17:**
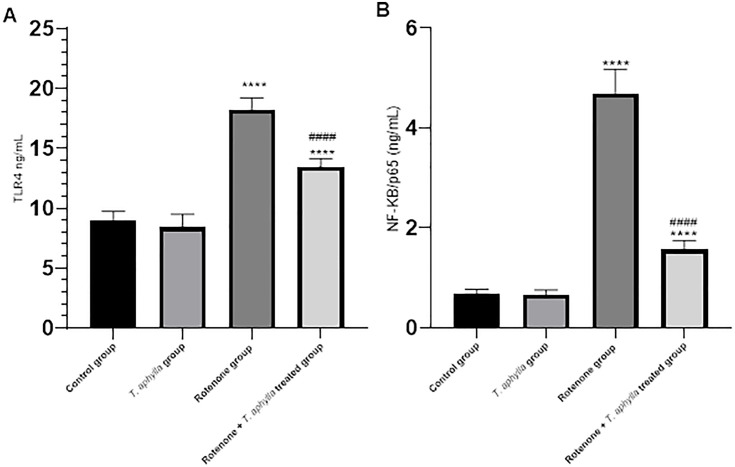
Assessment of TLR-4 and NF-κB. A one-way ANOVA test was used, followed by Tukey’s post hoc test. N = 6. Values are expressed as mean ± SD. **** *p* < 0.0001 compared to control, #### *p* < 0.0001 compared to the rotenone-treated group.

The influence of *T. aphylla* on the TLR-4/NF-κB pathway precipitated by rotenone was evaluated. Relative to control animals, the tissues of rotenone-treated rats displayed marked aggravation of the TLR-4/NF-κB pathway. This was apparent through an extensive increase (*p* < 0.05) in TLR-4 (18.2 ng/mL) and NF-κBp65 (4.65 ng/mL), relative to control animals as delineated in Fig 17. Concomitantly, the treatment with *T. aphylla* elicited a noteworthy augmentation in the TLR-4 (13.4 ng/mL) and NF-κB (1.55 ng/mL) (*p* < 0.05) expressions when compared to the rotenone-treated rats.

#### Effect of *T. aphylla* on pro- and anti-apoptotic markers.

**Fig 18:**
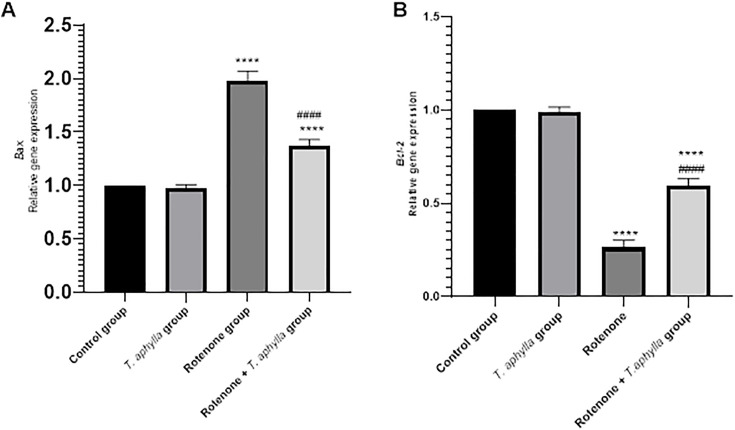
Real-time PCR analysis of (A): *Bax* and (B): *Bcl-2* gene expression in experimental animals. Expressions were calculated relative to the control sample after normalizing to GAPDH (housekeeping gene). ANOVA test was used, followed by Tukey’s post hoc test. N = 6. Values are expressed as mean ± SD. **** *p* < 0.0001 compared to the control, #### *p* < 0.0001 compared to the rotenone-treated group.

Increased oxidative stress provokes dopaminergic neurons apoptosis serving as the primary mechanism underlying neuronal degeneration in PD. We studied the expression of apoptotic and antiapoptotic genes (*Bax* and *Bcl-2*) and the change induced following rotenone or *T. aphylla* treatment. Administering rotenone to rats revealed a noteworthy (*p* < 0.05) upsurge in *Bax* gene expression (1.98-fold change) compared to untreated control rats, as illustrated in Fig 18. Conversely, treatment with *T. aphylla* yielded a marked (*p* < 0.05) reduction in *Bax* expression (1.37-fold change) relative to rats solely exposed to rotenone. Strikingly, *Bcl-2*, the antiapoptotic guardian, exhibited a decline in expression in rotenone-exposed rats (0.26-fold change) but experienced an increase post *T. aphylla* treatment (0.6-fold change). Notably, the expression profiles of these proteins stayed unaltered in control and *T. aphylla*-treated rats without rotenone exposure, as depicted in Fig 18.

## Discussion

While there is a plethora of therapies for the treatment PD, the quest persists for an ideal pharmaceutical agent capable not only of stalling the progression of dopaminergic neuronal degeneration but also of affecting a respite from the clutches of the ailment. Despite the initial efficacy demonstrated by various treatment modalities, their extended usage often precipitates debilitating side effects, underscoring the imperative need for innovative drugs boasting a reduced side effect profile to revolutionize the landscape of PD therapeutics [53]. Rotenone, a profoundly lipophilic compound, stands out for its capacity to induce PD in rodent models, mirroring symptomatic manifestations akin to those observed in human patients [54,55]. Noteworthy studies have underscored the potent antioxidant properties exhibited by *T. aphylla* [22].

Nevertheless, the uncharted territory concerning the neuroprotective prowess of *T. aphylla* in an *in-vivo* setting beckons for exploration. Leveraging rotenone-induced PD models as a cornerstone for unraveling the intricate pathophysiological underpinnings of the disease and identifying promising therapeutic agents [56], our study embarks on a quest to probe the neuroprotective efficacy of *T. aphylla* within the framework of a rotenone-induced PD rat model.

The paramount revelation of the current investigation lies in the reversal of rotenone-triggered degeneration of tyrosine hydroxylase (TH) positive neurons within the *substantia nigra pars* *compacta* (SNpc) through the therapeutic intervention of *T. aphylla.* TH, a pivotal enzyme governing dopamine synthesis, catalyzes the conversion of L-tyrosine to L-dihydroxyphenylalanine (L-DOPA), a critical step in dopamine production [57]. Notably, in rotenone-induced lesion models, a conspicuous dearth of TH-immunoreactive dopaminergic neuron fibers has been documented [58]. In our study, rotenone administration precipitated a marked suppression of TH immunostaining within the SN. Conversely, histological scrutiny unveiled a complete preservation of TH immunoreactivity within the SN of lesioned subjects following treatment with *T. aphylla.*

α-Syn, a soluble protein highly concentrated within neuronal presynaptic terminals, features prominently in the constitution of Lewy bodies and Lewy neurites, hallmark pathological structures synonymous with PD [59]. The aggregation of α-Syn into intracellular filamentous assemblies represents a defining pathological trait observed in both sporadic and hereditary forms of PD. The accrual of α-Syn within dopaminergic neurons precipitates diminished activity of mitochondrial complex I, heightened ROS generation, culminating in cellular demise [60]. Studies have reported that rotenone administration in animal models triggers the aggregation of α-Syn into Lewy bodies within surviving SN neurons [16], a process implicated in promoting neuronal demise via necrotic or apoptotic pathways [61]. Our investigation revealed that rotenone exposure elicited an escalation in α-Syn levels. Remarkably, treatment with *T. aphylla* exhibited a repressive effect on rotenone-induced α-Syn accumulation within the SN, underscoring its potential as a therapeutic intervention in mitigating α-Syn pathology.

Oxidative stress emerges as a critical determinant in the progression and pathogenesis of PD, as illustrated by a nexus of studies [62]. This oxidative assault, predominantly targeting neuronal lipids [63], proteins, and nucleic acids, manifests profoundly in the cerebral milieu of PD sufferers owing to the rampant generation of free radicals and ROS [64,65]. The brain, distinguished by its enriched lipid reservoirs, heightened oxygen consumption, and modest antioxidant armamentarium compared to other tissues, stands particularly vulnerable to oxidative insults [66,67]. Perturbations in the intricate machinery of the mitochondrial electron transport chain, notably complex I, can incite a cataclysmic surge in free radical production culminating in cellular demise [68]. Experimental perturbation via systemic rotenone administration, a recognized complex I antagonist, elicits a decline in ATP synthesis, thereby exacerbating ROS production [69]. ROS, in turn, catalyze the oxidation of polyunsaturated fatty acids, a cascade known as lipid peroxidation. MDA, a pivotal byproduct of this process, forms adducts with proteins and DNA bases, instigating a chain of events culminating in cellular injury and dysfunction [70]. In this investigative context, we probed the potential neuroprotective prowess of *T. aphylla* against ROS-induced neuronal impairment in rotenone-exposed rats, gauging the levels of MDA, SOD, CAT, and GSH. Remarkably heightened MDA levels were discerned in rotenone-treated rats compared to controls. Strikingly, *T. aphylla* intervention ameliorated MDA levels in contrast to their rotenone-exposed counterparts. The abatement of lipid peroxidation observed in this study following *T. aphylla* treatment is suggestive of its capacity to neutralize peroxy radicals and ROS, thereby fortifying the antioxidant profile attributed to *T. aphylla*.

GSH, an indispensable antioxidant, has an essential role in the interception of hydrogen peroxide. The surge in MDA levels resultant from oxidative stress is intricately linked to the decline in GSH availability within the cerebral milieu. Consequently, diminished GSH concentrations in the brain may serve as a hallmark of oxidative stress. Notably, Pearce and colleagues noted depleted GSH levels in the brain of PD patients [71]. In our present investigation, a significant reduction in GSH levels was evident in rats injected with rotenone compared to control subjects. Conversely, treatment with *T. aphylla* conspicuously bolstered GSH levels, underscoring the potent antioxidant efficacy of this botanical agent, a phenomenon corroborated by existing literature [72]. The impact of *T. aphylla* on GSH levels may stem from its direct antioxidant properties or its ability to avert rotenone-induced GSH oxidation, thereby accentuating its role in combating oxidative stress.

SOD stands out as a key player among the antioxidant enzymes crucial for combating free radicals [73]. Diminished CAT or SOD functionality exacerbates oxidative stress in rats subjected to rotenone exposure, as evidenced by elevated MDA levels and diminished GSH concentrations. Disruption of the electron transport chain prompts an overwhelming surge in superoxide radicals [74] amplifying the significance of maintaining adequate SOD activity to counteract this surge effectively. CAT, another indispensable antioxidant enzyme, plays a pivotal role in ameliorating oxidative stress by converting cellular hydrogen peroxide into innocuous water molecules [75]. Our current findings unveil reduced SOD and CAT activities in rotenone-treated rats compared to controls. Interestingly, administration of *T. aphylla* elicited a potentiation of SOD and CAT activities in the rotenone-exposed cohort, thereby fortifying the antioxidant defense mechanisms. This observation aligns with previous research indicating an enhancement in SOD activity following *T. aphylla* supplementation [76].

The accumulation of α-Syn in the brain triggers a cascade of events leading to ROS production and oxidative stress, pivotal factors in the degeneration of dopaminergic neurons in PD [60]. Within this intricate landscape, the antioxidant defense system operates through a network of pathways that respond to oxidative challenges. Notably, Sirt-1 emerges as a central regulator, orchestrating processes linked to inflammation, apoptosis, and antioxidant defenses [77]. Studies have underscored the role of Sirt-1 in activating the Nrf2 pathway, a critical mechanism for curtailing ROS generation [78]. Nrf2, a key sentinel of oxidative stress, assumes a primary role in shielding cells from the brunt of excessive oxidative burden [79]. By modulating the expression of various antioxidant defense genes, Nrf2 orchestrates an array of protective mechanisms such as the removal of toxic heme, carbon monoxide, and iron ions. Our investigations reveal that rotenone treatment markedly diminishes the levels of Sirt-1 and Nrf2, indicative of heightened oxidative stress. These findings resonate with prior studies highlighting the potential of rotenone in exacerbating oxidative stress within brain tissues and impeding the expression of Sirt-1 and Nrf2. Conversely, treatment with *T. aphylla* robustly enhances the expression of Sirt-1 and Nrf2 in rotenone-exposed rats, hinting at the antioxidant potential of *T. aphylla* in mitigating rotenone-induced neurodegeneration. These outcomes dovetail with earlier research showcasing the antioxidant prowess of *T. aphylla* in modulating the Sirt-1/Nrf2 pathway [22].

Multiple studies substantiate the notion that inflammation is a key factor in the pathogenesis of PD. In rotenone-treated rats, the neuroinflammatory cascade is set in motion and sustained through diverse mechanisms [80]. The dysfunction of mitochondrial respiratory chain complex I triggered by rotenone administration culminates in a notable surge of ROS, capable of activating glial cells [81]. Extensive research has underscored the involvement of neuroinflammation in PD, often typified by the activation of glial cells [82]. Notably, reports have highlighted the activation of NF-κB in the SNpc of PD patients, MPTP-treated mice [83], and rotenone-exposed rats [80]. Following activation, NF-κB translocates to the nucleus, stimulating the expression of various proinflammatory factors, *viz*. *TNF-α, IL-6*, and *IL-1β*, by microglia [84]. In our current investigation, an elevation in NF-κB expression was noted in rotenone-treated rats, a phenomenon typically associated with neurodestructive processes. Interestingly, *T. aphylla* treatment resulted in a noteworthy lessening in NF-κB expression, indicative of its capacity to downregulate NF-κB gene expression and counteract the inflammatory cascade in PD. Consistent with this, elevated levels of pro-inflammatory cytokines, including TNF-α, IL-6, and IL-1β, were detected in the rotenone-exposed group, aligning with prior studies documenting similar upregulation in rotenone-treated rats [83]. Conversely, *T. aphylla* intervention thwarted rotenone-induced neuroinflammation, as evidenced by the attenuation of *TNF-α, IL-6,* and *IL-1β* expression. The anti-inflammatory attributes of *T. aphylla* could be due to its rich content of a diverse pool of metabolites, such as terpenoids and sterols [85].

*Bcl-2*, a crucial transmembrane protein predominantly situated on the outer mitochondrial membrane, exerts a crucial role in regulating cellular fate by impeding apoptosis triggered by diverse stimuli [86]. Conversely, *Bax* serves as a pro-apoptotic mediator, and caspase-3 functions as an executor during the culminating phases of apoptosis [59]. Elevated levels of ROS possess the capacity to instigate the initiation of controlled cell death pathways in PD [87]. The oxidative stress induced by ROS disrupts the delicate equilibrium between pro-apoptotic and anti-apoptotic signals that safeguard against cellular demise [88], thereby promoting apoptosis [89]. Furthermore, augmented levels of *Bax* have been documented across various PD models [90], thereby instigating the activation of the intrinsic apoptotic pathway [91]. In our current investigation, we observed a downregulation of *Bcl-2* alongside an upregulation of *Bax* in the rotenone-exposed group. Nevertheless, *T. aphylla* mitigated rotenone-induced apoptosis, demonstrated by the enhanced expression of *Bcl-2*, and diminished levels of *Bax*. The anti-apoptotic attributes of *T. aphylla* have undergone extensive scrutiny in previous *in vivo* experiments [72,92].

**Fig 19:**
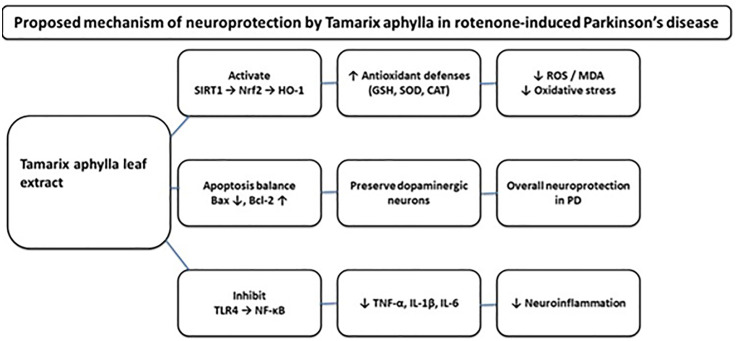
Graphical abstract of the proposed neuroprotective mechanism of *Tamarix aphylla* in a rotenone-induced Parkinson’s disease model.

TLR-4, a prominent member of the toll-like receptor family, stands out as a fundamental candidate pivotal in triggering the innate immune response [78]. Scientific reports underscore crucial involvement of TLR-4 in instigating neuroinflammation and neurodegeneration processes [79]. Elevated TLR-4 expression within the brain orchestrates NF-κB activation, which leads to releasing pro-inflammatory mediators [80,81]. Noteworthy investigations are shedding light on the potent capabilities of *T. aphylla* as an inhibitor of proinflammatory mediators and cytokines [82,83]. In this context, our study aimed to assess whether *T. aphylla* could modulate the TLR-4/NF-κB pathway. Our findings revealed that the administration of rotenone heightened TLR-4/NF-κB expression. Conversely, treatment with *T. aphylla* mitigated this activation, resulting in a curbed inflammatory response. These outcomes align with prior research affirming the role of rotenone in stimulating the TLR-4/NF-κB pathway [93]. The conclusions drawn from our study indicate that the protective effects of *T. aphylla* may be partially owing to its impact on the TLR-4/NF-κB pathway. A graphical summary (Fig 19) has been included to illustrate the multi-pathway neuroprotective mechanism proposed for *T. aphylla* in Parkinsonian neurodegeneration.

## Conclusion

Herein, we report, for the first time, that *T. aphylla* provides neuroprotection against rotenone-induced PD by modulating the expression of TH, α-Syn, and reducing neuroinflammation markers such as *TNF-α, IL-6*, and *IL-1β*. The protective effects are attributed to its antioxidant properties and modulation of the TLR-4/NF-κB signaling pathway, suggesting its potential as a novel therapeutic for PD. Compound **12** (tamarixin) displayed a prominent docking score of −7.98 kcal/mol and maintained a stable RMSD value of 0.7 nm, denoting a strong binding affinity with the Sirt-1 catalytic domain. These interactions were visualized, highlighting key residues such as GLN345, ILE347, PHE273, and ARG274. Molecular dynamics analyses over 150 ns for RMSD and potential energy demonstrated a stable ligand-protein complex, with consistent hydrogen bond formation further supporting the binding stability. This study reinforces the potential of compound **12** from *T. aphylla* as a promising neuroprotective agent in the treatment of PD.

## Supporting information

S1 Table Primer Sequences. (DOCX)

S2 Table Dereplicated Metabolites from the Crude Methanolic Extract of *T. aphylla* Leaves. (DOCX)

S3 Table List of Proteins Related to Parkinson’s Disease (PD). (DOCX)

S4 Table Results of Swiss Target Prediction for Compound 1. (DOCX)

S5 Table Results of Swiss Target Prediction for Compound 2. (DOCX)

S6 Table Results of Swiss Target Prediction for Compound 3. (DOCX)

S7 Table Results of Swiss Target Prediction for Compound 4. (DOCX)

S8 Table Results of Swiss Target Prediction for Compound 5. (DOCX)

S9 Table Results of Swiss Target Prediction for Compound 6. (DOCX)

S10 Table Results of Swiss Target Prediction for Compound 7. (DOCX)

S11 Table Results of Swiss Target Prediction for Compound 8. (DOCX)

S12 Table Results of Swiss Target Prediction for Compound 9. (DOCX)

S13 Table Results of Swiss Target Prediction for Compound 10. (DOCX)

S14 Table Results of Swiss Target Prediction for Compound 11. (DOCX)

S15 Table Results of Swiss Target Prediction for Compound 12. (DOCX)

S16 Table Results of Swiss Target Prediction for Compound 13. (DOCX)

S17 Table GO Enrichment Entry. (DOCX)

S18 Table KEGG Enrichment Entry. (DOCX)

S19 Table Binding Energies and RMSD of the 13 Compounds into the Active Pocket Site of the SIRT1 Catalytic Domain (PDB: 4i5i). (DOCX)

S1 Data Raw data. (XLSX)
