# Supplementary material for: Emphasizing the role of oxidative stress and Sirt-1/Nrf2 and TLR-4/NF-κB in Tamarix aphylla mediated neuroprotective potential in rotenone-induced Parkinson’s disease: In silico and in vivo study
Source: PLoS One. 2026 Jan 6;21(1):e0339010. doi: 10.1371/journal.pone.0339010 (PMC12774373; doi:10.1371/journal.pone.0339010)
Supplement: S1 Table — (DOCX) [file pone.0339010.s001.docx]

**Table S1. Primer Sequences.**

|  |  | Acc. no | Product size |  |
| --- | --- | --- | --- | --- |
| *IL-1β* | forward | NM_031512.2 | 281 | 5'-GTGATGAAAGACGGCACACC-3' |
|  | reverse |  |  | 5'-TCCTGGGGAAGGCATTAGGA -3' |
| *GAPDH* | forward | NM_017008.4 | 351 | 5'-CTC TCT GCT CCT CCC TGT TC-3' |
|  | reverse |  |  | 5'-CGA CAT ACT CAG CAC CAG CA-3' |
| *TNF-α* | forward | NM_012675.3 | 231 | 5'-CCT CTC TGC CAT CAA GAG CC-3' |
|  | reverse |  |  | 5'-GGC TGG GTA GAG AAC GGA TG-3' |
| *Bcl-2* | forward | NM_016993.2 | 382 | 5`-GGG CTA CGA GTG GGA TAC TG-`3 |
|  | reverse |  |  | 5`-GAC CCC ACC GAA CTC AAA GA-`3 |
| *Bax* | forward | NM_017059.2 | 292 | 5`-CAC GTC TGC GGG GAG TC-`3 |
|  | reverse |  |  | 5`-TGT TGT CCA GTT CAT CGC CA-`3 |
| *IL-6* | forward | NM_012589.2 | 299 | 5`-TCTGGTCTTCTGGAGTTCCGT-`3 |
|  | reverse |  |  | 5`-GGATGGTCTTGGTCCTTAGCC-`3 |
