# Supplementary material for: Emphasizing the role of oxidative stress and Sirt-1/Nrf2 and TLR-4/NF-κB in Tamarix aphylla mediated neuroprotective potential in rotenone-induced Parkinson’s disease: In silico and in vivo study
Source: PLoS One. 2026 Jan 6;21(1):e0339010. doi: 10.1371/journal.pone.0339010 (PMC12774373; doi:10.1371/journal.pone.0339010)
Supplement: S2 Table — (DOCX) [file pone.0339010.s002.docx]

**Table S2. Dereplicated Metabolites from the Crude Methanolic Extract of *T. aphylla* Leaves.**

| **No.** | **Compound**  **name** | **Concentration** | ***m/z*** | **Mol. formula** | **Source** |
| --- | --- | --- | --- | --- | --- |
| 1 | Isoferulaldehyde | 0.3175918 | 177.055 | C_10_H_10_O_3_ | *T. nilotica* |
| 2 | Troupin | 297922.28 | 235.060 | C_12_H_12_O_5_ | *T. troupii* |
| 3 | 3,7,8-Trihydroxy-2H,5H-pyrano[3,2-c][1]benzopyran-2,5-dione | 0.0435 | 261.007 | C_12_H_6_O_7_ |  |
| 4 | Isoferulic acid-3-sulphate | 10103.974 | 273.007 | C_10_H_10_O_7_S | *T. nilotica* |
| 5 | 3',4',7-Trihydroxy-5-methoxyflavone | 0.058 | 301.070 | C_16_H_12_O_6_ | *Tamarix* sp |
| 6 | 3,3',5,7-Tetrahydroxy-4'-methoxyflavone | 4.5732915 | 315.050 | C_16_H_12_O_7_ | *Tamarix* sp |
| 7 | Tameridone | 2.2632107 | 329.069 | C_17_H_14_O_7_ | *T. dioica* |
| 8 | 2,7-Di-methoxylellagic acid | 1045.882 | 329.032 | C_16_H_10_O_8_ | *T. gallica* |
| 9 | Aphyllin | 0.336298 | 355.103 | C_16_H_20_O_9_ | *T. aphylla* |
| 10 | Tamadone | 0.1334 | 359.076 | C_18_H_16_O_8_ | *T. dioica* |
| 11 | Rhamnocitrin 3-glucoside | 0.0609 | 463.123 | C_22_H_22_O_11_ | *Tamarix* sp |
| 12 | Tamarixin | 654.65631 | 477.104 | C_22_H_22_O_12_ | *Tamarix* sp |
| 13 | Ellagic acid 3,3′-dimethyl ether 4-*O*-*β*-D-glucopyranoside | 0.087 | 491.097 | C_22_H_20_O_13_ | *T. nilotica* |
