# Supplementary material for: Emphasizing the role of oxidative stress and Sirt-1/Nrf2 and TLR-4/NF-κB in Tamarix aphylla mediated neuroprotective potential in rotenone-induced Parkinson’s disease: In silico and in vivo study
Source: PLoS One. 2026 Jan 6;21(1):e0339010. doi: 10.1371/journal.pone.0339010 (PMC12774373; doi:10.1371/journal.pone.0339010)
Supplement: S3 Table — (DOCX) [file pone.0339010.s003.docx]

**Emphasizing the role of oxidative stress and Sirt-1/Nrf2 and TLR-4/NF-κB in *Tamarix aphylla* mediated neuroprotective potential in rotenone-induced Parkinson’s disease: *in silico* and *in vivo* study**

Dalia H. Abu-Baih ^1,2^, Abeer H. Elmaidomy ^3^, Hesham A. Abou-Zied ^4^, Nahla Abdelghany Hussien ^2^, Manar I. Rasekh ^2^, Entesar A. Saber ^5^, Sayed Fouad El-sheikh Ali ^6^, Mostafa E. Rateb ^7^, Omnia Magdy Hendawy ^8,9^, Faisal H. Altemani ^10^, Abdullah H. Altemani ^11^, Gerhard Bringmann ^12^, Usama Ramadan Abdelmohsen ^2,13^*, Omnia Hesham Abdelhafez ^13^*

^1^Department of Biochemistry and Molecular Biology, Faculty of Pharmacy, Deraya University, New Minia City, Minia 61111, Egypt; [dalia.hamdy@deraya.edu.eg](mailto:dalia.hamdy@deraya.edu.eg)

^2^Deraya Center for Scientific Research, Deraya University, New Minia City, Minia 61111, Egypt; [usama.ramadan@mu.edu.eg](mailto:usama.ramadan@mu.edu.eg), [nahla.abdalghany20@gmail.com](mailto:nahla.abdalghany20@gmail.com), manaribrahim912@gmail.com

^3^Department of Pharmacognosy, Faculty of Pharmacy, Beni-Suef University, Beni-Suef 62514, Egypt; abeer011150@pharm.bsu.edu.eg

^4^Department of Medicinal Chemistry, Faculty of Pharmacy, Deraya University, Minia 61111, Egypt; hisham.alaa@deraya.edu.eg

^5^Department of Medical Science, Histology and Cell Biology, Faculty of Pharmacy, Deraya University, New Minia City, Minia 61111, Egypt; entesar.ali@deraya.edu.eg

^6^Department of Anatomy, Faculty of Medicine, Minia University, Minia 61519, Egypt; sayed_fouad@mu.edu.eg

^7^Natural and Medical Sciences Research Center, University of Nizwa, P. O. Box 33, Birkat Al Mauz, Nizwa, Oman ; [m.rateb11@aberdeen.ac.uk](mailto:m.rateb11@aberdeen.ac.uk)

^8^Department of Pharmacology, College of Pharmacy, Jouf University, 72341 Skaka, Saudi Arabia; omhendawy@ju.edu.sa

^9^Department of Clinical Pharmacology, College of Medicine, Beni-Suef University, Beni-Suef, Egypt

^10^ Department of Medical Laboratory Technology, Faculty of Applied Medical Sciences, University of Tabuk, 71491 Tabuk, Saudi Arabia; [faltemani@ut.edu.sa](mailto:faltemani@ut.edu.sa)

^11^Department of Family and Community Medicine, Faculty of Medicine, University of Tabuk, 71491Tabuk, Saudi Arabia; atemani@ut.edu.sa

^12^Institute of Organic Chemistry, University of Würzburg, Am Hubland, 97074 Würzburg, Germany; gerhard.bringmann@uni-wuerzburg.de

^13^Department of Pharmacognosy, Faculty of Pharmacy, Deraya University, New Minia, 61111, Egypt; omnia.hesham@deraya.edu.eg

*****Correspondence: Usama Ramadan Abdelmohsen, Omnia Hesham Abdelhafez

**Running title**

***Tamarix aphylla* mediated neuroprotective potential in rotenone-induced Parkinson’s disease**

**ELISA**

Rat-specific ELISA kits for Sirt-1 (Elabscience, Texas, USA, Cat. no. E-EL-R1102), Nrf2 (Novus biologicals, Texas, USA, Cat. no. NBP3-08161), tyrosine hydroxylase and α-Syn were utilized for the assay of each respective target.

**Table S1. Primer Sequences.**

|  |  | Acc. no | Product size |  |
| --- | --- | --- | --- | --- |
| *IL-1β* | forward | NM_031512.2 | 281 | 5'-GTGATGAAAGACGGCACACC-3' |
|  | reverse |  |  | 5'-TCCTGGGGAAGGCATTAGGA -3' |
| *GAPDH* | forward | NM_017008.4 | 351 | 5'-CTC TCT GCT CCT CCC TGT TC-3' |
|  | reverse |  |  | 5'-CGA CAT ACT CAG CAC CAG CA-3' |
| *TNF-α* | forward | NM_012675.3 | 231 | 5'-CCT CTC TGC CAT CAA GAG CC-3' |
|  | reverse |  |  | 5'-GGC TGG GTA GAG AAC GGA TG-3' |
| *Bcl-2* | forward | NM_016993.2 | 382 | 5`-GGG CTA CGA GTG GGA TAC TG-`3 |
|  | reverse |  |  | 5`-GAC CCC ACC GAA CTC AAA GA-`3 |
| *Bax* | forward | NM_017059.2 | 292 | 5`-CAC GTC TGC GGG GAG TC-`3 |
|  | reverse |  |  | 5`-TGT TGT CCA GTT CAT CGC CA-`3 |
| *IL-6* | forward | NM_012589.2 | 299 | 5`-TCTGGTCTTCTGGAGTTCCGT-`3 |
|  | reverse |  |  | 5`-GGATGGTCTTGGTCCTTAGCC-`3 |

**Table S2. Dereplicated Metabolites from the Crude Methanolic Extract of *T. aphylla* Leaves.**

| **No.** | **Compound**  **name** | **Concentration** | ***m/z*** | **Mol. formula** | **Source** |
| --- | --- | --- | --- | --- | --- |
| 1 | Isoferulaldehyde | 0.3175918 | 177.055 | C_10_H_10_O_3_ | *T. nilotica* |
| 2 | Troupin | 297922.28 | 235.060 | C_12_H_12_O_5_ | *T. troupii* |
| 3 | 3,7,8-Trihydroxy-2H,5H-pyrano[3,2-c][1]benzopyran-2,5-dione | 0.0435 | 261.007 | C_12_H_6_O_7_ |  |
| 4 | Isoferulic acid-3-sulphate | 10103.974 | 273.007 | C_10_H_10_O_7_S | *T. nilotica* |
| 5 | 3',4',7-Trihydroxy-5-methoxyflavone | 0.058 | 301.070 | C_16_H_12_O_6_ | *Tamarix* sp |
| 6 | 3,3',5,7-Tetrahydroxy-4'-methoxyflavone | 4.5732915 | 315.050 | C_16_H_12_O_7_ | *Tamarix* sp |
| 7 | Tameridone | 2.2632107 | 329.069 | C_17_H_14_O_7_ | *T. dioica* |
| 8 | 2,7-Di-methoxylellagic acid | 1045.882 | 329.032 | C_16_H_10_O_8_ | *T. gallica* |
| 9 | Aphyllin | 0.336298 | 355.103 | C_16_H_20_O_9_ | *T. aphylla* |
| 10 | Tamadone | 0.1334 | 359.076 | C_18_H_16_O_8_ | *T. dioica* |
| 11 | Rhamnocitrin 3-glucoside | 0.0609 | 463.123 | C_22_H_22_O_11_ | *Tamarix* sp |
| 12 | Tamarixin | 654.65631 | 477.104 | C_22_H_22_O_12_ | *Tamarix* sp |
| 13 | Ellagic acid 3,3′-dimethyl ether 4-*O*-*β*-D-glucopyranoside | 0.087 | 491.097 | C_22_H_20_O_13_ | *T. nilotica* |

**Table S3. List of Proteins Related to Parkinson’s Disease (PD).**

| **Protein** | **Online library Source** |
| --- | --- |
| Tumor necrosis factor | **pharmGKB** |
| Interleukin 18 | **pharmGKB** |
| Absent in melanoma 2 | **pharmGKB** |
| Interleukin 4 | **pharmGKB** |
| Heme oxygenase 1 | **pharmGKB** |
| Interferon alpha/beta receptor 1 | **pharmGKB** |
| Interferon beta 1 | **pharmGKB** |
| Interferon alpha 2 | **pharmGKB** |
| NLR family pyrin domain containing 1 | **pharmGKB** |
| Interleukin 17A | **pharmGKB** |
| C-X-C Motif chemokine ligand 1 | **pharmGKB** |
| Toll-like receptor 4 | **pharmGKB** |
| NADPH oxidase 4 | **pharmGKB** |
| Interferon alpha 1 | **pharmGKB** |
| C-C Motif chemokine ligand 5 | **pharmGKB** |
| NLR family CARD domain containing 4 | **pharmGKB** |
| Interleukin 5 | **pharmGKB** |
| Interleukin 6 | **pharmGKB** |
| NLR family pyrin domain containing 3 | **NCBI** |
| Interleukin 10 | **NCBI** |
| Caspase 1 | **NCBI** |
| Caspase recruitment domain family member 8 | **NCBI** |
| Caspase 9 | **NCBI** |
| Interleukin 1 alpha | **NCBI** |
| Nitric oxide synthase 2 | **NCBI** |
| Colony stimulating factor 3 | **NCBI** |
| Colony stimulating factor 2 | **NCBI** |
| Sirtuin 1 | **NCBI** |
| Interferon gamma | **NCBI** |
| Interleukin 1 beta | **NCBI** |
| Peroxidasin | **NCBI** |
| Mediterranean fever gene | **NCBI** |
| Interleukin 37 | **NCBI** |
| C-C Motif chemokine ligand 4 | **NCBI** |
| Caspase 8 | **NCBI** |
| Interleukin 1 receptor antagonist | **NCBI** |
| Caspase 4 | **NCBI** |
| Interleukin 13 | **NCBI** |
| Actin beta | **NCBI** |
| Mannose receptor C-type 1 | **NCBI** |
| C-Reactive protein | **NCBI** |
| PYD and CARD domain containing | **NCBI** |
| Interleukin 33 | **NCBI** |
| C-X-C Motif chemokine ligand 8 | **NCBI** |
| Angiotensin-converting enzyme 2 | **NCBI** |
| Caspase 5 | **NCBI** |
| Cytochrome b-245 beta chain | **NCBI** |
| C-X-C Motif chemokine ligand 10 | **NCBI** |
| C-C Motif chemokine ligand 3 | **NCBI** |
| Interleukin 1 receptor accessory protein | **NCBI** |
| Toll-like receptor 9 | **NCBI** |
| Gasdermin D | **NCBI** |
| Interleukin 2 | **NCBI** |
| C-C Motif chemokine ligand 2 | **NCBI** |
| Glyceraldehyde-3-phosphate dehydrogenase | **NCBI** |
| Interleukin 15 (IL-15) | **NCBI** |
| Interleukin 18 binding protein (IL-18BP) | **NCBI** |
| Chemokine (C-X-C motif) ligand 12 (CXCL12) | **NCBI** |
| Matrix metalloproteinase 9 (MMP-9) | **NCBI** |
| High mobility group box 1 (HMGB1) | **NCBI** |
| Interleukin 22 (IL-22) | **NCBI** |
| Fractalkine (CX3CL1) | **NCBI** |
| Neuropeptide Y (NPY) | **NCBI** |
| Triggering receptor expressed on myeloid cells 2 (TREM2) | **NCBI** |
| Programmed death-ligand 1 (PD-L1) | **NCBI** |

**PharmGKB (Pharmacogenomics Knowledgebasewww.pharmgkb.org)**

**NCBI (National Center for Biotechnology Information www.ncbi.nlm.nih.gov)**

**Table S4. Results of Swiss Target Prediction for Compound 1.**

| **No.** | **Name** |
| --- | --- |
| 1 | 11-Beta-hydroxysteroid dehydrogenase 1 |
| 2 | Absent in melanoma 2 |
| 3 | ADAM17 |
| 4 | Aldose reductase |
| 5 | Alkaline phosphatase placental-like |
| 6 | Alkaline phosphatase, tissue-nonspecific isozyme |
| 7 | Angiotensin-converting enzyme (by homology) |
| 8 | Apoptosis regulator Bcl-2 |
| 9 | Arachidonate 5-lipoxygenase |
| 10 | Aryl hydrocarbon receptor |
| 11 | Arylamine N-acetyltransferase 1 |
| 12 | Beta amyloid A4 protein |
| 13 | Beta-secretase 1 |
| 14 | Calcium-activated potassium channel subunit alpha-1 |
| 15 | Carbonic anhydrase I |
| 16 | Carbonic anhydrase II |
| 17 | Carbonic anhydrase IX |
| 18 | Carbonic anhydrase VA |
| 19 | Carbonic anhydrase VB |
| 20 | Carbonic anhydrase VI |
| 21 | Carbonic anhydrase VII |
| 22 | Carbonic anhydrase XII |
| 23 | Carbonic anhydrase XIII |
| 24 | Carbonic anhydrase XIV |
| 25 | Casein kinase I alpha |
| 26 | Casein kinase I delta |
| 27 | CDGSH iron-sulfur domain-containing protein 1 |
| 28 | c-Jun N-terminal kinase 1 |
| 29 | Coagulation factor VII/tissue factor |
| 30 | Cyclin-dependent kinase 1/cyclin B |
| 31 | Cyclin-dependent kinase 1/cyclin B1 |
| 32 | Cyclin-dependent kinase 2/cyclin E |
| 33 | Cyclin-dependent kinase 4/cyclin D1 |
| 34 | Cyclin-dependent kinase 5/CDK5 activator 1 |
| 35 | Cyclooxygenase-1 |
| 36 | Cyclooxygenase-2 |
| 37 | Cytochrome P450 19A1 |
| 38 | Cytochrome P450 1A1 |
| 39 | Cytochrome P450 1A2 |
| 40 | Cytochrome P450 1B1 |
| 41 | Cytochrome P450 3A4 |
| 42 | D-Amino-acid oxidase |
| 43 | DNA topoisomerase II alpha |
| 44 | Epidermal growth factor receptor erbB1 |
| 45 | Estrogen receptor alpha |
| 46 | Estrogen receptor beta |
| 47 | Fructose-1,6-bisphosphatase |
| 48 | Glyoxalase I |
| 49 | G-protein coupled receptor kinase 2 |
| 50 | Heme oxygenase 1 |
| 51 | Histone acetyltransferase p300 |
| 52 | Histone deacetylase 4 |
| 53 | Histone deacetylase 5 |
| 54 | Histone deacetylase 7 |
| 55 | HMG-CoA reductase |
| 56 | Inhibitor of NF-kappa-B kinase (IKK) |
| 57 | Interleukin 18 |
| 58 | Interleukin 4 |
| 59 | Leukocyte common antigen |
| 60 | Lysine-specific demethylase 4A |
| 61 | Lysine-specific demethylase 4B |
| 62 | Lysine-specific demethylase 5B |
| 63 | Lysine-specific demethylase 5C |
| 64 | Matrix metalloproteinase 1 |
| 65 | Matrix metalloproteinase 13 |
| 66 | Matrix metalloproteinase 2 |
| 67 | Matrix metalloproteinase 3 |
| 68 | Matrix metalloproteinase 8 |
| 69 | Matrix metalloproteinase 9 |
| 70 | Metabotropic glutamate receptor 4 |
| 71 | Metabotropic glutamate receptor 5 |
| 72 | Monoamine oxidase A |
| 73 | Monoamine oxidase B |
| 74 | Monocarboxylate transporter 1 (by homology) |
| 75 | Myoglobin |
| 76 | Myosin light chain kinase, smooth muscle |
| 77 | Nuclear factor erythroid 2-related factor 2 |
| 78 | Nuclear factor NF-kappa-B p65 subunit |
| 79 | Palmitoleoyl-protein carboxylesterase NOTUM |
| 80 | P-Glycoprotein 1 |
| 81 | Phosphodiesterase 5A |
| 82 | Phospholipase A-2-activating protein |
| 83 | Plectin |
| 84 | Poly [ADP-ribose] polymerase-1 |
| 85 | Protein kinase C mu |
| 86 | Protein-tyrosine phosphatase 1B |
| 87 | Quinone reductase 2 |
| 88 | Serine/threonine-protein kinase D2 |
| 89 | Serine/threonine-protein kinase Nek1 |
| 90 | Serine/threonine-protein kinase PIM1 |
| 91 | Serine/threonine-protein kinase PIM2 |
| 92 | Serine/threonine-protein kinase PIM3 |
| 93 | Signal transducer and activator of transcription 3 |
| 94 | Transthyretin |
| 95 | Tubulin beta-1 chain |
| 96 | Tubulin beta-3 chain |
| 97 | Tumor Necrosis Factor |
| 98 | Xanthine dehydrogenase |

**Table S5. Results of Swiss Target Prediction for Compound 2.**

| **No.** | **Name** |
| --- | --- |
| 1 | Acetylcholinesterase |
| 2 | Adenosine A2b receptor |
| 3 | Adrenergic receptor alpha-2 |
| 4 | Aldehyde dehydrogenase |
| 5 | Alkaline phosphatase, tissue-nonspecific isozyme |
| 6 | Alpha-2a adrenergic receptor |
| 7 | Alpha-2b adrenergic receptor |
| 8 | Amine oxidase, copper containing |
| 9 | Androgen receptor |
| 10 | Arylamine N-acetyltransferase 1 |
| 11 | Beta-secretase 1 |
| 12 | Calcium-activated potassium channel subunit alpha-1 |
| 13 | Carbonic anhydrase I |
| 14 | Carbonic anhydrase II |
| 15 | Carbonic anhydrase III |
| 16 | Carbonic anhydrase IX |
| 17 | Carbonic anhydrase VI |
| 18 | Carbonic anhydrase VII |
| 19 | Carbonic anhydrase XII |
| 20 | Carbonic anhydrase XIII |
| 21 | Carbonic anhydrase XIV |
| 22 | Carbonyl reductase [NADPH] 1 |
| 23 | Casein kinase I alpha |
| 24 | Casein kinase I delta |
| 25 | Caspase-6 |
| 26 | Cathepsin (V and K) |
| 27 | CDC7/DBF4 (cell division cycle 7-related protein kinase/activator of S phase kinase) |
| 28 | CDGSH iron-sulfur domain-containing protein 1 |
| 29 | CDK9/cyclin T1 |
| 30 | c-Jun N-terminal kinase 1 |
| 31 | Coagulation factor XII |
| 32 | Cyclin-dependent kinase 1/cyclin B |
| 33 | Cyclin-dependent kinase 2/cyclin A |
| 34 | Cytochrome P450 1A2 |
| 35 | Dual specificity tyrosine-phosphorylation-regulated kinase 1B |
| 36 | Dual-specificity tyrosine-phosphorylation regulated kinase 1A (by homology) |
| 37 | Egl nine homolog 1 |
| 38 | Epidermal growth factor receptor erbB1 |
| 39 | Estradiol 17-beta-dehydrogenase 3 |
| 40 | Estrogen receptor beta |
| 41 | Fibroblast growth factor receptor 1 |
| 42 | Focal adhesion kinase 1 |
| 43 | G-Protein-coupled receptor kinase 6 |
| 44 | GABA A receptor alpha-3/beta-2/gamma-2 |
| 45 | GABA-A receptor; alpha-1/beta-2/gamma-2 |
| 46 | Gamma-amino-N-butyrate transaminase (by homology) |
| 47 | Glutathione reductase |
| 48 | Glutathione S-transferase A1 |
| 49 | Glycogen synthase kinase-3 alpha |
| 50 | Heat shock 70 kDa protein 1 |
| 51 | Hepatocyte growth factor receptor |
| 52 | Histone chaperone ASF1A |
| 53 | Histone deacetylase 2 |
| 54 | Histone deacetylase 4 |
| 55 | Histone deacetylase 5 |
| 56 | Histone deacetylase 7 |
| 57 | Histone deacetylase 8 |
| 58 | HMG-CoA reductase |
| 59 | Interferon alpha 2 |
| 60 | Interferon alpha/beta receptor 1 |
| 61 | Interferon beta 1 |
| 62 | Interleukin 17A |
| 63 | Interleukin-8 receptor A |
| 64 | Kinesin-1 heavy chain/tyrosine-protein kinase receptor RET |
| 65 | LDL-associated phospholipase A2 |
| 66 | Leukocyte elastase |
| 67 | L-Lactate dehydrogenase A chain |
| 68 | Lysine-specific histone demethylase 1 |
| 69 | Macrophage scavenger receptor types I and II |
| 70 | Mitogen-activated protein kinase kinase kinase 8 |
| 71 | Monoamine oxidase A |
| 72 | Monoamine oxidase B |
| 73 | Muscarinic acetylcholine receptor M1 (by homology) |
| 74 | Myoglobin |
| 75 | Nischarin |
| 76 | NLR family pyrin domain containing 1 |
| 77 | N-Lysine methyltransferase SETD8 |
| 78 | NUAK family SNF1-like kinase 1 |
| 79 | Phosphodiesterase 5A |
| 80 | PI3-Kinase p110-gamma subunit |
| 81 | Plectin |
| 82 | Poly [ADP-ribose] polymerase-1 |
| 83 | Progesterone receptor |
| 84 | Serine/threonine-protein kinase Chk1 |
| 85 | Serine/threonine-protein kinase PIM2 |
| 86 | Serine/threonine-protein kinase/endoribonuclease IRE1 |
| 87 | Serine-protein kinase ATR |
| 88 | Serotonin 2b (5-HT2b) receptor |
| 89 | Thrombin |
| 90 | Thymidylate synthase |
| 91 | Trace amine-associated receptor 1(by homology) |
| 92 | Tyrosine-protein kinase JAK1 |
| 93 | Tyrosine-protein kinase JAK2 |
| 94 | Tyrosine-protein kinase JAK3 |
| 95 | Tyrosine-protein kinase TYK2 |
| 96 | Vascular endothelial growth factor receptor 1 |
| 97 | Vascular endothelial growth factor receptor 2 |
| 98 | Voltage-gated potassium channel subunit Kv1.3 |

**Table S6. Results of Swiss Target Prediction for Compound 3.**

| **No.** | **Name** |
| --- | --- |
| 1 | 14-3-3 Protein gamma |
| 2 | Acetylcholinesterase |
| 3 | Aldose reductase |
| 4 | ALK tyrosine kinase receptor |
| 5 | Alkaline phosphatase, tissue-nonspecific isozyme |
| 6 | Alpha-synuclein |
| 7 | Arachidonate 5-lipoxygenase |
| 8 | Beta amyloid A4 protein |
| 9 | Beta-secretase 1 |
| 10 | Carbonic anhydrase I |
| 11 | Carbonic anhydrase II |
| 12 | Carbonic anhydrase IV |
| 13 | Carbonic anhydrase IX |
| 14 | Carbonic anhydrase VA |
| 15 | Carbonic anhydrase VI |
| 16 | Carbonic anhydrase VII |
| 17 | Carbonic anhydrase XII |
| 18 | Carbonic anhydrase XIII |
| 19 | Carbonic anhydrase XIV |
| 20 | Carbonyl reductase [NADPH] 1 |
| 21 | Casein kinase II alpha |
| 22 | Cyclin-dependent kinase 2/cyclin A |
| 23 | Cyclin-dependent kinase 2/cyclin E1 |
| 24 | Cyclin-dependent kinase 4/cyclin D1 |
| 25 | Cyclin-dependent kinase 5/CDK5 activator 1 |
| 26 | Cyclooxygenase-1 |
| 27 | Cyclooxygenase-2 |
| 28 | Cytochrome P450 1A2 |
| 29 | D-Amino-acid oxidase |
| 30 | Dihydrofolate reductase |
| 31 | DNA topoisomerase I |
| 32 | Ephrin receptor |
| 33 | Epidermal growth factor receptor erbB1 |
| 34 | Estradiol 17-beta-dehydrogenase 1 |
| 35 | Estradiol 17-beta-dehydrogenase 3 |
| 36 | Estrogen receptor alpha |
| 37 | Estrogen receptor beta |
| 38 | Fibroblast growth factor receptor 1 |
| 39 | Focal adhesion kinase 1 |
| 40 | Glutamate receptor ionotropic, AMPA 1 |
| 41 | Glutamate receptor ionotropic, AMPA 2 |
| 42 | Glutamate receptor ionotropic, AMPA 3 |
| 43 | Glutamate receptor ionotropic, AMPA 4 |
| 44 | Glutathione reductase |
| 45 | Glycogen synthase kinase-3 beta |
| 46 | G-protein coupled receptor 35 |
| 47 | Heat shock 70 kDa protein 1 |
| 48 | Hepatocyte growth factor receptor |
| 49 | Histone deacetylase 6 |
| 50 | Insulin receptor |
| 51 | Insulin-like growth factor binding protein 3 |
| 52 | Insulin-like growth factor I receptor |
| 53 | Interferon alpha 2 |
| 54 | Interferon alpha/beta receptor 1 |
| 55 | Interferon beta 1 |
| 56 | Interleukin 17A |
| 57 | Mannose-6-phosphate isomerase |
| 58 | Matrix metalloproteinase 2 |
| 59 | Matrix metalloproteinase 9 |
| 60 | Microtubule-associated protein tau |
| 61 | Mitogen-activated protein kinase kinase kinase 8 |
| 62 | Monoamine oxidase A |
| 63 | NLR family pyrin domain containing 1 |
| 64 | NUAK family SNF1-like kinase 1 |
| 65 | Nuclear factor NF-kappa-B p105 subunit |
| 66 | Phospholipase A-2-activating protein |
| 67 | PI3-Kinase p110-gamma subunit |
| 68 | Plasminogen activator inhibitor-1 |
| 69 | Platelet-derived growth factor receptor beta |
| 70 | Potassium-transporting ATPase |
| 71 | Protein-tyrosine phosphatase 2C |
| 72 | Receptor protein-tyrosine kinase erbB-2 |
| 73 | Serine/threonine-protein kinase AKT |
| 74 | Serine/threonine-protein kinase aurora-A |
| 75 | Serine/threonine-protein kinase aurora-B |
| 76 | Serine/threonine-protein kinase B-raf |
| 77 | Serine/threonine-protein kinase Chk1 |
| 78 | Serine/threonine-protein kinase PIM1 |
| 79 | Serine/threonine-protein kinase PLK1 |
| 80 | Serine/threonine-protein kinase PLK4 |
| 81 | Squalene monooxygenase (by homology) |
| 82 | Stem cell growth factor receptor |
| 83 | Thrombin |
| 84 | Thymidine phosphorylase |
| 85 | Thymidylate synthase |
| 86 | Transmembrane domain-containing protein TMIGD3 |
| 87 | Troponin, cardiac muscle |
| 88 | Tyrosine-protein kinase FGR (by homology) |
| 89 | Tyrosine-protein kinase Lyn (by homology) |
| 90 | Tyrosine-protein kinase receptor UFO |
| 91 | Tyrosine-protein kinase SRC |
| 92 | Tyrosine-protein kinase TIE-2 |
| 93 | Tyrosyl-DNA phosphodiesterase 1 |
| 94 | Uridine phosphorylase 1 (by homology) |
| 95 | Vascular endothelial growth factor receptor 2 |
| 96 | Vascular endothelial growth factor receptor 3 |
| 97 | Voltage-gated potassium channel subunit Kv1.3 |
| 98 | Voltage-gated potassium channel subunit Kv1.5 |
| 99 | Xanthine dehydrogenase |

**Table S7. Results of Swiss Target Prediction for Compound 4.**

| **No.** | **Name** |
| --- | --- |
| 1 | 3-Phosphoinositide dependent protein kinase-1 |
| 2 | 6-Phosphofructo-2-kinase/fructose-2,6-bisphosphatase 3 |
| 3 | 6-Phosphofructo-2-kinase/fructose-2,6-bisphosphatase 4 |
| 4 | Acetylcholinesterase |
| 5 | ADAMTS5 |
| 6 | Adenosine A3 receptor |
| 7 | Adenosine kinase |
| 8 | Bifunctional protein NCOAT |
| 9 | Carbonic anhydrase I |
| 10 | Carbonic anhydrase II |
| 11 | Carbonic anhydrase IX |
| 12 | Carbonic anhydrase VA |
| 13 | Carbonic anhydrase VI |
| 14 | Carbonic anhydrase VII |
| 15 | Carbonic anhydrase XII |
| 16 | Carbonic anhydrase XIV |
| 17 | Casein kinase II alpha |
| 18 | Caspase-1 |
| 19 | Caspase-2 |
| 20 | Caspase-3 |
| 21 | Caspase-6 |
| 22 | Caspase-7 |
| 23 | Caspase-8 |
| 24 | C-C Chemokine receptor type 2 |
| 25 | C-C Motif chemokine ligand 5 |
| 26 | CDC7/DBF4 (cell division cycle 7-related protein kinase/activator of S phase kinase) |
| 27 | CDGSH iron-sulfur domain-containing protein 1 |
| 28 | CDK9/cyclin T1 |
| 29 | C-X-C Motif chemokine ligand 1 |
| 30 | Cyclin-dependent kinase 1 |
| 31 | Cyclin-dependent kinase 9 |
| 32 | Cytidine deaminase |
| 33 | D-amino-acid oxidase |
| 34 | Dihydroorotate dehydrogenase |
| 35 | DNA excision repair protein ERCC-5 |
| 36 | DNA (apurinic or apyrimidinic site) lyase |
| 37 | Dual specificity mitogen-activated protein kinase kinase 1 |
| 38 | Dual specificity protein kinase CLK2 (by homology) |
| 39 | Dual specificity protein kinase CLK4 (by homology) |
| 40 | Dual specificity protein kinase CLK1 (by homology) |
| 41 | Dual-specificity tyrosine-phosphorylation regulated kinase 3 |
| 42 | Endothelin-converting enzyme 1 |
| 43 | Farnesyl diphosphate synthase |
| 44 | Fatty acid binding protein adipocyte |
| 45 | Fatty acid binding protein intestinal |
| 46 | Fatty acid binding protein muscle |
| 47 | Flap endonuclease 1 |
| 48 | Glutamate carboxypeptidase II |
| 49 | Glutamate receptor ionotropic kainate 1 |
| 50 | Glutathione S-transferase Mu 2 |
| 51 | Glutathione S-transferase Pi |
| 52 | G-protein coupled receptor 35 |
| 53 | G-protein coupled receptor 55 |
| 54 | Hydroxyacid oxidase 1 |
| 55 | Hydroxyacid oxidase 2 (by homology) |
| 56 | Hypoxia-inducible factor prolyl 4-hydroxylase |
| 57 | Intercellular adhesion molecule (ICAM-1), integrin alpha-L/beta-2 |
| 58 | Interferon alpha 1 |
| 59 | Kynurenine 3-monooxygenase |
| 60 | Liver glycogen phosphorylase |
| 61 | Lysine-specific demethylase 4A |
| 62 | Lysine-specific demethylase 4B |
| 63 | Lysine-specific demethylase 4C |
| 64 | Lysine-specific demethylase 4D |
| 65 | Lysine-specific demethylase 4D-like |
| 66 | Lysine-specific demethylase 5A |
| 67 | Lysine-specific demethylase 5B |
| 68 | Lysine-specific demethylase 5C |
| 69 | Lysine-specific demethylase 6B |
| 70 | Macrophage migration inhibitory factor |
| 71 | Matrix metalloproteinase 12 |
| 72 | Methionine aminopeptidase 2 |
| 73 | Monocarboxylate transporter 4 |
| 74 | Muscarinic acetylcholine receptor M1 |
| 75 | Muscle glycogen phosphorylase |
| 76 | NADPH oxidase 4 |
| 77 | Neuronal acetylcholine receptor; alpha4/beta2 |
| 78 | Neurotensin receptor 3 |
| 79 | Peptidyl-prolyl cis-trans isomerase NIMA-interacting 1 |
| 80 | Peroxisome proliferator-activated receptor gamma |
| 81 | Phosphodiesterase 10A |
| 82 | Phosphodiesterase 4B |
| 83 | Poly [ADP-ribose] polymerase 10 |
| 84 | Poly [ADP-ribose] polymerase 15 |
| 85 | Poly [ADP-ribose] polymerase-1 |
| 86 | Prostanoid EP1 receptor |
| 87 | Protein tyrosine kinase 2 beta |
| 88 | Renin |
| 89 | Ribonuclease H1 |
| 90 | Serine/threonine-protein kinase PIM1 |
| 91 | Solute carrier family 22 member 12 |
| 92 | Solute carrier organic anion transporter family member 1B1 |
| 93 | Squalene synthetase (by homology) |
| 94 | T1R1/T1R3_UNCURATED |
| 95 | Thrombin |
| 96 | Thromboxane-A synthase |
| 97 | Thymidine phosphorylase (by homology) |
| 98 | Toll-like receptor 4 |
| 99 | Voltage-gated potassium channel subunit Kv1.3 |

**Table S8. Results of Swiss Target Prediction for Compound 5.**

| **No.** | **Name** |
| --- | --- |
| 1 | Acetylcholinesterase |
| 2 | Adenosine A1 receptor (by homology) |
| 3 | Adenosine A2a receptor (by homology) |
| 4 | Aldo-keto reductase family 1 member B10 |
| 5 | Aldo-keto reductase family 1 member C1 (by homology) |
| 6 | Aldo-keto reductase family 1 member C2 (by homology) |
| 7 | Aldo-keto reductase family 1 member C4 (by homology) |
| 8 | Aldo-keto-reductase family 1 member C3 (by homology) |
| 9 | Aldose reductase (by homology) |
| 10 | ALK tyrosine kinase receptor |
| 11 | AMY1C |
| 12 | Arachidonate 12-lipoxygenase |
| 13 | Arachidonate 15-lipoxygenase |
| 14 | Arachidonate 5-lipoxygenase |
| 15 | Arginase-1 (by homology) |
| 16 | ATP-binding cassette sub-family G member 2 |
| 17 | Beta amyloid A4 protein |
| 18 | Beta-secretase 1 |
| 19 | CaM kinase II beta |
| 20 | Carbonic anhydrase I |
| 21 | Carbonic anhydrase II |
| 22 | Carbonic anhydrase III |
| 23 | Carbonic anhydrase IV |
| 24 | Carbonic anhydrase IX |
| 25 | Carbonic anhydrase VA |
| 26 | Carbonic anhydrase VI |
| 27 | Carbonic anhydrase VII |
| 28 | Carbonic anhydrase XII |
| 29 | Carbonic anhydrase XIV |
| 30 | Casein kinase II alpha |
| 31 | Cyclin-dependent kinase 1 |
| 32 | Cyclin-dependent kinase 1/cyclin B |
| 33 | Cyclin-dependent kinase 5/CDK5 activator 1 |
| 34 | Cyclin-dependent kinase 6 |
| 35 | Cyclooxygenase-2 |
| 36 | Cystic fibrosis transmembrane conductance regulator |
| 37 | Cytochrome P450 19A1 |
| 38 | Cytochrome P450 1B1 |
| 39 | Death-associated protein kinase 1 |
| 40 | Delta opioid receptor |
| 41 | DNA topoisomerase I (by homology) |
| 42 | Dopamine D4 receptor |
| 43 | Epidermal growth factor receptor erbB1 |
| 44 | Estradiol 17-beta-dehydrogenase 1 |
| 45 | Estradiol 17-beta-dehydrogenase 2 |
| 46 | Estrogen receptor alpha |
| 47 | Estrogen receptor beta |
| 48 | Focal adhesion kinase 1 |
| 49 | G-Protein-coupled receptor kinase 6 |
| 50 | Glycogen synthase kinase-3 beta |
| 51 | Glyoxalase I |
| 52 | G-protein coupled receptor 35 |
| 53 | Hepatocyte growth factor receptor |
| 54 | Induced myeloid leukemia cell differentiation protein Mcl-1 |
| 55 | Insulin-like growth factor I receptor |
| 56 | Interleukin 10 |
| 57 | Interleukin 5 |
| 58 | Interleukin 6 |
| 59 | Interleukin-8 receptor A |
| 60 | Liver glycogen phosphorylase |
| 61 | Lymphocyte differentiation antigen CD38 |
| 62 | Matrix metalloproteinase 12 |
| 63 | Matrix metalloproteinase 13 |
| 64 | Matrix metalloproteinase 2 |
| 65 | Matrix metalloproteinase 3 |
| 66 | Matrix metalloproteinase 9 |
| 67 | Monoamine oxidase A |
| 68 | Multidrug resistance-associated protein 1 |
| 69 | Myeloperoxidase |
| 70 | NADPH oxidase 4 |
| 71 | NLR family CARD domain containing 4 |
| 72 | NLR family pyrin domain containing 3 |
| 73 | NUAK family SNF1-like kinase 1 |
| 74 | P-Glycoprotein 1 |
| 75 | Phospholipase A2 group 1B |
| 76 | Phospholipase A2 group IIA |
| 77 | PI3-Kinase p85-alpha subunit |
| 78 | Plasminogen |
| 79 | Poly [ADP-ribose] polymerase-1 |
| 80 | Protein kinase N1 |
| 81 | Receptor-type tyrosine-protein phosphatase S |
| 82 | Serine/threonine-protein kinase AKT |
| 83 | Serine/threonine-protein kinase aurora-B |
| 84 | Serine/threonine-protein kinase NEK2 |
| 85 | Serine/threonine-protein kinase NEK6 |
| 86 | Serine/threonine-protein kinase PIM1 |
| 87 | Serine/threonine-protein kinase PLK1 |
| 88 | Tankyrase-1 |
| 89 | Tankyrase-2 |
| 90 | Telomerase reverse transcriptase |
| 91 | Thrombin |
| 92 | Transthyretin |
| 93 | Tyrosine-protein kinase receptor FLT3 |
| 94 | Tyrosine-protein kinase receptor UFO |
| 95 | Tyrosine-protein kinase SRC |
| 96 | Tyrosine-protein kinase SYK |
| 97 | Vascular endothelial growth factor receptor 2 |
| 98 | Vasopressin V2 receptor |
| 99 | Xanthine dehydrogenase |

**Table S9. Results of Swiss Target Prediction for Compound 6.**

| **No.** | **Name** |
| --- | --- |
| 1 | Acetylcholinesterase |
| 2 | Adenosine A1 receptor (by homology) |
| 3 | Adenosine A2a receptor (by homology) |
| 4 | Aldehyde reductase (by homology) |
| 5 | Aldo-keto reductase family 1 member C1 (by homology) |
| 6 | Aldo-keto reductase family 1 member C2 (by homology) |
| 7 | Aldo-keto reductase family 1 member C4 (by homology) |
| 8 | Aldo-keto-reductase family 1 member C3 (by homology) |
| 9 | Aldose reductase |
| 10 | ALK tyrosine kinase receptor |
| 11 | Arachidonate 12-lipoxygenase |
| 12 | Arachidonate 15-lipoxygenase |
| 13 | Arachidonate 5-lipoxygenase |
| 14 | Arginase-1 (by homology) |
| 15 | Aryl hydrocarbon receptor |
| 16 | ATP-binding cassette sub-family G member 2 |
| 17 | Beta amyloid A4 protein |
| 18 | Beta-secretase 1 |
| 19 | CaM kinase II beta |
| 20 | Carbonic anhydrase I |
| 21 | Carbonic anhydrase II |
| 22 | Carbonic anhydrase III |
| 23 | Carbonic anhydrase IV |
| 24 | Carbonic anhydrase IX |
| 25 | Carbonic anhydrase VA |
| 26 | Carbonic anhydrase VI |
| 27 | Carbonic anhydrase VII |
| 28 | Carbonic anhydrase XII |
| 29 | Carbonic anhydrase XIII (by homology) |
| 30 | Carbonic anhydrase XIV |
| 31 | Casein kinase II alpha |
| 32 | Caspase 1 |
| 33 | Caspase 9 |
| 34 | Caspase recruitment domain family member 8 |
| 35 | Cyclin-dependent kinase 1 |
| 36 | Cyclin-dependent kinase 1/cyclin B |
| 37 | Cyclin-dependent kinase 2 |
| 38 | Cyclin-dependent kinase 5/CDK5 activator 1 |
| 39 | Cyclin-dependent kinase 6 |
| 40 | Cytochrome P450 19A1 |
| 41 | Cytochrome P450 1B1 |
| 42 | Death-associated protein kinase 1 |
| 43 | DNA topoisomerase II alpha |
| 44 | DNA-(apurinic or apyrimidinic site) lyase |
| 45 | DNA-3-methyladenine glycosylase |
| 46 | Dopamine D4 receptor |
| 47 | Epidermal growth factor receptor erbB1 |
| 48 | Estradiol 17-beta-dehydrogenase 1 |
| 49 | Estradiol 17-beta-dehydrogenase 2 |
| 50 | Estrogen receptor beta |
| 51 | Estrogen-related receptor alpha |
| 52 | Focal adhesion kinase 1 |
| 53 | Glycogen synthase kinase-3 beta |
| 54 | Glyoxalase I |
| 55 | G-protein coupled receptor 35 |
| 56 | Hepatocyte growth factor receptor |
| 57 | Induced myeloid leukemia cell differentiation protein Mcl-1 |
| 58 | Insulin receptor |
| 59 | Insulin-like growth factor I receptor |
| 60 | Interleukin 1 alpha |
| 61 | Interleukin-8 receptor A |
| 62 | Liver glycogen phosphorylase |
| 63 | Lysine-specific demethylase 4D-like |
| 64 | Matrix metalloproteinase 13 |
| 65 | Matrix metalloproteinase 2 |
| 66 | Matrix metalloproteinase 3 |
| 67 | Matrix metalloproteinase 9 |
| 68 | Microtubule-associated protein tau |
| 69 | Monoamine oxidase A |
| 70 | Multidrug resistance-associated protein 1 |
| 71 | Myeloperoxidase |
| 72 | Myosin light chain kinase, smooth muscle |
| 73 | NADPH oxidase 4 |
| 74 | Nitric oxide synthase 2 |
| 75 | NUAK family SNF1-like kinase 1 |
| 76 | P-Glycoprotein 1 |
| 77 | Phospholipase A2 group 1B |
| 78 | PI3-Kinase p110-gamma subunit |
| 79 | PI3-Kinase p85-alpha subunit |
| 80 | Plasminogen |
| 81 | Protein kinase N1 |
| 82 | Receptor-type tyrosine-protein phosphatase S |
| 83 | Serine/threonine-protein kinase AKT |
| 84 | Serine/threonine-protein kinase aurora-B |
| 85 | Serine/threonine-protein kinase NEK2 |
| 86 | Serine/threonine-protein kinase NEK6 |
| 87 | Serine/threonine-protein kinase PIM1 |
| 88 | Serine/threonine-protein kinase PLK1 |
| 89 | Solute carrier family 22 member 12 |
| 90 | Telomerase reverse transcriptase |
| 91 | Thrombin |
| 92 | Tyrosinase |
| 93 | Tyrosine-protein kinase receptor FLT3 |
| 94 | Tyrosine-protein kinase receptor UFO |
| 95 | Tyrosine-protein kinase SRC |
| 96 | Tyrosine-protein kinase SYK |
| 97 | Vascular endothelial growth factor receptor 2 |
| 98 | Vasopressin V2 receptor |
| 99 | Xanthine dehydrogenase |

**Table S10. Results of Swiss Target Prediction for Compound 7.**

| **No.** | **Name** |
| --- | --- |
| 1 | 6-Phosphofructo-2-kinase/fructose-2,6-bisphosphatase 3 |
| 2 | Acetylcholinesterase |
| 3 | Adenosine A1 receptor (by homology) |
| 4 | Adenosine A2a receptor (by homology) |
| 5 | Aldehyde reductase (by homology) |
| 6 | Aldo-keto reductase family 1 member C1 (by homology) |
| 7 | Aldo-keto reductase family 1 member C2 (by homology) |
| 8 | Aldo-keto reductase family 1 member C4 (by homology) |
| 9 | Aldo-keto-reductase family 1 member C3 (by homology) |
| 10 | Aldose reductase (by homology) |
| 11 | ALK tyrosine kinase receptor |
| 12 | Arachidonate 12-lipoxygenase |
| 13 | Arachidonate 15-lipoxygenase |
| 14 | Arachidonate 5-lipoxygenase |
| 15 | Arginase-1 (by homology) |
| 16 | ATP-binding cassette sub-family G member 2 |
| 17 | Beta amyloid A4 protein |
| 18 | Beta-secretase 1 |
| 19 | CaM kinase II beta |
| 20 | Carbonic anhydrase I |
| 21 | Carbonic anhydrase II |
| 22 | Carbonic anhydrase III |
| 23 | Carbonic anhydrase IV |
| 24 | Carbonic anhydrase IX |
| 25 | Carbonic anhydrase VA |
| 26 | Carbonic anhydrase VI |
| 27 | Carbonic anhydrase VII |
| 28 | Carbonic anhydrase XII |
| 29 | Carbonic anhydrase XIII (by homology) |
| 30 | Carbonic anhydrase XIV |
| 31 | Casein kinase II alpha |
| 32 | Colony stimulating factor 2 |
| 33 | Colony stimulating factor 3 |
| 34 | Cyclin-dependent kinase 1 |
| 35 | Cyclin-dependent kinase 1/cyclin B |
| 36 | Cyclin-dependent kinase 5/CDK5 activator 1 |
| 37 | Cyclooxygenase-2 (by homology) |
| 38 | Cytochrome P450 19A1 |
| 39 | Cytochrome P450 1A1 |
| 40 | Cytochrome P450 1A2 |
| 41 | Cytochrome P450 1B1 |
| 42 | Death-associated protein kinase 1 |
| 43 | Delta opioid receptor |
| 44 | Dopamine D4 receptor |
| 45 | Endoplasmin |
| 46 | Epidermal growth factor receptor erbB1 |
| 47 | Estradiol 17-beta-dehydrogenase 1 |
| 48 | Estradiol 17-beta-dehydrogenase 2 |
| 49 | Estrogen receptor alpha |
| 50 | Estrogen receptor beta |
| 51 | Focal adhesion kinase 1 |
| 52 | Glycogen synthase kinase-3 beta |
| 53 | Glyoxalase I |
| 54 | G-Protein coupled receptor 35 |
| 55 | Hepatocyte growth factor receptor |
| 56 | Induced myeloid leukemia cell differentiation protein Mcl-1 |
| 57 | Insulin-like growth factor I receptor |
| 58 | Interferon gamma |
| 59 | Interleukin 1 beta |
| 60 | Interleukin-8 receptor A |
| 61 | Liver glycogen phosphorylase |
| 62 | Matrix metalloproteinase 13 |
| 63 | Matrix metalloproteinase 2 |
| 64 | Matrix metalloproteinase 3 |
| 65 | Matrix metalloproteinase 9 |
| 66 | Monoamine oxidase A |
| 67 | Mu opioid receptor |
| 68 | Multidrug resistance-associated protein 1 |
| 69 | Myeloperoxidase |
| 70 | NADPH oxidase 4 |
| 71 | Nitric oxide synthase, inducible (by homology) |
| 72 | NUAK family SNF1-like kinase 1 |
| 73 | P-Glycoprotein 1 |
| 74 | Phospholipase A2 group 1B |
| 75 | Phospholipase A2 group IIA |
| 76 | PI3-Kinase p110-gamma subunit |
| 77 | PI3-Kinase p85-alpha subunit |
| 78 | Plasminogen |
| 79 | Poly [ADP-ribose] polymerase-1 |
| 80 | Protein kinase N1 |
| 81 | Receptor-type tyrosine-protein phosphatase S |
| 82 | Serine/threonine-protein kinase AKT |
| 83 | Serine/threonine-protein kinase aurora-B |
| 84 | Serine/threonine-protein kinase NEK2 |
| 85 | Serine/threonine-protein kinase NEK6 |
| 86 | Serine/threonine-protein kinase PIM1 |
| 87 | Serine/threonine-protein kinase PLK1 |
| 88 | Sirtuin 1 |
| 89 | Solute carrier family 22 member 12 |
| 90 | Stem cell growth factor receptor |
| 91 | Telomerase reverse transcriptase |
| 92 | Thrombin |
| 93 | Tyrosine-protein kinase receptor FLT3 |
| 94 | Tyrosine-protein kinase receptor UFO |
| 95 | Tyrosine-protein kinase SRC |
| 96 | Tyrosine-protein kinase SYK |
| 97 | Vascular endothelial growth factor receptor 2 |
| 98 | Vasopressin V2 receptor |
| 99 | Xanthine dehydrogenase |

**Table S11. Results of Swiss Target Prediction for Compound 8.**

| **NO.** | **Name** |
| --- | --- |
| 1 | Acetylcholinesterase |
| 2 | ADAMTS5 |
| 3 | Aldo-keto reductase family 1 member C1 |
| 4 | Aldo-keto reductase family 1 member C3 |
| 5 | Aldose reductase |
| 6 | Arachidonate 5-lipoxygenase |
| 7 | Beta amyloid A4 protein |
| 8 | Beta-glucuronidase |
| 9 | Beta-secretase 1 |
| 10 | Carbonic anhydrase I |
| 11 | Carbonic anhydrase II |
| 12 | Carbonic anhydrase III |
| 13 | Carbonic anhydrase IV |
| 14 | Carbonic anhydrase IX |
| 15 | Carbonic anhydrase VA |
| 16 | Carbonic anhydrase VB |
| 17 | Carbonic anhydrase VI |
| 18 | Carbonic anhydrase VII |
| 19 | Carbonic anhydrase XII |
| 20 | Carbonic anhydrase XIII |
| 21 | Carbonic anhydrase XIV |
| 22 | Carbonyl reductase [NADPH] 1 |
| 23 | Casein kinase II alpha |
| 24 | Caspase 8 |
| 25 | C-C Motif chemokine ligand 4 |
| 26 | CDK9/cyclin T1 |
| 27 | c-Jun N-terminal kinase 1 |
| 28 | c-Jun N-terminal kinase 2 |
| 29 | Cyclin-dependent kinase 1/cyclin B1 |
| 30 | Cyclin-dependent kinase 2/cyclin A |
| 31 | Cyclin-dependent kinase 2/cyclin E1 |
| 32 | Cyclin-dependent kinase 4/cyclin D1 |
| 33 | Cyclooxygenase-2 |
| 34 | Cytochrome P450 1A2 |
| 35 | Dihydroorotate dehydrogenase |
| 36 | DNA-(apurinic or apyrimidinic site) lyase |
| 37 | Dynamin-1 |
| 38 | Ephrin receptor |
| 39 | Epidermal growth factor receptor erbB1 |
| 40 | Estradiol 17-beta-dehydrogenase 3 |
| 41 | Estrogen receptor alpha |
| 42 | Estrogen receptor beta |
| 43 | Focal adhesion kinase 1 |
| 44 | G-Protein-coupled receptor kinase 6 |
| 45 | Glutathione reductase |
| 46 | Glycogen synthase kinase-3 beta |
| 47 | G-Protein coupled receptor 35 |
| 48 | Heat shock 70 kDa protein 1 |
| 49 | Hepatocyte growth factor receptor |
| 50 | Hypoxia-inducible factor 1 alpha |
| 51 | Insulin receptor |
| 52 | Insulin-like growth factor I receptor |
| 53 | Interleukin 37 |
| 54 | Kinesin-1 heavy chain/ tyrosine-protein kinase receptor RET |
| 55 | LDL-associated phospholipase A2 |
| 56 | Mediterranean Fever Gene |
| 57 | Mitogen-activated protein kinase kinase kinase 8 |
| 58 | Monoamine oxidase A |
| 59 | Nerve growth factor receptor Trk-A |
| 60 | Neuronal acetylcholine receptor protein alpha-7 subunit |
| 61 | NUAK family SNF1-like kinase 1 |
| 62 | Peroxidasin |
| 63 | Peroxisome proliferator-activated receptor gamma |
| 64 | Phosphodiesterase 5A |
| 65 | PI3-Kinase p110-alpha subunit |
| 66 | PI3-Kinase p110-beta subunit |
| 67 | PI3-Kinase p110-delta subunit |
| 68 | PI3-Kinase p110-gamma subunit |
| 69 | Plasminogen activator inhibitor-1 |
| 70 | Platelet-derived growth factor receptor alpha |
| 71 | Platelet-derived growth factor receptor beta |
| 72 | Poly [ADP-ribose] polymerase-1 |
| 73 | Protein tyrosine kinase 2 beta |
| 74 | Receptor protein-tyrosine kinase erbB-2 |
| 75 | Ribosomal protein S6 kinase alpha 3 |
| 76 | Serine/threonine-protein kinase AKT |
| 77 | Serine/threonine-protein kinase Aurora-A |
| 78 | Serine/threonine-protein kinase Aurora-B |
| 79 | Serine/threonine-protein kinase B-raf |
| 80 | Serine/threonine-protein kinase Chk1 |
| 81 | Serine/threonine-protein kinase mTOR |
| 82 | Serine/threonine-protein kinase PLK1 |
| 83 | Serine/threonine-protein kinase PLK4 |
| 84 | Serine/threonine-protein kinase RAF |
| 85 | Squalene monooxygenase (by homology) |
| 86 | Steroid 5-alpha-reductase 1 |
| 87 | Subtilisin/kexin type 7 |
| 88 | Troponin, cardiac muscle |
| 89 | Tyrosine-protein kinase ABL |
| 90 | Tyrosine-protein kinase FGR (by homology) |
| 91 | Tyrosine-protein kinase HCK |
| 92 | Tyrosine-protein kinase Lyn (by homology) |
| 93 | Tyrosine-protein kinase SRC |
| 94 | Tyrosine-protein kinase TIE-2 |
| 95 | Vascular endothelial growth factor receptor 1 |
| 96 | Vascular endothelial growth factor receptor 2 |
| 97 | Vascular endothelial growth factor receptor 3 |
| 98 | Voltage-gated potassium channel subunit Kv1.5 |
| 99 | Xanthine dehydrogenase |

**Table S12. Results of Swiss Target Prediction for Compound 9.**

| **No.** | **Name** |
| --- | --- |
| 1 | 2'-Deoxynucleoside 5'-phosphate N-hydrolase 1 |
| 2 | Actin Beta |
| 3 | Adenosine A1 receptor |
| 4 | Adenosine A3 receptor |
| 5 | AICAR transformylase |
| 6 | Aldose reductase (by homology) |
| 7 | Aminopeptidase A |
| 8 | Angiotensin-converting enzyme |
| 9 | Asparagine synthetase |
| 10 | ATP-citrate synthase |
| 11 | Bifunctional protein NCOAT |
| 12 | Breast cancer type 1 susceptibility protein |
| 13 | Caspase 4 |
| 14 | Caspase-1 |
| 15 | Caspase-2 |
| 16 | Caspase-3 |
| 17 | Caspase-6 |
| 18 | Caspase-7 |
| 19 | Caspase-8 |
| 20 | Cathepsin D |
| 21 | Cathepsin E |
| 22 | Cathepsin S |
| 23 | Cyclooxygenase-1 |
| 24 | Dihydrofolate reductase |
| 25 | Disks large homolog 4 |
| 26 | DNA (cytosine-5)-methyltransferase 1 |
| 27 | DNA (cytosine-5)-methyltransferase 3B |
| 28 | DNA topoisomerase I |
| 29 | Endo-beta-N-acetylglucosaminidase |
| 30 | Endothelin-converting enzyme 1 |
| 31 | Eukaryotic translation initation factor |
| 32 | EZH2/SUZ12/EED/RBBP7/RBBP4 |
| 33 | Folylpoly-gamma-glutamate synthetase |
| 34 | G-Protein-coupled receptor kinase 6 |
| 35 | Galectin-3 |
| 36 | GAR transformylase |
| 37 | Glucosamine-fructose-6-phosphate aminotransferase [isomerizing] 1 |
| 38 | Glutamate carboxypeptidase II |
| 39 | Glutathione S-transferase Pi |
| 40 | Glyoxalase I |
| 41 | G-Protein coupled receptor 35 |
| 42 | Hematopoietic cell protein-tyrosine phosphatase 70Z-PEP |
| 43 | Hexokinase type I |
| 44 | Hexokinase type II |
| 45 | Histone-arginine methyltransferase CARM1 |
| 46 | Histone-lysine N-methyltransferase EZH1 |
| 47 | Histone-lysine N-methyltransferase MLL |
| 48 | Histone-lysine N-methyltransferase SETDB1 |
| 49 | Histone-lysine N-methyltransferase SUV39H1 |
| 50 | Histone-lysine N-methyltransferase, H3 lysine-79 specific |
| 51 | Histone-lysine N-methyltransferase, H3 lysine-9 specific 3 |
| 52 | Histone-lysine N-methyltransferase, H3 lysine-9 specific 5 |
| 53 | Hypoxanthine-guanine phosphoribosyltransferase |
| 54 | Indoleamine 2,3-dioxygenase |
| 55 | Indolethylamine N-methyltransferase |
| 56 | Inosine-5'-monophosphate dehydrogenase 2 |
| 57 | Interleukin 1 receptor antagonist |
| 58 | Interleukin 13 |
| 59 | Interleukin-8 receptor B |
| 60 | Leucine aminopeptidase |
| 61 | Leukocyte adhesion molecule-1 |
| 62 | Low affinity sodium-glucose cotransporter |
| 63 | Lysosomal alpha-glucosidase |
| 64 | Maltase-glucoamylase |
| 65 | Mannose receptor C-type 1 |
| 66 | MAP kinase p38 alpha |
| 67 | Metabotropic glutamate receptor 2 (by homology) |
| 68 | Muscle glycogen phosphorylase |
| 69 | NAALADase II |
| 70 | Neprilysin (by homology) |
| 71 | Nicotinamide N-methyltransferase |
| 72 | N-lysine methyltransferase SMYD2 |
| 73 | Nucleotide-binding oligomerization domain-containing protein 2 |
| 74 | PI3-Kinase p110-alpha subunit |
| 75 | PI3-Kinase p110-gamma subunit |
| 76 | Protein arginine N-methyltransferase 3 |
| 77 | Protein arginine N-methyltransferase 5 |
| 78 | Protein kinase C alpha |
| 79 | Protein tyrosine phosphatase type IVA 1 |
| 80 | Protein tyrosine phosphatase type IVA 2 |
| 81 | Protein-arginine N-methyltransferase 1 |
| 82 | Protein-tyrosine phosphatase 4A3 |
| 83 | P-selectin |
| 84 | Purinergic receptor P2Y1 |
| 85 | Serine/threonine-protein kinase aurora-A |
| 86 | Serine/threonine-protein kinase PIM1 |
| 87 | Serine/threonine-protein kinase PLK1 |
| 88 | Sialidase 2 |
| 89 | Sialidase 3 |
| 90 | Sialidase 4 |
| 91 | Steroid 5-alpha-reductase 1 |
| 92 | Thymidylate synthase |
| 93 | Transketolase |
| 94 | Tyrosine-protein kinase LCK |
| 95 | Tyrosine-protein kinase SRC |
| 96 | Tyrosine-protein kinase ZAP-70 |
| 97 | Tyrosyl-DNA phosphodiesterase 1 |
| 98 | Tyrosyl-tRNA synthetase |
| 99 | Vascular endothelial growth factor receptor 2 |

**Table S13. Results of Swiss Target Prediction for Compound 10.**

| **No.** | **Name** |
| --- | --- |
| 1 | 6-phosphofructo-2-kinase/fructose-2,6-bisphosphatase 3 |
| 2 | Acetylcholinesterase |
| 3 | Adenosine A1 receptor (by homology) |
| 4 | Adenosine A2a receptor (by homology) |
| 5 | Adenosine A3 receptor |
| 6 | Aldehyde reductase (by homology) |
| 7 | Aldo-keto reductase family 1 member C1 (by homology) |
| 8 | Aldo-keto reductase family 1 member C2 (by homology) |
| 9 | Aldo-keto reductase family 1 member C4 (by homology) |
| 10 | Aldo-keto-reductase family 1 member C3 (by homology) |
| 11 | Aldose reductase (by homology) |
| 12 | ALK tyrosine kinase receptor |
| 13 | AMY1C |
| 14 | Angiotensin-converting enzyme 2 |
| 15 | Arachidonate 15-lipoxygenase |
| 16 | Arachidonate 5-lipoxygenase |
| 17 | Arginase-1 (by homology) |
| 18 | Aryl hydrocarbon receptor |
| 19 | ATP-binding cassette sub-family G member 2 |
| 20 | Beta amyloid A4 protein |
| 21 | Beta-secretase 1 |
| 22 | Butyrylcholinesterase |
| 23 | CaM kinase II beta |
| 24 | Carbonic anhydrase I |
| 25 | Carbonic anhydrase II |
| 26 | Carbonic anhydrase III |
| 27 | Carbonic anhydrase IV |
| 28 | Carbonic anhydrase IX |
| 29 | Carbonic anhydrase VA |
| 30 | Carbonic anhydrase VI |
| 31 | Carbonic anhydrase VII |
| 32 | Carbonic anhydrase XII |
| 33 | Carbonic anhydrase XIII (by homology) |
| 34 | Carbonic anhydrase XIV |
| 35 | C-Reactive protein |
| 36 | C-X-C Motif chemokine ligand 8 |
| 37 | Cyclin-dependent kinase 1 |
| 38 | Cyclin-dependent kinase 6 |
| 39 | Cyclooxygenase-2 |
| 40 | Cytochrome P450 19A1 |
| 41 | Cytochrome P450 1A2 |
| 42 | Cytochrome P450 1B1 |
| 43 | Death-associated protein kinase 1 |
| 44 | Delta opioid receptor (by homology) |
| 45 | DNA topoisomerase II alpha |
| 46 | DNA (apurinic or apyrimidinic site) lyase |
| 47 | DNA-3-methyladenine glycosylase |
| 48 | Epidermal growth factor receptor erbB1 |
| 49 | Estradiol 17-beta-dehydrogenase 1 |
| 50 | Estradiol 17-beta-dehydrogenase 2 |
| 51 | Estrogen receptor alpha |
| 52 | Estrogen receptor beta |
| 53 | Estrogen-related receptor alpha |
| 54 | Focal adhesion kinase 1 |
| 55 | G-Protein-coupled receptor kinase 6 |
| 56 | Glycogen synthase kinase-3 beta |
| 57 | Glyoxalase I |
| 58 | G-Protein coupled receptor 35 |
| 59 | Hepatocyte growth factor receptor |
| 60 | Induced myeloid leukemia cell differentiation protein Mcl-1 |
| 61 | Insulin receptor |
| 62 | Insulin-like growth factor I receptor |
| 63 | Interleukin 33 |
| 64 | Lysine-specific demethylase 4D-like |
| 65 | Matrix metalloproteinase 13 |
| 66 | Matrix metalloproteinase 2 |
| 67 | Matrix metalloproteinase 3 |
| 68 | Matrix metalloproteinase 9 |
| 69 | Monoamine oxidase A |
| 70 | Mu opioid receptor (by homology) |
| 71 | Multidrug resistance-associated protein 1 |
| 72 | Myeloperoxidase |
| 73 | NADPH oxidase 4 |
| 74 | NEDD8-activating enzyme E1 regulatory subunit |
| 75 | Nitric oxide synthase, inducible |
| 76 | NUAK family SNF1-like kinase 1 |
| 77 | Ornithine decarboxylase |
| 78 | P-Glycoprotein 1 |
| 79 | Phospholipase A2 group 1B |
| 80 | PI3-Kinase p85-alpha subunit |
| 81 | Plasminogen |
| 82 | PYD and CARD domain containing |
| 83 | Receptor-type tyrosine-protein phosphatase S |
| 84 | Serine/threonine-protein kinase aurora-B |
| 85 | Serine/threonine-protein kinase NEK2 |
| 86 | Serine/threonine-protein kinase NEK6 |
| 87 | Serine/threonine-protein kinase PIM1 |
| 88 | Serine/threonine-protein kinase PLK1 |
| 89 | Solute carrier family 22 member 12 |
| 90 | Stem cell growth factor receptor |
| 91 | Taste receptor type 2 member 31 |
| 92 | Telomerase reverse transcriptase |
| 93 | Thrombin |
| 94 | Transthyretin |
| 95 | Tyrosine-protein kinase receptor FLT3 |
| 96 | Tyrosine-protein kinase SRC |
| 97 | Tyrosine-protein kinase SYK |
| 98 | Vascular endothelial growth factor receptor 2 |
| 99 | Xanthine dehydrogenase |

**Table S14. Results of Swiss Target Prediction for Compound 11.**

| **No.** | **Name** |
| --- | --- |
| 1 | Acetylcholinesterase |
| 2 | Adenosine A1 receptor (by homology) |
| 3 | Adenosine A2a receptor (by homology) |
| 4 | Adenosine A3 receptor |
| 5 | Adrenergic receptor alpha-2 |
| 6 | Aldehyde dehydrogenase |
| 7 | Aldose reductase (by homology) |
| 8 | Alpha-2a adrenergic receptor |
| 9 | Arachidonate 5-lipoxygenase |
| 10 | ATP-binding cassette sub-family G member 2 |
| 11 | Beta amyloid A4 protein |
| 12 | Beta-adrenergic receptor kinase 2 |
| 13 | cAMP-dependent protein kinase alpha-catalytic subunit |
| 14 | Carbonic anhydrase II |
| 15 | Carbonic anhydrase IV |
| 16 | Carbonic anhydrase IX |
| 17 | Carbonic anhydrase VII |
| 18 | Carbonic anhydrase XII |
| 19 | Carbonic anhydrase XIII |
| 20 | Caspase 5 |
| 21 | Catechol O-methyltransferase |
| 22 | C-C Motif chemokine ligand 3 |
| 23 | CCR4-NOT transcription complex subunit 7 |
| 24 | C-X-C Motif chemokine ligand 10 |
| 25 | Cyclin-dependent kinase 1/cyclin B1 |
| 26 | Cyclin-dependent kinase 2/cyclin A |
| 27 | Cyclin-dependent kinase 2/cyclin E |
| 28 | Cyclin-dependent kinase 4 |
| 29 | Cyclin-dependent kinase 4/cyclin D1 |
| 30 | Cyclooxygenase-2 |
| 31 | Cytochrome b-245 beta chain |
| 32 | Cytochrome P450 1A1 |
| 33 | Cytochrome P450 1B1 |
| 34 | Dopamine D2 receptor (by homology) |
| 35 | Dual specificity protein phosphatase 3 |
| 36 | Egl nine homolog 1 |
| 37 | Epidermal growth factor receptor erbB1 |
| 38 | Equilibrative nucleoside transporter 1 |
| 39 | Fructose-1,6-bisphosphatase |
| 40 | G-Protein-coupled receptor kinase 4 |
| 41 | G-Protein-coupled receptor kinase 5 |
| 42 | G-Protein-coupled receptor kinase 7 |
| 43 | G-Protein coupled receptor kinase 2 |
| 44 | Heat shock protein HSP 90-alpha |
| 45 | Heat shock protein HSP 90-beta |
| 46 | Histone deacetylase 1 |
| 47 | Histone deacetylase 10 |
| 48 | Histone deacetylase 2 |
| 49 | Histone deacetylase 3 |
| 50 | Histone deacetylase 6 |
| 51 | Histone deacetylase 8 |
| 52 | Inhibitor of nuclear factor kappa B kinase beta subunit |
| 53 | Integrin alpha-4/beta-1 |
| 54 | Integrin alpha-5/beta-1 |
| 55 | Integrin alpha-IIb/beta-3 |
| 56 | Integrin alpha-V/beta-3 |
| 57 | Integrin alpha-V/beta-6 |
| 58 | Interleukin 1 receptor accessory protein |
| 59 | Interleukin-2 |
| 60 | Interleukin-8 receptor B |
| 61 | Kallikrein 1 |
| 62 | Kallikrein 2 |
| 63 | Lymphocyte differentiation antigen CD38 |
| 64 | Metastin receptor |
| 65 | Multidrug resistance-associated protein 1 |
| 66 | NADPH oxidase 4 |
| 67 | Neuromedin-U receptor 2 |
| 68 | P-Glycoprotein 1 |
| 69 | Phosphodiesterase 5A |
| 70 | PI3-Kinase p110-alpha subunit |
| 71 | Plasminogen |
| 72 | Plasminogen activator inhibitor-1 |
| 73 | Prostaglandin E synthase |
| 74 | Proteasome assembly chaperone 3 |
| 75 | Protein kinase C (PKC) |
| 76 | Protein kinase C alpha |
| 77 | Protein kinase C beta |
| 78 | Protein kinase C delta |
| 79 | Protein kinase C epsilon |
| 80 | Protein kinase C eta |
| 81 | Protein kinase C gamma |
| 82 | Quinone reductase 2 |
| 83 | Rhodopsin kinase |
| 84 | Ribosomal protein S6 kinase alpha 3 |
| 85 | Serine/threonine-protein kinase Chk1 |
| 86 | Serine/threonine-protein kinase Chk2 |
| 87 | Serine/threonine-protein kinase RAF |
| 88 | Sigma opioid receptor |
| 89 | Squalene monooxygenase (by homology) |
| 90 | SUMO-activating enzyme |
| 91 | Telomerase reverse transcriptase |
| 92 | Thrombin and coagulation factor X |
| 93 | TNF-alpha |
| 94 | Transitional endoplasmic reticulum ATPase |
| 95 | Troponin, cardiac muscle |
| 96 | Tumor suppressor p53/oncoprotein Mdm2 |
| 97 | Urokinase-type plasminogen activator |
| 98 | Voltage-gated potassium channel subunit Kv1.3 |
| 99 | Xanthine dehydrogenase |

**Table S15. Results of Swiss Target Prediction for Compound 12.**

| **No.** | **Name** |
| --- | --- |
| 1 | Acetylcholinesterase |
| 2 | Adenosine A1 receptor (by homology) |
| 3 | Adenosine A2a receptor (by homology) |
| 4 | Adenosine A3 receptor |
| 5 | Adrenergic receptor alpha-2 |
| 6 | Adrenergic receptor beta |
| 7 | Aldehyde dehydrogenase |
| 8 | Aldose reductase |
| 9 | Alpha-2a adrenergic receptor |
| 10 | Arachidonate 5-lipoxygenase |
| 11 | ATP-binding cassette sub-family G member 2 |
| 12 | Beta amyloid A4 protein |
| 13 | Beta-1 adrenergic receptor |
| 14 | cAMP-dependent protein kinase alpha-catalytic subunit |
| 15 | Carbonic anhydrase II |
| 16 | Carbonic anhydrase IV |
| 17 | Carbonic anhydrase VII |
| 18 | Carbonic anhydrase XII |
| 19 | Carbonic anhydrase XIII |
| 20 | Catechol O-methyltransferase |
| 21 | C-C Motif chemokine ligand 2 |
| 22 | CCR4-NOT transcription complex subunit 7 |
| 23 | Cyclin-dependent kinase 1/cyclin B1 |
| 24 | Cyclooxygenase-2 |
| 25 | Cytochrome P450 19A1 |
| 26 | Cytochrome P450 1B1 |
| 27 | Death-associated protein kinase 1 |
| 28 | DNA topoisomerase II alpha |
| 29 | Dopamine D3 receptor |
| 30 | Dopamine D4 receptor |
| 31 | Dual specificity protein phosphatase 3 |
| 32 | Dynamin-2 |
| 33 | Egl nine homolog 1 |
| 34 | Equilibrative nucleoside transporter 1 |
| 35 | Estradiol 17-beta-dehydrogenase 2 |
| 36 | Focal adhesion kinase 1 |
| 37 | Gasdermin D |
| 38 | Glyceraldehyde-3-phosphate dehydrogenase |
| 39 | Glycogen synthase kinase-3 beta |
| 40 | Glyoxalase I |
| 41 | G-Protein coupled receptor 35 |
| 42 | Heat shock protein HSP 90-alpha |
| 43 | Heat shock protein HSP 90-beta |
| 44 | Induced myeloid leukemia cell differentiation protein Mcl-1 |
| 45 | Inhibitor of nuclear factor kappa B kinase beta subunit |
| 46 | Insulin receptor |
| 47 | Insulin-like growth factor I receptor |
| 48 | Integrin alpha-4/beta-1 |
| 49 | Integrin alpha-5/beta-1 |
| 50 | Integrin alpha-IIb/beta-3 |
| 51 | Integrin alpha-V/beta-3 |
| 52 | Integrin alpha-V/beta-6 |
| 53 | Interleukin 2 |
| 54 | Interleukin-2 |
| 55 | Liver glycogen phosphorylase |
| 56 | Lymphocyte differentiation antigen CD38 |
| 57 | Lysine-specific demethylase 4D-like |
| 58 | Matrix metalloproteinase 13 |
| 59 | Metastin receptor |
| 60 | Microtubule-associated protein tau |
| 61 | Monoamine oxidase A |
| 62 | Myeloperoxidase |
| 63 | Myosin light chain kinase, smooth muscle |
| 64 | NADPH oxidase 4 |
| 65 | Neuromedin-U receptor 2 |
| 66 | Phosphodiesterase 5A |
| 67 | PI3-Kinase p110-alpha subunit |
| 68 | PI3-Kinase p85-alpha subunit |
| 69 | Plasminogen |
| 70 | Plasminogen activator inhibitor-1 |
| 71 | Protein kinase C (PKC) |
| 72 | Protein kinase C alpha |
| 73 | Protein kinase C beta |
| 74 | Protein kinase C delta |
| 75 | Protein kinase C epsilon |
| 76 | Protein kinase C eta |
| 77 | Protein kinase C gamma |
| 78 | Quinone reductase 2 |
| 79 | Ribosomal protein S6 kinase alpha 3 |
| 80 | Serine/threonine-protein kinase aurora-B |
| 81 | Serine/threonine-protein kinase Chk1 |
| 82 | Serine/threonine-protein kinase Chk2 |
| 83 | Serine/threonine-protein kinase PIM1 |
| 84 | Serine/threonine-protein kinase RAF |
| 85 | Serine/threonine-protein kinase WEE1 |
| 86 | Squalene monooxygenase (by homology) |
| 87 | Telomerase reverse transcriptase |
| 88 | Thrombin and coagulation factor X |
| 89 | TNF-alpha |
| 90 | Toll-like receptor 9 |
| 91 | Transitional endoplasmic reticulum ATPase |
| 92 | Troponin, cardiac muscle |
| 93 | Tyrosine-protein kinase receptor FLT3 |
| 94 | Tyrosine-protein kinase SYK |
| 95 | Tyrosyl-DNA phosphodiesterase 1 |
| 96 | Vascular endothelial growth factor receptor 2 |
| 97 | Vasopressin V2 receptor |
| 98 | Voltage-gated potassium channel subunit Kv1.3 |
| 99 | Xanthine dehydrogenase |

**Table S16. Results of Swiss Target Prediction for Compound 13.**

| **No.** | **Name** |
| --- | --- |
| 1 | Absent in melanoma 2 |
| 2 | Acetylcholinesterase |
| 3 | Adenosine A1 receptor (by homology) |
| 4 | Adrenergic receptor alpha-2 |
| 5 | Aldehyde dehydrogenase |
| 6 | Aldose reductase (by homology) |
| 7 | Alpha-2a adrenergic receptor |
| 8 | Androgen receptor |
| 9 | Apoptosis regulator Bcl-2 |
| 10 | Apoptosis regulator Bcl-X |
| 11 | Arachidonate 5-lipoxygenase |
| 12 | Bcl-2-related protein A1 |
| 13 | Beta-1 adrenergic receptor |
| 14 | Beta-adrenergic receptor kinase 2 |
| 15 | Beta-secretase 1 |
| 16 | cAMP-dependent protein kinase alpha-catalytic subunit |
| 17 | Cannabinoid receptor 2 |
| 18 | Carbonic anhydrase I |
| 19 | Carbonic anhydrase II |
| 20 | Carbonic anhydrase III |
| 21 | Carbonic anhydrase IV |
| 22 | Carbonic anhydrase IX |
| 23 | Carbonic anhydrase VB |
| 24 | Carbonic anhydrase VI |
| 25 | Carbonic anhydrase VII |
| 26 | Carbonic anhydrase XII |
| 27 | Carbonic anhydrase XIII |
| 28 | Carbonic anhydrase XIV |
| 29 | Catechol O-methyltransferase |
| 30 | Cyclooxygenase-2 |
| 31 | Cytochrome P450 1A2 |
| 32 | DNA topoisomerase I |
| 33 | Dopamine transporter (by homology) |
| 34 | Dual specificity protein phosphatase 3 |
| 35 | Dynamin-2 |
| 36 | Equilibrative nucleoside transporter 1 |
| 37 | Estrogen receptor alpha |
| 38 | Estrogen receptor beta |
| 39 | G-Protein-coupled receptor kinase 4 |
| 40 | G-Protein-coupled receptor kinase 5 |
| 41 | G-Protein-coupled receptor kinase 6 |
| 42 | G-Protein-coupled receptor kinase 7 |
| 43 | G-Protein coupled receptor kinase 2 |
| 44 | Heat shock protein HSP 90-alpha |
| 45 | Heat shock protein HSP 90-beta |
| 46 | Heme oxygenase 1 |
| 47 | Induced myeloid leukemia cell differentiation protein Mcl-1 |
| 48 | Inhibitor of nuclear factor kappa B kinase beta subunit |
| 49 | Integrin alpha-5/beta-1 |
| 50 | Integrin alpha-IIb/beta-3 |
| 51 | Integrin alpha-V/beta-3 |
| 52 | Integrin alpha-V/beta-6 |
| 53 | Interleukin 18 |
| 54 | Interleukin 4 |
| 55 | Interleukin-2 |
| 56 | Lymphocyte differentiation antigen CD38 |
| 57 | Metastin receptor |
| 58 | Monoamine oxidase A |
| 59 | Monoamine oxidase B |
| 60 | Mu opioid receptor |
| 61 | Multidrug resistance-associated protein 1 |
| 62 | NAD-dependent deacetylase sirtuin 1 |
| 63 | NADPH oxidase 4 |
| 64 | Neuromedin-U receptor 2 |
| 65 | P-Glycoprotein 1 (by homology) |
| 66 | Phosphodiesterase 4D |
| 67 | Phosphodiesterase 5A |
| 68 | Plasminogen activator inhibitor-1 |
| 69 | Protein kinase C (PKC) |
| 70 | Protein kinase C alpha |
| 71 | Protein kinase C beta |
| 72 | Protein kinase C delta |
| 73 | Protein kinase C epsilon |
| 74 | Protein kinase C eta |
| 75 | Protein kinase C gamma |
| 76 | Quinone reductase 2 |
| 77 | Rhodopsin kinase |
| 78 | Ribosomal protein S6 kinase alpha 3 |
| 79 | Serine/threonine-protein kinase Chk1 |
| 80 | Serine/threonine-protein kinase Chk2 |
| 81 | Serine/threonine-protein kinase RAF |
| 82 | Serine/threonine-protein kinase WEE1 |
| 83 | Serotonin transporter (by homology) |
| 84 | Sodium/glucose cotransporter 1 |
| 85 | Sodium/glucose cotransporter 2 |
| 86 | Squalene monooxygenase (by homology) |
| 87 | Thrombin |
| 88 | Thrombin and coagulation factor X |
| 89 | TNF-alpha |
| 90 | Transitional endoplasmic reticulum ATPase |
| 91 | Troponin, cardiac muscle |
| 92 | Tumor necrosis factor |
| 93 | Tyrosinase |
| 94 | Tyrosyl-DNA phosphodiesterase 1 |
| 95 | Voltage-gated potassium channel subunit Kv1.3 |
| 96 | Voltage-gated potassium channel subunit Kv1.5 |
| 97 | Xanthine dehydrogenase |

**Table S17: GO Enrichment Entry.**

| **Category** | **Description** |
| --- | --- |
| GO Biological Process | Inflammatory response |
| GO Biological Process | Immune response |
| GO Biological Process | Immune system process |
| GO Biological Process | Regulation of cytokine production |
| GO Biological Process | Defense response |
| GO Biological Process | Response to other organisms |
| GO Biological Process | Biological process involved in interspecies interaction between organisms |
| GO Biological Process | Positive regulation of cytokine production |
| GO Biological Process | Response to cytokine |
| GO Biological Process | Defense response to other organisms |
| GO Biological Process | Cellular response to cytokine stimulus |
| GO Biological Process | Response to external stimulus |
| GO Biological Process | Response to stress |
| GO Biological Process | Positive regulation of gene expression |
| GO Biological Process | Response to bacterium |
| GO Biological Process | Cytokine-mediated signaling pathway |
| GO Biological Process | Regulation of inflammatory response |
| GO Biological Process | Response to organic substance |
| GO Biological Process | Regulation of response to external stimulus |
| GO Biological Process | Innate immune response |
| GO Biological Process | Response to lipopolysaccharide |
| GO Biological Process | Cellular response to organic substance |
| GO Biological Process | Regulation of defense response |
| GO Biological Process | Cellular response to chemical stimulus |
| GO Biological Process | Cellular response to lipopolysaccharide |
| GO Biological Process | Positive regulation of interleukin-1 beta production |
| GO Biological Process | Positive regulation of metabolic process |
| GO Biological Process | Regulation of immune system process |
| GO Biological Process | Positive regulation of macromolecule metabolic process |
| GO Biological Process | Positive regulation of multicellular organismal process |
| GO Biological Process | Positive regulation of immune system process |
| GO Biological Process | Regulation of multicellular organismal process |
| GO Biological Process | Regulation of response to stress |
| GO Biological Process | Regulation of interleukin-1 production |
| GO Biological Process | Response to chemical |
| GO Biological Process | Regulation of production of molecular mediator of immune response |
| GO Biological Process | Regulation of response to stimulus |
| GO Biological Process | Positive regulation of response to external stimulus |
| GO Biological Process | Positive regulation of inflammatory response |
| GO Biological Process | Positive regulation of response to stimulus |
| GO Biological Process | Positive regulation of defense response |
| GO Biological Process | Positive regulation of immune effector process |
| GO Biological Process | Response to lipid |
| GO Biological Process | Cellular response to stimulus |
| GO Biological Process | Regulation of immune effector process |
| GO Biological Process | Regulation of immune response |
| GO Biological Process | Response to oxygen-containing compound |
| GO Biological Process | Regulation of molecular function |
| GO Biological Process | Positive regulation of production of molecular mediator of immune response |
| GO Biological Process | Cellular response to lipid |
| GO Biological Process | Pyroptosis |
| GO Biological Process | Programmed cell death |
| GO Biological Process | Positive regulation of molecular function |
| GO Biological Process | Regulation of cytokine production involved in immune response |
| GO Biological Process | Positive regulation of biological process |
| GO Biological Process | Response to stimulus |
| GO Biological Process | Signal transduction |
| GO Biological Process | Positive regulation of protein metabolic process |
| GO Biological Process | Cellular response to oxygen-containing compound |
| GO Biological Process | Positive regulation of leukocyte activation |
| GO Biological Process | Cell communication |
| GO Biological Process | Regulation of leukocyte activation |
| GO Biological Process | Cell activation |
| GO Biological Process | Negative regulation of cytokine production |
| GO Biological Process | Regulation of interleukin-6 production |
| GO Biological Process | Response to virus |
| GO Biological Process | Regulation of chemokine production |
| GO Biological Process | Apoptotic process |
| GO Biological Process | Positive regulation of cysteine-type endopeptidase activity |
| GO Biological Process | Regulation of gene expression |
| GO Biological Process | Positive regulation of immune response |
| GO Biological Process | Positive regulation of catalytic activity |
| GO Biological Process | Negative regulation of gene expression |
| GO Biological Process | Regulation of macromolecule metabolic process |
| GO Biological Process | Regulation of protein metabolic process |
| GO Biological Process | Regulation of leukocyte proliferation |
| GO Biological Process | Regulation of multicellular organismal development |
| GO Biological Process | Regulation of cell population proliferation |
| GO Biological Process | Positive regulation of interleukin-6 production |
| GO Biological Process | Positive regulation of lymphocyte activation |
| GO Biological Process | Positive regulation of chemokine production |
| GO Biological Process | Leukocyte activation |
| GO Biological Process | Cell surface receptor signaling pathway |
| GO Biological Process | Regulation of leukocyte mediated immunity |
| GO Biological Process | Negative regulation of response to stimulus |
| GO Biological Process | Regulation of lymphocyte activation |
| GO Biological Process | Positive regulation of cellular process |
| GO Biological Process | Regulation of DNA-binding transcription factor activity |
| GO Biological Process | Myeloid leukocyte activation |
| GO Biological Process | Positive regulation of nitrogen compound metabolic process |
| GO Biological Process | Defense response to bacterium |
| GO Biological Process | Positive regulation of proteolysis |
| GO Biological Process | Regulation of cell death |
| GO Biological Process | Positive regulation of signal transduction |
| GO Biological Process | Regulation of tumor necrosis factor production |
| GO Biological Process | Positive regulation of leukocyte proliferation |
| GO Biological Process | Regulation of catalytic activity |
| GO Biological Process | Regulation of signal transduction |
| GO Biological Process | Regulation of leukocyte migration |
| GO Biological Process | Regulation of programmed cell death |
| GO Biological Process | Positive regulation of hydrolase activity |
| GO Biological Process | Regulation of mononuclear cell proliferation |
| GO Biological Process | Positive regulation of lymphocyte proliferation |
| GO Biological Process | Regulation of adaptive immune response |
| GO Biological Process | Defense response to virus |
| GO Biological Process | Regulation of leukocyte differentiation |
| GO Biological Process | Regulation of I-kappaB kinase/NF-kappaB signaling |
| GO Biological Process | Regulation of cell communication |
| GO Biological Process | Regulation of signaling |
| GO Biological Process | Regulation of apoptotic process |
| GO Biological Process | Positive regulation of DNA-binding transcription factor activity |
| GO Biological Process | Regulation of hemopoiesis |
| GO Biological Process | Response to interferon-gamma |
| GO Biological Process | Positive regulation of cysteine-type endopeptidase activity involved in apoptotic process |
| GO Biological Process | Negative regulation of multicellular organismal process |
| GO Biological Process | Positive regulation of tyrosine phosphorylation of STAT protein |
| GO Biological Process | Regulation of leukocyte cell-cell adhesion |
| GO Biological Process | Regulation of cell differentiation |
| GO Biological Process | Regulation of lymphocyte proliferation |
| GO Biological Process | Pattern recognition receptor signaling pathway |
| GO Biological Process | Positive regulation of tumor necrosis factor production |
| GO Biological Process | Positive regulation of cell population proliferation |
| GO Biological Process | Positive regulation of peptidyl-tyrosine phosphorylation |
| GO Biological Process | Regulation of proteolysis |
| GO Biological Process | Cellular response to interferon-gamma |
| GO Biological Process | Apoptotic signaling pathway |
| GO Biological Process | Regulation of cell-cell adhesion |
| GO Biological Process | Regulation of protein phosphorylation |
| GO Biological Process | Regulation of developmental process |
| GO Biological Process | Positive regulation of intracellular signal transduction |
| GO Biological Process | Regulation of endopeptidase activity |
| GO Biological Process | Positive regulation of cytokine production involved in immune response |
| GO Biological Process | Regulation of response to cytokine stimulus |
| GO Biological Process | Regulation of interferon-gamma production |
| GO Biological Process | Activation of cysteine-type endopeptidase activity involved in apoptotic process |
| GO Biological Process | Regulation of interleukin-8 production |
| GO Biological Process | Defense response to Gram-positive bacterium |
| GO Biological Process | Regulation of adaptive immune response based on somatic recombination of immune receptors built from immunoglobulin superfamily domains |
| GO Biological Process | Regulation of ERK1 and ERK2 cascade |
| GO Biological Process | Immune effector process |
| GO Biological Process | Regulation of T cell activation |
| GO Biological Process | Positive regulation of protein phosphorylation |
| GO Biological Process | Response to organic cyclic compound |
| GO Biological Process | Leukocyte activation involved in immune response |
| GO Biological Process | Leukocyte migration |
| GO Biological Process | Cellular response to interleukin-1 |
| GO Biological Process | Positive regulation of cellular metabolic process |
| GO Biological Process | Negative regulation of signal transduction |
| GO Biological Process | Regulation of T cell cytokine production |
| GO Biological Process | Regulation of smooth muscle cell proliferation |
| GO Biological Process | Negative regulation of cellular process |
| GO Biological Process | Positive regulation of cell-cell adhesion |
| GO Biological Process | Lipopolysaccharide-mediated signaling pathway |
| GO Biological Process | Regulation of phosphate metabolic process |
| GO Biological Process | Regulation of hydrolase activity |
| GO Biological Process | Negative regulation of biological process |
| GO Biological Process | Regulation of intracellular signal transduction |
| GO Biological Process | Negative regulation of cell differentiation |
| GO Biological Process | Positive regulation of leukocyte migration |
| GO Biological Process | Positive regulation of phosphate metabolic process |
| GO Biological Process | Regulation of cytokine-mediated signaling pathway |
| GO Biological Process | Positive regulation of leukocyte cell-cell adhesion |
| GO Biological Process | Positive regulation of NF-kappaB transcription factor activity |
| GO Biological Process | Positive regulation of developmental process |
| GO Biological Process | Regulation of immunoglobulin production |
| GO Biological Process | Positive regulation of adaptive immune response |
| GO Biological Process | Positive regulation of cell migration |
| GO Biological Process | Regulation of cellular process |
| GO Biological Process | Regulation of lymphocyte mediated immunity |
| GO Biological Process | Positive regulation of smooth muscle cell proliferation |
| GO Biological Process | Regulation of biological process |
| GO Biological Process | Positive regulation of immunoglobulin production |
| GO Biological Process | Cellular response to virus |
| GO Biological Process | Positive regulation of leukocyte differentiation |
| GO Biological Process | Regulation of MAPK cascade |
| GO Biological Process | Regulation of cell adhesion |
| GO Biological Process | Regulation of cytokine production involved in inflammatory response |
| GO Biological Process | Positive regulation of biosynthetic process |
| GO Biological Process | Regulation of cellular metabolic process |
| GO Biological Process | Positive regulation of T cell activation |
| GO Biological Process | Regulation of neuron death |
| GO Biological Process | Myeloid leukocyte migration |
| GO Biological Process | Positive regulation of cell death |
| GO Biological Process | Regulation of cell migration |
| GO Biological Process | Regulation of nitrogen compound metabolic process |
| GO Biological Process | Regulation of type 2 immune response |
| GO Biological Process | Positive regulation of MHC class II biosynthetic process |
| GO Biological Process | Positive regulation of neuroinflammatory response |
| GO Biological Process | Leukocyte activation involved in inflammatory response |
| GO Biological Process | Regulation of neuroinflammatory response |
| GO Biological Process | Macrophage activation |
| GO Biological Process | Granulocyte migration |
| GO Biological Process | Leukocyte chemotaxis |
| GO Biological Process | Positive regulation of programmed cell death |
| GO Biological Process | Positive regulation of interleukin-8 production |
| GO Biological Process | Glial cell activation |
| GO Biological Process | Regulation of primary metabolic process |
| GO Biological Process | Positive regulation of cell differentiation |
| GO Biological Process | Cellular response to mechanical stimulus |
| GO Biological Process | Positive regulation of macromolecule biosynthetic process |
| GO Biological Process | Inflammasome complex assembly |
| GO Biological Process | Regulation of biosynthetic process |
| GO Biological Process | Positive regulation of interferon-gamma production |
| GO Biological Process | Positive regulation of MAPK cascade |
| GO Biological Process | Neutrophil chemotaxis |
| GO Biological Process | Leukocyte differentiation |
| GO Biological Process | Negative regulation of macromolecule metabolic process |
| GO Biological Process | Cellular response to abiotic stimulus |
| GO Biological Process | Negative regulation of cell population proliferation |
| GO Biological Process | Positive regulation of apoptotic process |
| GO Biological Process | Positive regulation of cellular biosynthetic process |
| GO Biological Process | Positive regulation of I-kappaB kinase/NF-kappaB signaling |
| GO Biological Process | Positive regulation of T cell cytokine production |
| GO Biological Process | Negative regulation of NF-kappaB transcription factor activity |
| GO Biological Process | Positive regulation of leukocyte mediated immunity |
| GO Biological Process | Regulation of interleukin-23 production |
| GO Biological Process | Regulation of cellular biosynthetic process |
| GO Biological Process | Positive regulation of B cell activation |
| GO Biological Process | Response to tumor necrosis factor |
| GO Biological Process | Humoral immune response |
| GO Biological Process | Negative regulation of molecular function |
| GO Biological Process | Regulation of macrophage activation |
| GO Biological Process | Regulation of macromolecule biosynthetic process |
| GO Biological Process | Regulation of lymphocyte differentiation |
| GO Biological Process | Regulation of response to biotic stimulus |
| GO Biological Process | Regulation of interleukin-18 production |
| GO Biological Process | Microglial cell activation |
| GO Biological Process | Response to mechanical stimulus |
| GO Biological Process | Regulation of T-helper 2 cell cytokine production |
| GO Biological Process | Positive regulation of T cell proliferation |
| GO Biological Process | Positive regulation of adaptive immune response based on somatic recombination of immune receptors built from immunoglobulin superfamily domains |
| GO Biological Process | Regulation of B cell proliferation |
| GO Biological Process | Extrinsic apoptotic signaling pathway |
| GO Biological Process | Positive regulation of lymphocyte mediated immunity |
| GO Biological Process | Response to abiotic stimulus |
| GO Biological Process | Cellular response to external stimulus |
| GO Biological Process | Negative regulation of DNA-binding transcription factor activity |
| GO Biological Process | Cellular response to tumor necrosis factor |
| GO Biological Process | Positive regulation of interleukin-10 production |
| GO Biological Process | Positive regulation of mononuclear cell migration |
| GO Biological Process | Regulation of interleukin-17 production |
| GO Biological Process | Regulation of transport |
| GO Biological Process | Negative regulation of immune effector process |
| GO Biological Process | Regulation of myeloid leukocyte differentiation |
| GO Biological Process | Regulation of mononuclear cell migration |
| GO Biological Process | Positive regulation of type 2 immune response |
| GO Biological Process | Positive regulation of nitric-oxide synthase biosynthetic process |
| GO Biological Process | Positive regulation of stress-activated MAPK cascade |
| GO Biological Process | Regulation of T cell proliferation |
| GO Biological Process | Regulation of leukocyte chemotaxis |
| GO Biological Process | Acute inflammatory response |
| GO Biological Process | Negative regulation of immune system process |
| GO Biological Process | Chemokine-mediated signaling pathway |
| GO Biological Process | Positive regulation of B cell proliferation |
| GO Biological Process | Positive regulation of receptor signaling pathway via JAK-STAT |
| GO Biological Process | Hemopoiesis |
| GO Biological Process | Activation of cysteine-type endopeptidase activity |
| GO Biological Process | Negative regulation of production of molecular mediator of immune response |
| GO Biological Process | Negative regulation of I-kappaB kinase/NF-kappaB signaling |
| GO Biological Process | Regulation of biological quality |
| GO Biological Process | Positive regulation of transport |
| GO Biological Process | Regulation of interleukin-13 production |
| GO Biological Process | Regulation of cellular catabolic process |
| GO Biological Process | Negative regulation of interleukin-6 production |
| GO Biological Process | Intracellular signal transduction |
| GO Biological Process | Regulation of transcription, DNA-templated |
| GO Biological Process | Positive regulation of nucleobase-containing compound metabolic process |
| GO Biological Process | Positive regulation of ion transport |
| GO Biological Process | Regulation of angiogenesis |
| GO Biological Process | Negative regulation of inflammatory response |
| GO Biological Process | Positive regulation of ERK1 and ERK2 cascade |
| GO Biological Process | Regulation of myeloid cell differentiation |
| GO Biological Process | Positive regulation of interleukin-23 production |
| GO Biological Process | Positive regulation of cytokine production involved in inflammatory response |
| GO Biological Process | Positive regulation of JNK cascade |
| GO Biological Process | Regulation of localization |
| GO Biological Process | Defense response to Gram-negative bacterium |
| GO Biological Process | MAPK cascade |
| GO Biological Process | Negative regulation of response to external stimulus |
| GO Biological Process | Positive regulation of leukocyte chemotaxis |
| GO Biological Process | Regulation of natural killer cell chemotaxis |
| GO Biological Process | Regulation of catabolic process |
| GO Biological Process | Regulation of NIK/NF-kappaB signaling |
| GO Biological Process | Positive regulation of macrophage activation |
| GO Biological Process | Regulation of lymphocyte chemotaxis |
| GO Biological Process | Regulation of nucleobase-containing compound metabolic process |
| GO Biological Process | Cellular response to organic cyclic compound |
| GO Biological Process | Regulation of epithelial cell apoptotic process |
| GO Biological Process | Negative regulation of intracellular signal transduction |
| GO Biological Process | Positive regulation of peptidyl-serine phosphorylation |
| GO Biological Process | Regulation of interleukin-12 production |
| GO Biological Process | Regulation of chronic inflammatory response |
| GO Biological Process | Natural killer cell activation |
| GO Biological Process | Regulation of protein secretion |
| GO Biological Process | Positive regulation of NIK/NF-kappaB signaling |
| GO Biological Process | Mononuclear cell migration |
| GO Biological Process | Regulation of T cell differentiation |
| GO Biological Process | Response to nitrogen compound |
| GO Biological Process | Regulation of signaling receptor activity |
| GO Biological Process | Granulocyte activation |
| GO Biological Process | Regulation of lymphocyte migration |
| GO Biological Process | Positive regulation of transcription, DNA-templated |
| GO Biological Process | Regulation of osteoclast differentiation |
| GO Biological Process | Regulation of autophagy |
| GO Biological Process | Negative regulation of immune response |
| GO Biological Process | Negative regulation of cytokine production involved in immune response |
| GO Biological Process | Negative regulation of interleukin-1 production |
| GO Biological Process | Type 2 immune response |
| GO Biological Process | Positive regulation of response to biotic stimulus |
| GO Biological Process | B cell activation |
| GO Biological Process | Positive regulation of transcription by RNA polymerase II |
| GO Biological Process | Response to ethanol |
| GO Biological Process | Adaptive immune response |
| GO Biological Process | Positive regulation of protein-containing complex assembly |
| GO Biological Process | Cell killing |
| GO Biological Process | Positive regulation of lymphocyte differentiation |
| GO Biological Process | Inflammatory response to antigenic stimulus |
| GO Biological Process | Regulation of ion transport |
| GO Biological Process | Positive regulation of interleukin-13 production |
| GO Biological Process | Multicellular organismal homeostasis |
| GO Biological Process | Myeloid leukocyte differentiation |
| GO Biological Process | Protein processing |
| GO Biological Process | Acute-phase response |
| GO Biological Process | Type I interferon signaling pathway |
| GO Biological Process | Positive regulation of kinase activity |
| GO Biological Process | Monocyte chemotaxis |
| GO Biological Process | Positive regulation of lymphocyte migration |
| GO Biological Process | Negative regulation of cell death |
| GO Biological Process | Positive regulation of cellular component biogenesis |
| GO Biological Process | Positive regulation of innate immune response |
| GO Biological Process | Regulation of secretion |
| GO Biological Process | Leukocyte proliferation |
| GO Biological Process | Eosinophil chemotaxis |
| GO Biological Process | Positive regulation of calcidiol 1-monooxygenase activity |
| GO Biological Process | Positive regulation of neuron death |
| GO Biological Process | Response to endogenous stimulus |
| GO Biological Process | Positive regulation of cellular component organization |
| GO Biological Process | Cytokine production |
| GO Biological Process | Regulation of extrinsic apoptotic signaling pathway |
| GO Biological Process | Regulation of cellular respiration |
| GO Biological Process | Positive regulation of cellular catabolic process |
| GO Biological Process | Regulation of protein-containing complex assembly |
| GO Biological Process | Negative regulation of programmed cell death |
| GO Biological Process | Cellular process |
| GO Biological Process | Positive regulation of protein kinase activity |
| GO Biological Process | Regulation of acute inflammatory response |
| GO Biological Process | Regulation of kinase activity |
| GO Biological Process | Negative regulation of extrinsic apoptotic signaling pathway |
| GO Biological Process | Receptor signaling pathway via JAK-STAT |
| GO Biological Process | Lymphocyte chemotaxis |
| GO Biological Process | Regulation of endothelial cell apoptotic process |
| GO Biological Process | Regulation of NLRP3 inflammasome complex assembly |
| GO Biological Process | Regulation of innate immune response |
| GO Biological Process | Regulation of protein kinase activity |
| GO Biological Process | System development |
| GO Biological Process | Positive regulation of myeloid cell differentiation |
| GO Biological Process | B cell proliferation |
| GO Biological Process | Protein kinase B signaling |
| GO Biological Process | Positive regulation of peptidyl-serine phosphorylation of STAT protein |
| GO Biological Process | Positive regulation of angiogenesis |
| GO Biological Process | Regulation of protein kinase B signaling |
| GO Biological Process | Regulation of tumor necrosis factor-mediated signaling pathway |
| GO Biological Process | Regulation of CD4-positive, alpha-beta T cell differentiation |
| GO Biological Process | Regulation of type I interferon production |
| GO Biological Process | Regulation of cellular component organization |
| GO Biological Process | Antiviral innate immune response |
| GO Biological Process | Regulation of secretion by cell |
| GO Biological Process | Response to alcohol |
| GO Biological Process | Negative regulation of leukocyte differentiation |
| GO Biological Process | Negative regulation of adaptive immune response based on somatic recombination of immune receptors built from immunoglobulin superfamily domains |
| GO Biological Process | Regulation of cellular component biogenesis |
| GO Biological Process | Animal organ development |
| GO Biological Process | Biological process involved in interaction with symbiont |
| GO Biological Process | Positive regulation of myeloid leukocyte differentiation |
| GO Biological Process | Biological process involved in symbiotic interaction |
| GO Biological Process | Lymphocyte activation |
| GO Biological Process | Regulation of nitric oxide biosynthetic process |
| GO Biological Process | Regulation of heterotypic cell-cell adhesion |
| GO Biological Process | Myeloid cell differentiation |
| GO Biological Process | Cell population proliferation |
| GO Biological Process | Regulation of cellular response to stress |
| GO Biological Process | Myeloid cell activation involved in immune response |
| GO Biological Process | Regulation of membrane protein ectodomain proteolysis |
| GO Biological Process | Positive regulation of macrophage cytokine production |
| GO Biological Process | Positive regulation of T cell differentiation |
| GO Biological Process | Regulation of apoptotic signaling pathway |
| GO Biological Process | Positive regulation of response to cytokine stimulus |
| GO Biological Process | Osmosensory signaling pathway |
| GO Biological Process | Positive regulation of natural killer cell chemotaxis |
| GO Biological Process | Activation of innate immune response |
| GO Biological Process | Positive regulation of interleukin-17 production |
| GO Biological Process | Negative regulation of signaling receptor activity |
| GO Biological Process | Protein homooligomerization |
| GO Biological Process | Lymphocyte proliferation |
| GO Biological Process | Negative regulation of apoptotic process |
| GO Biological Process | I-KappaB kinase/NF-kappaB signaling |
| GO Biological Process | Macrophage differentiation |
| GO Biological Process | Neutrophil activation |
| GO Biological Process | Regulation of protein serine/threonine kinase activity |
| GO Biological Process | Wound healing involved in inflammatory response |
| GO Biological Process | Vascular endothelial growth factor production |
| GO Biological Process | Liver regeneration |
| GO Biological Process | Lymphocyte activation involved in immune response |
| GO Biological Process | B cell differentiation |
| GO Biological Process | Cell motility |
| GO Biological Process | Negative regulation of ERK1 and ERK2 cascade |
| GO Biological Process | Regulation of T-helper 1 type immune response |
| GO Biological Process | Positive regulation of interleukin-18 production |
| GO Biological Process | Homeostatic process |
| GO Biological Process | Regulation of MHC class I biosynthetic process |
| GO Biological Process | Positive regulation of calcium ion transport |
| GO Biological Process | Positive regulation of T-helper 1 cell cytokine production |
| GO Biological Process | Regulation of interleukin-1-mediated signaling pathway |
| GO Biological Process | Negative regulation of cytokine-mediated signaling pathway |
| GO Biological Process | T Cell activation involved in immune response |
| GO Biological Process | Intrinsic apoptotic signaling pathway in response to DNA damage |
| GO Biological Process | Positive regulation of phagocytosis |
| GO Biological Process | Regulation of endothelial cell proliferation |
| GO Biological Process | Positive regulation of phosphatidylinositol 3-kinase signaling |
| GO Biological Process | Self proteolysis |
| GO Biological Process | Positive regulation of T-helper 2 cell cytokine production |
| GO Biological Process | Response to organonitrogen compound |
| GO Biological Process | Astrocyte development |
| GO Biological Process | Regulation of sensory perception of pain |
| GO Biological Process | Extrinsic apoptotic signaling pathway in absence of ligand |
| GO Biological Process | Negative regulation of extrinsic apoptotic signaling pathway in absence of ligand |
| GO Biological Process | Positive regulation of autophagy |
| GO Biological Process | Positive regulation of small molecule metabolic process |
| GO Biological Process | Positive regulation of protein serine/threonine kinase activity |
| GO Biological Process | Mononuclear cell differentiation |
| GO Biological Process | Regulation of epithelial cell differentiation |
| GO Biological Process | Negative regulation of apoptotic signaling pathway |
| GO Biological Process | Regulation of insulin secretion |
| GO Biological Process | Anatomical structure development |
| GO Biological Process | Positive regulation of podosome assembly |
| GO Biological Process | Regulation of T-helper cell differentiation |
| GO Biological Process | Positive regulation of interleukin-12 production |
| GO Biological Process | Regulation of transmembrane transport |
| GO Biological Process | Gliogenesis |
| GO Biological Process | Positive regulation of nitric oxide biosynthetic process |
| GO Biological Process | Negative regulation of interferon-gamma production |
| GO Biological Process | Intrinsic apoptotic signaling pathway |
| GO Biological Process | Response to corticosteroid |
| GO Biological Process | Macrophage chemotaxis |
| GO Biological Process | Cellular response to interleukin-17 |
| GO Biological Process | Regulation of lipid localization |
| GO Biological Process | Regulation of defense response to virus by host |
| GO Biological Process | Negative regulation of MAPK cascade |
| GO Biological Process | Cellular response to stress |
| GO Biological Process | Negative regulation of T cell differentiation |
| GO Biological Process | Positive regulation of cellular respiration |
| GO Biological Process | Regulation of neutrophil migration |
| GO Biological Process | Negative regulation of epithelial cell differentiation |
| GO Biological Process | Regulation of oxidoreductase activity |
| GO Biological Process | Reactive oxygen species metabolic process |
| GO Biological Process | Regulation of calcium ion transport |
| GO Biological Process | Positive regulation of granulocyte macrophage colony-stimulating factor production |
| GO Biological Process | Positive regulation of heterotypic cell-cell adhesion |
| GO Biological Process | Negative regulation by host of viral transcription |
| GO Biological Process | Cell-cell signaling |
| GO Biological Process | Regulation of lipid storage |
| GO Biological Process | Regulation of anatomical structure morphogenesis |
| GO Biological Process | Regulation of monooxygenase activity |
| GO Biological Process | Epithelial cell apoptotic process |
| GO Biological Process | Lymphocyte differentiation |
| GO Biological Process | Negative regulation of smooth muscle cell proliferation |
| GO Biological Process | Astrocyte activation |
| GO Biological Process | Positive regulation of membrane protein ectodomain proteolysis |
| GO Biological Process | Positive regulation of protein kinase B signaling |
| GO Biological Process | Negative regulation of cell-cell adhesion |
| GO Biological Process | Positive regulation of MAP kinase activity |
| GO Biological Process | Positive regulation of glial cell proliferation |
| GO Biological Process | Regulation of chemokine (C-X-C motif) ligand 2 production |
| GO Biological Process | Negative regulation of leukocyte activation |
| GO Biological Process | Positive regulation of cytokine-mediated signaling pathway |
| GO Biological Process | JNK cascade |
| GO Biological Process | Regulation of metal ion transport |
| GO Biological Process | Myeloid leukocyte mediated immunity |
| GO Biological Process | Response to inorganic substance |
| GO Biological Process | T Cell activation |
| GO Biological Process | Regulation of amyloid-beta clearance |
| GO Biological Process | Negative regulation of epithelial cell apoptotic process |
| GO Biological Process | Toll-like receptor signaling pathway |
| GO Biological Process | Maintenance of gastrointestinal epithelium |
| GO Biological Process | Regulation of interleukin-5 production |
| GO Biological Process | Negative regulation of tumor necrosis factor production |
| GO Biological Process | Positive regulation of DNA metabolic process |
| GO Biological Process | Interleukin-1-mediated signaling pathway |
| GO Biological Process | Negative regulation of phosphate metabolic process |
| GO Biological Process | MyD88-Dependent toll-like receptor signaling pathway |
| GO Biological Process | Regulation of immunoglobulin mediated immune response |
| GO Biological Process | Negative regulation of lipid storage |
| GO Biological Process | Positive regulation of type I interferon production |
| GO Biological Process | Positive regulation of vitamin D biosynthetic process |
| GO Biological Process | Pyroptosome complex assembly |
| GO Biological Process | Positive regulation of humoral immune response |
| GO Biological Process | Positive regulation of steroid biosynthetic process |
| GO Biological Process | Regulation of neuron apoptotic process |
| GO Biological Process | Negative regulation of T cell activation |
| GO Biological Process | Regulation of transcription by RNA polymerase II |
| GO Biological Process | Positive regulation of gliogenesis |
| GO Biological Process | Angiogenesis |
| GO Biological Process | Negative regulation of neuron death |
| GO Biological Process | Negative regulation of protein phosphorylation |
| GO Biological Process | Negative regulation of protein metabolic process |
| GO Biological Process | Multicellular organismal process |
| GO Biological Process | Negative regulation of chemokine production |
| GO Biological Process | Positive regulation of reactive oxygen species metabolic process |
| GO Biological Process | Regulation of steroid biosynthetic process |
| GO Biological Process | Regulation of viral life cycle |
| GO Biological Process | Myeloid dendritic cell activation |
| GO Biological Process | Neutrophil mediated immunity |
| GO Biological Process | Response to toxic substance |
| GO Biological Process | Killing of cells of another organism |
| GO Biological Process | Wound healing |
| GO Biological Process | Fever generation |
| GO Biological Process | Chronic inflammatory response to antigenic stimulus |
| GO Biological Process | Regulation of chronic inflammatory response to antigenic stimulus |
| GO Biological Process | Response to fungus |
| GO Biological Process | interleukin-33-mediated signaling pathway |
| GO Biological Process | Positive regulation of plasma cell differentiation |
| GO Biological Process | Regulation of oxidative stress-induced cell death |
| GO Biological Process | Negative regulation of complement-dependent cytotoxicity |
| GO Biological Process | Regulation of reactive oxygen species metabolic process |
| GO Biological Process | Positive regulation of interleukin-18-mediated signaling pathway |
| GO Biological Process | Natural killer cell activation involved in immune response |
| GO Biological Process | Regulation of nervous system process |
| GO Biological Process | Regulation of organic acid transport |
| GO Biological Process | Positive regulation of osteoclast differentiation |
| GO Biological Process | Response to glucocorticoid |
| GO Biological Process | Tissue homeostasis |
| GO Biological Process | Positive regulation of acute inflammatory response |
| GO Biological Process | Positive regulation of interleukin-4 production |
| GO Biological Process | Positive regulation of activated T cell proliferation |
| GO Biological Process | Regulation of myoblast differentiation |
| GO Biological Process | Negative regu4lation of myoblast differentiation |
| GO Biological Process | Modulation by host of symbiont process |
| GO Biological Process | Response to osmotic stress |
| GO Biological Process | Positive regulation of vascular endothelial growth factor production |
| GO Biological Process | Negative regulation of interleukin-1 beta production |
| GO Biological Process | Killing by host of symbiont cells |
| GO Biological Process | Smooth muscle adaptation |
| GO Biological Process | Cellular homeostasis |
| GO Biological Process | Sequestering of triglyceride |
| GO Biological Process | NLRP3 inflammasome complex assembly |
| GO Biological Process | Negative regulation of T-helper 2 cell cytokine production |
| GO Biological Process | Regulation of macrophage derived foam cell differentiation |
| GO Biological Process | Regulation of interferon-alpha production |
| GO Biological Process | Regulation of oxidative stress-induced neuron death |
| GO Biological Process | Positive regulation of leukocyte apoptotic process |
| GO Biological Process | Negative regulation of endothelial cell apoptotic process |
| GO Biological Process | Regulation of ion transmembrane transport |
| GO Biological Process | Regulation of mast cell degranulation |
| GO Biological Process | Regulation of viral genome replication |
| GO Biological Process | Regulation of B cell differentiation |
| GO Biological Process | Positive regulation of lipid biosynthetic process |
| GO Biological Process | Negative regulation of protein modification process |
| GO Biological Process | Positive regulation of CD4-positive, alpha-beta T cell differentiation |
| GO Biological Process | Negative regulation of osteoclast differentiation |
| GO Biological Process | Negative regulation of chronic inflammatory response |
| GO Biological Process | Cell differentiation |
| GO Biological Process | Intestinal epithelial structure maintenance |
| GO Biological Process | Negative regulation of interleukin-1-mediated signaling pathway |
| GO Biological Process | Positive regulation of defense response to virus by host |
| GO Biological Process | Modulation of process of another organism |
| GO Biological Process | Cellular response to chemical stress |
| GO Biological Process | Positive regulation of cation transmembrane transport |
| GO Biological Process | Regulation of endothelial cell differentiation |
| GO Biological Process | Regulation of lipid biosynthetic process |
| GO Biological Process | T Cell migration |
| GO Biological Process | Positive regulation of neutrophil migration |
| GO Biological Process | Positive regulation of T cell migration |
| GO Biological Process | Regulation of hormone levels |
| GO Biological Process | Regulation of anion transport |
| GO Biological Process | Positive regulation of nervous system development |
| GO Biological Process | Response to hypoxia |
| GO Biological Process | Negative regulation of viral process |
| GO Biological Process | Regulation of leukocyte apoptotic process |
| GO Biological Process | Detection of biotic stimulus |
| GO Biological Process | Hydrogen peroxide metabolic process |
| GO Biological Process | Peptidyl-cysteine S-nitrosylation |
| GO Biological Process | Positive regulation of immature T cell proliferation in thymus |
| GO Biological Process | Positive regulation of MHC class I biosynthetic process |
| GO Biological Process | Negative regulation of nitrogen compound metabolic process |
| GO Biological Process | Positive regulation of killing of cells of another organism |
| GO Biological Process | Positive regulation of secretion by cell |
| GO Biological Process | Positive regulation of NMDA glutamate receptor activity |
| GO Biological Process | Regulation of chemokine (C-X-C motif) ligand 1 production |
| GO Biological Process | Regulation of isotype switching |
| GO Biological Process | Regulation of DNA metabolic process |
| GO Biological Process | Regulation of protein localization |
| GO Biological Process | Negative regulation of leukocyte proliferation |
| GO Biological Process | Regulation of vesicle-mediated transport |
| GO Biological Process | Regulation of nitric-oxide synthase activity |
| GO Biological Process | Cellular response to amyloid-beta |
| GO Biological Process | Regulation of cell killing |
| GO Biological Process | Positive regulation of fever generation |
| GO Biological Process | Astrocyte cell migration |
| GO Biological Process | Positive regulation of T-helper 2 cell differentiation |
| GO Biological Process | Negative regulation of NLRP3 inflammasome complex assembly |
| GO Biological Process | Organ or tissue specific immune response |
| GO Biological Process | Superoxide metabolic process |
| GO Biological Process | Response to xenobiotic stimulus |
| GO Biological Process | Regulation of inflammatory response to antigenic stimulus |
| GO Biological Process | Positive regulation of immunoglobulin mediated immune response |
| GO Biological Process | Positive regulation of calcium-mediated signaling |
| GO Biological Process | Positive regulation of epithelial cell apoptotic process |
| GO Biological Process | Positive regulation of protein transport |
| GO Biological Process | Negative regulation of endothelial cell proliferation |
| GO Biological Process | Positive regulation of cell development |
| GO Biological Process | Negative regulation of cellular metabolic process |
| GO Biological Process | Organonitrogen compound metabolic process |
| GO Biological Process | Proteolysis |
| GO Biological Process | Regulation of cell adhesion molecule production |
| GO Biological Process | Positive regulation of oxidative stress-induced neuron death |
| GO Biological Process | Response to peptide |
| GO Biological Process | Regulation of cell junction assembly |
| GO Biological Process | Positive regulation of mitotic nuclear division |
| GO Biological Process | Antimicrobial humoral immune response mediated by antimicrobial peptide |
| GO Biological Process | Positive regulation of organic acid transport |
| GO Biological Process | Positive regulation of signaling receptor activity |
| GO Biological Process | Neutrophil-mediated killing of bacterium |
| GO Biological Process | Negative regulation of amyloid-beta clearance |
| GO Biological Process | Cellular response to heat |
| GO Biological Process | Response to nicotine |
| GO Biological Process | Positive regulation of purine nucleotide metabolic process |
| GO Biological Process | Leukocyte mediated immunity |
| GO Biological Process | Cellular response to endogenous stimulus |
| GO Biological Process | Negative regulation of leukocyte migration |
| GO Biological Process | Icosanoid biosynthetic process |
| GO Biological Process | Response to hormone |
| GO Biological Process | Negative regulation of catabolic process |
| GO Biological Process | Response to exogenous dsRNA |
| GO Biological Process | Positive regulation of anion transport |
| GO Biological Process | Regulation of prostaglandin biosynthetic process |
| GO Biological Process | Response to cobalt ion |
| GO Biological Process | Negative regulation of heterotypic cell-cell adhesion |
| GO Biological Process | Positive regulation of isotype switching to IgG isotypes |
| GO Biological Process | Regulation of gap junction assembly |
| GO Biological Process | Positive regulation of chemokine (C-X-C motif) ligand 2 production |
| GO Biological Process | Negative regulation of fat cell differentiation |
| GO Biological Process | Regulation of granulocyte chemotaxis |
| GO Biological Process | Positive regulation of interleukin-5 production |
| GO Biological Process | Positive regulation of tissue remodeling |
| GO Biological Process | Endothelial cell apoptotic process |
| GO Biological Process | Cellular response to oxidized low-density lipoprotein particle stimulus |
| GO Biological Process | Negative regulation of T-helper 17 cell differentiation |
| GO Biological Process | Positive regulation of neuron apoptotic process |
| GO Biological Process | Positive regulation of neurogenesis |
| GO Biological Process | B cell activation involved in immune response |
| GO Biological Process | Regulation of epithelial cell migration |
| GO Biological Process | Positive regulation of apoptotic signaling pathway |
| GO Biological Process | Leukocyte mediated cytotoxicity |
| GO Biological Process | Leukocyte cell-cell adhesion |
| GO Biological Process | Tumor necrosis factor-mediated signaling pathway |
| GO Biological Process | Negative regulation of viral genome replication |
| GO Biological Process | Regulation of DNA recombination |
| GO Biological Process | I-KappaB phosphorylation |
| GO Biological Process | Positive regulation of NLRP3 inflammasome complex assembly |
| GO Biological Process | Positive regulation of tumor necrosis factor-mediated signaling pathway |
| GO Biological Process | Response to extracellular stimulus |
| GO Biological Process | Circulatory system process |
| GO Biological Process | Regulation of mitotic cell cycle |
| GO Biological Process | Regulation of interferon-beta production |
| GO Biological Process | Response to metal ion |
| GO Biological Process | Negative regulation of acute inflammatory response |
| GO Biological Process | Response to peptidoglycan |
| GO Biological Process | Protein metabolic process |
| GO Biological Process | Regulation of cell development |
| GO Biological Process | Regulation of protein catabolic process |
| GO Biological Process | Positive regulation of organelle organization |
| GO Biological Process | Response to oxidative stress |
| GO Biological Process | Regulation of vascular associated smooth muscle cell proliferation |
| GO Biological Process | Positive regulation of prostaglandin secretion |
| GO Biological Process | Secretion |
| GO Biological Process | Regulation of lymphocyte apoptotic process |
| GO Biological Process | Regulation of cation transmembrane transport |
| GO Biological Process | Regulation of cold-induced thermogenesis |
| GO Biological Process | Response to nutrient |
| GO Biological Process | Negative regulation of interleukin-17 production |
| GO Biological Process | Positive regulation of mast cell degranulation |
| GO Biological Process | Positive regulation of calcium ion import |
| GO Biological Process | Reactive oxygen species biosynthetic process |
| GO Biological Process | Regulation of muscle cell apoptotic process |
| GO Biological Process | Negative regulation of leukocyte mediated immunity |
| GO Biological Process | Response to activity |
| GO Biological Process | Regulation of dendritic cell cytokine production |
| GO Biological Process | T Cell chemotaxis |
| GO Biological Process | Regulation of establishment of endothelial barrier |
| GO Biological Process | Negative regulation of miRNA maturation |
| GO Biological Process | Regulation of T cell chemotaxis |
| GO Biological Process | Regulation of amino acid import across plasma membrane |
| GO Biological Process | Positive regulation of homotypic cell-cell adhesion |
| GO Biological Process | Blood circulation |
| GO Biological Process | Regulation of macroautophagy |
| GO Biological Process | Positive regulation of DNA recombination |
| GO Biological Process | Positive regulation of macrophage derived foam cell differentiation |
| GO Biological Process | Negative regulation of glucose transmembrane transport |
| GO Biological Process | Negative regulation of interleukin-12 production |
| GO Biological Process | Ectopic germ cell programmed cell death |
| GO Biological Process | Regulation of microglial cell activation |
| GO Biological Process | Positive regulation of macroautophagy |
| GO Biological Process | Regulation of transmembrane transporter activity |
| GO Biological Process | Circulatory system development |
| GO Biological Process | Regulation of tissue remodeling |
| GO Biological Process | Regulation of system process |
| GO Biological Process | Macrophage activation involved in immune response |
| GO Biological Process | Negative regulation of interleukin-8 production |
| GO Biological Process | Positive regulation of lymphocyte apoptotic process |
| GO Biological Process | Response to reactive oxygen species |
| GO Biological Process | Adaptive immune response based on somatic recombination of immune receptors built from immunoglobulin superfamily domains |
| GO Biological Process | Negative regulation of cell migration |
| GO Biological Process | Negative regulation of nervous system process |
| GO Biological Process | Macromolecule metabolic process |
| GO Biological Process | Protein-containing complex assembly |
| GO Biological Process | Necroptotic process |
| GO Biological Process | Detection of other organisms |
| GO Biological Process | Regulation of bicellular tight junction assembly |
| GO Biological Process | Response to temperature stimulus |
| GO Biological Process | Positive regulation of cellular amide metabolic process |
| GO Biological Process | Nitric oxide biosynthetic process |
| GO Biological Process | Myeloid dendritic cell differentiation |
| GO Biological Process | Regulation of myoblast fusion |
| GO Biological Process | Positive regulation of amyloid-beta formation |
| GO Biological Process | Cellular response to fibroblast growth factor stimulus |
| GO Biological Process | Regulation of B cell apoptotic process |
| GO Biological Process | Negative regulation of ATP-dependent activity |
| GO Biological Process | T-Helper 1 type immune response |
| GO Biological Process | Superoxide anion generation |
| GO Biological Process | Negative regulation of cell development |
| GO Biological Process | Negative regulation of catalytic activity |
| GO Biological Process | Embryonic placenta development |
| GO Biological Process | Regulation of smooth muscle cell apoptotic process |
| GO Biological Process | Cellular response to interferon-beta |
| GO Biological Process | Positive regulation of regulatory T cell differentiation |
| GO Biological Process | Negative regulation of heart contraction |
| GO Biological Process | Positive regulation of exocytosis |
| GO Biological Process | Positive regulation of monocyte chemotaxis |
| GO Biological Process | Positive regulation of leukocyte adhesion to vascular endothelial cell |
| GO Biological Process | Cell adhesion |
| GO Biological Process | Positive regulation of lipid transport |
| GO Biological Process | Granulocyte differentiation |
| GO Biological Process | Negative regulation of cytokine production involved in inflammatory response |
| GO Biological Process | Regulation of blood vessel endothelial cell migration |
| GO Biological Process | Neuron apoptotic process |
| GO Biological Process | Regulation of blood pressure |
| GO Biological Process | Response to nutrient levels |
| GO Biological Process | Positive regulation of interferon-alpha production |
| GO Biological Process | Dendritic cell migration |
| GO Biological Process | Hydrogen peroxide catabolic process |
| GO Biological Process | Negative regulation of vascular associated smooth muscle cell proliferation |
| GO Biological Process | Negative regulation of mononuclear cell proliferation |
| GO Cellular Component | Inflammasome complex |
| GO Cellular Component | Extracellular region |
| GO Cellular Component | Extracellular space |
| GO Cellular Component | NLRP3 inflammasome complex |
| GO Cellular Component | NLRP1 inflammasome complex |
| GO Cellular Component | AIM2 inflammasome complex |
| GO Cellular Component | IPAF inflammasome complex |
| GO Cellular Component | Caspase complex |
| GO Cellular Component | NLRP6 inflammasome complex |
| GO Molecular Function | Cytokine receptor binding |
| GO Molecular Function | Cytokine activity |
| GO Molecular Function | Signaling receptor regulator activity |
| GO Molecular Function | Signaling receptor binding |
| GO Molecular Function | Molecular function regulator activity |
| GO Molecular Function | Growth factor receptor binding |
| GO Molecular Function | Protein binding |
| GO Molecular Function | Interleukin-1 receptor binding |
| GO Molecular Function | Cysteine-type endopeptidase activity involved in apoptotic process |
| GO Molecular Function | Binding |
| GO Molecular Function | Chemokine activity |
| GO Molecular Function | Cysteine-type endopeptidase activity involved in apoptotic signaling pathway |
| GO Molecular Function | Identical protein binding |
| GO Molecular Function | Cysteine-type endopeptidase activator activity involved in apoptotic process |
| GO Molecular Function | Peptidase activator activity |
| GO Molecular Function | Growth factor activity |
| GO Molecular Function | Enzyme activator activity |
| GO Molecular Function | CCR1 chemokine receptor binding |
| GO Molecular Function | Pattern recognition receptor activity |
| GO Molecular Function | CCR5 chemokine receptor binding |
| GO Molecular Function | G-Protein-coupled receptor binding |
| GO Molecular Function | Interleukin-1 binding |
| GO Molecular Function | Heme binding |
| GO Molecular Function | Peptidase regulator activity |
| GO Molecular Function | Endopeptidase activity |
| GO Molecular Function | Protein domain specific binding |
| GO Molecular Function | Endopeptidase activator activity |
| GO Molecular Function | CCR chemokine receptor binding |
| GO Molecular Function | NAD(P)+ nucleosidase activity |
| GO Molecular Function | CARD domain binding |
| GO Molecular Function | NAD+ nucleotidase, cyclic ADP-ribose generating |
| GO Molecular Function | Type I interferon receptor binding |
| GO Molecular Function | CXCR chemokine receptor binding |
| GO Molecular Function | Cysteine-type endopeptidase activator activity |
| GO Molecular Function | Interleukin-1 receptor antagonist activity |
| GO Molecular Function | Enzyme regulator activity |
| GO Molecular Function | Protein self-association |
| GO Molecular Function | Protein dimerization activity |
| GO Molecular Function | Receptor antagonist activity |
| GO Molecular Function | Interleukin-6 receptor binding |
| GO Molecular Function | Flavin adenine dinucleotide binding |
| GO Molecular Function | Chemoattractant activity |
| GO Molecular Function | Interleukin-1 receptor activity |
| GO Molecular Function | Protein homodimerization activity |
| GO Molecular Function | Cysteine-type endopeptidase activity involved in execution phase of apoptosis |
| GO Molecular Function | Tumor necrosis factor receptor superfamily binding |

**Table S18. KEGG Enrichment Entry.**

| **Category** | **Description** |
| --- | --- |
| KEGG Pathways | NOD-like receptor signaling pathway |
| KEGG Pathways | Cytokine-cytokine receptor interaction |
| KEGG Pathways | Influenza A |
| KEGG Pathways | Yersinia infection |
| KEGG Pathways | Chagas disease |
| KEGG Pathways | Toll-like receptor signaling pathway |
| KEGG Pathways | IL-17 signaling pathway |
| KEGG Pathways | Tuberculosis |
| KEGG Pathways | Salmonella infection |
| KEGG Pathways | Legionellosis |
| KEGG Pathways | Rheumatoid arthritis |
| KEGG Pathways | Inflammatory bowel disease |
| KEGG Pathways | Cytosolic DNA-sensing pathway |
| KEGG Pathways | Malaria |
| KEGG Pathways | Pathogenic Escherichia coli infection |
| KEGG Pathways | Necroptosis |
| KEGG Pathways | Viral protein interaction with cytokine and cytokine receptor |
| KEGG Pathways | Shigellosis |
| KEGG Pathways | Pertussis |
| KEGG Pathways | Measles |
| KEGG Pathways | JAK-STAT signaling pathway |
| KEGG Pathways | Human cytomegalovirus infection |
| KEGG Pathways | Amoebiasis |
| KEGG Pathways | Leishmaniasis |
| KEGG Pathways | African trypanosomiasis |
| KEGG Pathways | TNF signaling pathway |
| KEGG Pathways | Hepatitis B |
| KEGG Pathways | Hematopoietic cell lineage |
| KEGG Pathways | C-Type lectin receptor signaling pathway |
| KEGG Pathways | Herpes simplex virus 1 infection |
| KEGG Pathways | Kaposi sarcoma-associated herpesvirus infection |
| KEGG Pathways | Epstein-Barr virus infection |
| KEGG Pathways | Pathways in cancer |
| KEGG Pathways | AGE-RAGE signaling pathway in diabetic complications |
| KEGG Pathways | NF-Kappa B signaling pathway |
| KEGG Pathways | Toxoplasmosis |
| KEGG Pathways | Hepatitis C |
| KEGG Pathways | Allograft rejection |
| KEGG Pathways | Graft-versus-host disease |
| KEGG Pathways | RIG-I-like receptor signaling pathway |
| KEGG Pathways | Fluid shear stress and atherosclerosis |
| KEGG Pathways | Autoimmune thyroid disease |
| KEGG Pathways | Th17 cell differentiation |
| KEGG Pathways | T Cell receptor signaling pathway |
| KEGG Pathways | HIF-1 signaling pathway |
| KEGG Pathways | Asthma |
| KEGG Pathways | Osteoclast differentiation |
| KEGG Pathways | Natural killer cell mediated cytotoxicity |
| KEGG Pathways | PI3K-Akt signaling pathway |
| KEGG Pathways | Alzheimer disease |
| KEGG Pathways | Type I diabetes mellitus |
| KEGG Pathways | Human immunodeficiency virus 1 infection |
| KEGG Pathways | Intestinal immune network for IgA production |
| KEGG Pathways | Chemokine signaling pathway |
| KEGG Pathways | Fc epsilon RI signaling pathway |
| KEGG Pathways | Non-alcoholic fatty liver disease |
| KEGG Pathways | Th1 and Th2 cell differentiation |
| KEGG Pathways | Prion disease |
| KEGG Pathways | Antifolate resistance |
| KEGG Pathways | PD-L1 expression and PD-1 checkpoint pathway in cancer |
| KEGG Pathways | Human papillomavirus infection |
| KEGG Pathways | Human T cell leukemia virus 1 infection |
| KEGG Pathways | Viral myocarditis |
| KEGG Pathways | Apoptosis |
| KEGG Pathways | Phagosome |
| KEGG Pathways | Epithelial cell signaling in Helicobacter pylori infection |
| KEGG Pathways | Cellular senescence |
| KEGG Pathways | MAPK signaling pathway |
| KEGG Pathways | Hypertrophic cardiomyopathy |
| KEGG Pathways | Systemic lupus erythematosus |
| KEGG Pathways | Apoptosis - multiple species |
| KEGG Pathways | Amyotrophic lateral sclerosis |
| KEGG Pathways | FoxO signaling pathway |
| KEGG Pathways | Ferroptosis |

**Table S19. Binding Energies and RMSD of the 13 Compounds into the Active Pocket Site of the SIRT1 Catalytic Domain (PDB: 4i5i).**

| **NO** | **S (kcal/mol)** | **RMSD_refine** |
| --- | --- | --- |
| 1 | -5.27458 | 1.38488388 |
| 2 | -5.97551918 | 1.42401814 |
| 3 | -5.8754549 | 1.61728275 |
| 4 | -5.94139242 | 1.05822444 |
| 5 | -6.3869772 | 1.18472898 |
| 6 | -6.42374039 | 1.21052182 |
| 7 | -6.38610697 | 1.61750984 |
| 8 | -6.50890446 | 1.33429134 |
| 9 | -6.97040892 | 0.929638445 |
| 10 | -6.63670635 | 1.18752587 |
| 11 | -7.7294302 | 1.09942734 |
| 12 | -7.98590326 | 0.726437182 |
| 13 | -7.38594723 | 1.07457232 |
| Co-ligand | -6.82321644 | 0.798828483 |
| Standard control (resveratrol) | -7.12138753 | 0.824170619 |
