# Supplementary material for: Emphasizing the role of oxidative stress and Sirt-1/Nrf2 and TLR-4/NF-κB in Tamarix aphylla mediated neuroprotective potential in rotenone-induced Parkinson’s disease: In silico and in vivo study
Source: PLoS One. 2026 Jan 6;21(1):e0339010. doi: 10.1371/journal.pone.0339010 (PMC12774373; doi:10.1371/journal.pone.0339010)
Supplement: S4 Table — (DOCX) [file pone.0339010.s004.docx]

**Table S4. Results of Swiss Target Prediction for Compound 1.**

| **No.** | **Name** |
| --- | --- |
| 1 | 11-Beta-hydroxysteroid dehydrogenase 1 |
| 2 | Absent in melanoma 2 |
| 3 | ADAM17 |
| 4 | Aldose reductase |
| 5 | Alkaline phosphatase placental-like |
| 6 | Alkaline phosphatase, tissue-nonspecific isozyme |
| 7 | Angiotensin-converting enzyme (by homology) |
| 8 | Apoptosis regulator Bcl-2 |
| 9 | Arachidonate 5-lipoxygenase |
| 10 | Aryl hydrocarbon receptor |
| 11 | Arylamine N-acetyltransferase 1 |
| 12 | Beta amyloid A4 protein |
| 13 | Beta-secretase 1 |
| 14 | Calcium-activated potassium channel subunit alpha-1 |
| 15 | Carbonic anhydrase I |
| 16 | Carbonic anhydrase II |
| 17 | Carbonic anhydrase IX |
| 18 | Carbonic anhydrase VA |
| 19 | Carbonic anhydrase VB |
| 20 | Carbonic anhydrase VI |
| 21 | Carbonic anhydrase VII |
| 22 | Carbonic anhydrase XII |
| 23 | Carbonic anhydrase XIII |
| 24 | Carbonic anhydrase XIV |
| 25 | Casein kinase I alpha |
| 26 | Casein kinase I delta |
| 27 | CDGSH iron-sulfur domain-containing protein 1 |
| 28 | c-Jun N-terminal kinase 1 |
| 29 | Coagulation factor VII/tissue factor |
| 30 | Cyclin-dependent kinase 1/cyclin B |
| 31 | Cyclin-dependent kinase 1/cyclin B1 |
| 32 | Cyclin-dependent kinase 2/cyclin E |
| 33 | Cyclin-dependent kinase 4/cyclin D1 |
| 34 | Cyclin-dependent kinase 5/CDK5 activator 1 |
| 35 | Cyclooxygenase-1 |
| 36 | Cyclooxygenase-2 |
| 37 | Cytochrome P450 19A1 |
| 38 | Cytochrome P450 1A1 |
| 39 | Cytochrome P450 1A2 |
| 40 | Cytochrome P450 1B1 |
| 41 | Cytochrome P450 3A4 |
| 42 | D-Amino-acid oxidase |
| 43 | DNA topoisomerase II alpha |
| 44 | Epidermal growth factor receptor erbB1 |
| 45 | Estrogen receptor alpha |
| 46 | Estrogen receptor beta |
| 47 | Fructose-1,6-bisphosphatase |
| 48 | Glyoxalase I |
| 49 | G-protein coupled receptor kinase 2 |
| 50 | Heme oxygenase 1 |
| 51 | Histone acetyltransferase p300 |
| 52 | Histone deacetylase 4 |
| 53 | Histone deacetylase 5 |
| 54 | Histone deacetylase 7 |
| 55 | HMG-CoA reductase |
| 56 | Inhibitor of NF-kappa-B kinase (IKK) |
| 57 | Interleukin 18 |
| 58 | Interleukin 4 |
| 59 | Leukocyte common antigen |
| 60 | Lysine-specific demethylase 4A |
| 61 | Lysine-specific demethylase 4B |
| 62 | Lysine-specific demethylase 5B |
| 63 | Lysine-specific demethylase 5C |
| 64 | Matrix metalloproteinase 1 |
| 65 | Matrix metalloproteinase 13 |
| 66 | Matrix metalloproteinase 2 |
| 67 | Matrix metalloproteinase 3 |
| 68 | Matrix metalloproteinase 8 |
| 69 | Matrix metalloproteinase 9 |
| 70 | Metabotropic glutamate receptor 4 |
| 71 | Metabotropic glutamate receptor 5 |
| 72 | Monoamine oxidase A |
| 73 | Monoamine oxidase B |
| 74 | Monocarboxylate transporter 1 (by homology) |
| 75 | Myoglobin |
| 76 | Myosin light chain kinase, smooth muscle |
| 77 | Nuclear factor erythroid 2-related factor 2 |
| 78 | Nuclear factor NF-kappa-B p65 subunit |
| 79 | Palmitoleoyl-protein carboxylesterase NOTUM |
| 80 | P-Glycoprotein 1 |
| 81 | Phosphodiesterase 5A |
| 82 | Phospholipase A-2-activating protein |
| 83 | Plectin |
| 84 | Poly [ADP-ribose] polymerase-1 |
| 85 | Protein kinase C mu |
| 86 | Protein-tyrosine phosphatase 1B |
| 87 | Quinone reductase 2 |
| 88 | Serine/threonine-protein kinase D2 |
| 89 | Serine/threonine-protein kinase Nek1 |
| 90 | Serine/threonine-protein kinase PIM1 |
| 91 | Serine/threonine-protein kinase PIM2 |
| 92 | Serine/threonine-protein kinase PIM3 |
| 93 | Signal transducer and activator of transcription 3 |
| 94 | Transthyretin |
| 95 | Tubulin beta-1 chain |
| 96 | Tubulin beta-3 chain |
| 97 | Tumor Necrosis Factor |
| 98 | Xanthine dehydrogenase |
