# Supplementary material for: Emphasizing the role of oxidative stress and Sirt-1/Nrf2 and TLR-4/NF-κB in Tamarix aphylla mediated neuroprotective potential in rotenone-induced Parkinson’s disease: In silico and in vivo study
Source: PLoS One. 2026 Jan 6;21(1):e0339010. doi: 10.1371/journal.pone.0339010 (PMC12774373; doi:10.1371/journal.pone.0339010)
Supplement: S5 Table — (DOCX) [file pone.0339010.s005.docx]

**Table S5. Results of Swiss Target Prediction for Compound 2.**

| **No.** | **Name** |
| --- | --- |
| 1 | Acetylcholinesterase |
| 2 | Adenosine A2b receptor |
| 3 | Adrenergic receptor alpha-2 |
| 4 | Aldehyde dehydrogenase |
| 5 | Alkaline phosphatase, tissue-nonspecific isozyme |
| 6 | Alpha-2a adrenergic receptor |
| 7 | Alpha-2b adrenergic receptor |
| 8 | Amine oxidase, copper containing |
| 9 | Androgen receptor |
| 10 | Arylamine N-acetyltransferase 1 |
| 11 | Beta-secretase 1 |
| 12 | Calcium-activated potassium channel subunit alpha-1 |
| 13 | Carbonic anhydrase I |
| 14 | Carbonic anhydrase II |
| 15 | Carbonic anhydrase III |
| 16 | Carbonic anhydrase IX |
| 17 | Carbonic anhydrase VI |
| 18 | Carbonic anhydrase VII |
| 19 | Carbonic anhydrase XII |
| 20 | Carbonic anhydrase XIII |
| 21 | Carbonic anhydrase XIV |
| 22 | Carbonyl reductase [NADPH] 1 |
| 23 | Casein kinase I alpha |
| 24 | Casein kinase I delta |
| 25 | Caspase-6 |
| 26 | Cathepsin (V and K) |
| 27 | CDC7/DBF4 (cell division cycle 7-related protein kinase/activator of S phase kinase) |
| 28 | CDGSH iron-sulfur domain-containing protein 1 |
| 29 | CDK9/cyclin T1 |
| 30 | c-Jun N-terminal kinase 1 |
| 31 | Coagulation factor XII |
| 32 | Cyclin-dependent kinase 1/cyclin B |
| 33 | Cyclin-dependent kinase 2/cyclin A |
| 34 | Cytochrome P450 1A2 |
| 35 | Dual specificity tyrosine-phosphorylation-regulated kinase 1B |
| 36 | Dual-specificity tyrosine-phosphorylation regulated kinase 1A (by homology) |
| 37 | Egl nine homolog 1 |
| 38 | Epidermal growth factor receptor erbB1 |
| 39 | Estradiol 17-beta-dehydrogenase 3 |
| 40 | Estrogen receptor beta |
| 41 | Fibroblast growth factor receptor 1 |
| 42 | Focal adhesion kinase 1 |
| 43 | G-Protein-coupled receptor kinase 6 |
| 44 | GABA A receptor alpha-3/beta-2/gamma-2 |
| 45 | GABA-A receptor; alpha-1/beta-2/gamma-2 |
| 46 | Gamma-amino-N-butyrate transaminase (by homology) |
| 47 | Glutathione reductase |
| 48 | Glutathione S-transferase A1 |
| 49 | Glycogen synthase kinase-3 alpha |
| 50 | Heat shock 70 kDa protein 1 |
| 51 | Hepatocyte growth factor receptor |
| 52 | Histone chaperone ASF1A |
| 53 | Histone deacetylase 2 |
| 54 | Histone deacetylase 4 |
| 55 | Histone deacetylase 5 |
| 56 | Histone deacetylase 7 |
| 57 | Histone deacetylase 8 |
| 58 | HMG-CoA reductase |
| 59 | Interferon alpha 2 |
| 60 | Interferon alpha/beta receptor 1 |
| 61 | Interferon beta 1 |
| 62 | Interleukin 17A |
| 63 | Interleukin-8 receptor A |
| 64 | Kinesin-1 heavy chain/tyrosine-protein kinase receptor RET |
| 65 | LDL-associated phospholipase A2 |
| 66 | Leukocyte elastase |
| 67 | L-Lactate dehydrogenase A chain |
| 68 | Lysine-specific histone demethylase 1 |
| 69 | Macrophage scavenger receptor types I and II |
| 70 | Mitogen-activated protein kinase kinase kinase 8 |
| 71 | Monoamine oxidase A |
| 72 | Monoamine oxidase B |
| 73 | Muscarinic acetylcholine receptor M1 (by homology) |
| 74 | Myoglobin |
| 75 | Nischarin |
| 76 | NLR family pyrin domain containing 1 |
| 77 | N-Lysine methyltransferase SETD8 |
| 78 | NUAK family SNF1-like kinase 1 |
| 79 | Phosphodiesterase 5A |
| 80 | PI3-Kinase p110-gamma subunit |
| 81 | Plectin |
| 82 | Poly [ADP-ribose] polymerase-1 |
| 83 | Progesterone receptor |
| 84 | Serine/threonine-protein kinase Chk1 |
| 85 | Serine/threonine-protein kinase PIM2 |
| 86 | Serine/threonine-protein kinase/endoribonuclease IRE1 |
| 87 | Serine-protein kinase ATR |
| 88 | Serotonin 2b (5-HT2b) receptor |
| 89 | Thrombin |
| 90 | Thymidylate synthase |
| 91 | Trace amine-associated receptor 1(by homology) |
| 92 | Tyrosine-protein kinase JAK1 |
| 93 | Tyrosine-protein kinase JAK2 |
| 94 | Tyrosine-protein kinase JAK3 |
| 95 | Tyrosine-protein kinase TYK2 |
| 96 | Vascular endothelial growth factor receptor 1 |
| 97 | Vascular endothelial growth factor receptor 2 |
| 98 | Voltage-gated potassium channel subunit Kv1.3 |
