# Supplementary material for: Emphasizing the role of oxidative stress and Sirt-1/Nrf2 and TLR-4/NF-κB in Tamarix aphylla mediated neuroprotective potential in rotenone-induced Parkinson’s disease: In silico and in vivo study
Source: PLoS One. 2026 Jan 6;21(1):e0339010. doi: 10.1371/journal.pone.0339010 (PMC12774373; doi:10.1371/journal.pone.0339010)
Supplement: S6 Table — (DOCX) [file pone.0339010.s006.docx]

**Table S6. Results of Swiss Target Prediction for Compound 3.**

| **No.** | **Name** |
| --- | --- |
| 1 | 14-3-3 Protein gamma |
| 2 | Acetylcholinesterase |
| 3 | Aldose reductase |
| 4 | ALK tyrosine kinase receptor |
| 5 | Alkaline phosphatase, tissue-nonspecific isozyme |
| 6 | Alpha-synuclein |
| 7 | Arachidonate 5-lipoxygenase |
| 8 | Beta amyloid A4 protein |
| 9 | Beta-secretase 1 |
| 10 | Carbonic anhydrase I |
| 11 | Carbonic anhydrase II |
| 12 | Carbonic anhydrase IV |
| 13 | Carbonic anhydrase IX |
| 14 | Carbonic anhydrase VA |
| 15 | Carbonic anhydrase VI |
| 16 | Carbonic anhydrase VII |
| 17 | Carbonic anhydrase XII |
| 18 | Carbonic anhydrase XIII |
| 19 | Carbonic anhydrase XIV |
| 20 | Carbonyl reductase [NADPH] 1 |
| 21 | Casein kinase II alpha |
| 22 | Cyclin-dependent kinase 2/cyclin A |
| 23 | Cyclin-dependent kinase 2/cyclin E1 |
| 24 | Cyclin-dependent kinase 4/cyclin D1 |
| 25 | Cyclin-dependent kinase 5/CDK5 activator 1 |
| 26 | Cyclooxygenase-1 |
| 27 | Cyclooxygenase-2 |
| 28 | Cytochrome P450 1A2 |
| 29 | D-Amino-acid oxidase |
| 30 | Dihydrofolate reductase |
| 31 | DNA topoisomerase I |
| 32 | Ephrin receptor |
| 33 | Epidermal growth factor receptor erbB1 |
| 34 | Estradiol 17-beta-dehydrogenase 1 |
| 35 | Estradiol 17-beta-dehydrogenase 3 |
| 36 | Estrogen receptor alpha |
| 37 | Estrogen receptor beta |
| 38 | Fibroblast growth factor receptor 1 |
| 39 | Focal adhesion kinase 1 |
| 40 | Glutamate receptor ionotropic, AMPA 1 |
| 41 | Glutamate receptor ionotropic, AMPA 2 |
| 42 | Glutamate receptor ionotropic, AMPA 3 |
| 43 | Glutamate receptor ionotropic, AMPA 4 |
| 44 | Glutathione reductase |
| 45 | Glycogen synthase kinase-3 beta |
| 46 | G-protein coupled receptor 35 |
| 47 | Heat shock 70 kDa protein 1 |
| 48 | Hepatocyte growth factor receptor |
| 49 | Histone deacetylase 6 |
| 50 | Insulin receptor |
| 51 | Insulin-like growth factor binding protein 3 |
| 52 | Insulin-like growth factor I receptor |
| 53 | Interferon alpha 2 |
| 54 | Interferon alpha/beta receptor 1 |
| 55 | Interferon beta 1 |
| 56 | Interleukin 17A |
| 57 | Mannose-6-phosphate isomerase |
| 58 | Matrix metalloproteinase 2 |
| 59 | Matrix metalloproteinase 9 |
| 60 | Microtubule-associated protein tau |
| 61 | Mitogen-activated protein kinase kinase kinase 8 |
| 62 | Monoamine oxidase A |
| 63 | NLR family pyrin domain containing 1 |
| 64 | NUAK family SNF1-like kinase 1 |
| 65 | Nuclear factor NF-kappa-B p105 subunit |
| 66 | Phospholipase A-2-activating protein |
| 67 | PI3-Kinase p110-gamma subunit |
| 68 | Plasminogen activator inhibitor-1 |
| 69 | Platelet-derived growth factor receptor beta |
| 70 | Potassium-transporting ATPase |
| 71 | Protein-tyrosine phosphatase 2C |
| 72 | Receptor protein-tyrosine kinase erbB-2 |
| 73 | Serine/threonine-protein kinase AKT |
| 74 | Serine/threonine-protein kinase aurora-A |
| 75 | Serine/threonine-protein kinase aurora-B |
| 76 | Serine/threonine-protein kinase B-raf |
| 77 | Serine/threonine-protein kinase Chk1 |
| 78 | Serine/threonine-protein kinase PIM1 |
| 79 | Serine/threonine-protein kinase PLK1 |
| 80 | Serine/threonine-protein kinase PLK4 |
| 81 | Squalene monooxygenase (by homology) |
| 82 | Stem cell growth factor receptor |
| 83 | Thrombin |
| 84 | Thymidine phosphorylase |
| 85 | Thymidylate synthase |
| 86 | Transmembrane domain-containing protein TMIGD3 |
| 87 | Troponin, cardiac muscle |
| 88 | Tyrosine-protein kinase FGR (by homology) |
| 89 | Tyrosine-protein kinase Lyn (by homology) |
| 90 | Tyrosine-protein kinase receptor UFO |
| 91 | Tyrosine-protein kinase SRC |
| 92 | Tyrosine-protein kinase TIE-2 |
| 93 | Tyrosyl-DNA phosphodiesterase 1 |
| 94 | Uridine phosphorylase 1 (by homology) |
| 95 | Vascular endothelial growth factor receptor 2 |
| 96 | Vascular endothelial growth factor receptor 3 |
| 97 | Voltage-gated potassium channel subunit Kv1.3 |
| 98 | Voltage-gated potassium channel subunit Kv1.5 |
| 99 | Xanthine dehydrogenase |
