# Supplementary material for: Emphasizing the role of oxidative stress and Sirt-1/Nrf2 and TLR-4/NF-κB in Tamarix aphylla mediated neuroprotective potential in rotenone-induced Parkinson’s disease: In silico and in vivo study
Source: PLoS One. 2026 Jan 6;21(1):e0339010. doi: 10.1371/journal.pone.0339010 (PMC12774373; doi:10.1371/journal.pone.0339010)
Supplement: S7 Table — (DOCX) [file pone.0339010.s007.docx]

**Table S7. Results of Swiss Target Prediction for Compound 4.**

| **No.** | **Name** |
| --- | --- |
| 1 | 3-Phosphoinositide dependent protein kinase-1 |
| 2 | 6-Phosphofructo-2-kinase/fructose-2,6-bisphosphatase 3 |
| 3 | 6-Phosphofructo-2-kinase/fructose-2,6-bisphosphatase 4 |
| 4 | Acetylcholinesterase |
| 5 | ADAMTS5 |
| 6 | Adenosine A3 receptor |
| 7 | Adenosine kinase |
| 8 | Bifunctional protein NCOAT |
| 9 | Carbonic anhydrase I |
| 10 | Carbonic anhydrase II |
| 11 | Carbonic anhydrase IX |
| 12 | Carbonic anhydrase VA |
| 13 | Carbonic anhydrase VI |
| 14 | Carbonic anhydrase VII |
| 15 | Carbonic anhydrase XII |
| 16 | Carbonic anhydrase XIV |
| 17 | Casein kinase II alpha |
| 18 | Caspase-1 |
| 19 | Caspase-2 |
| 20 | Caspase-3 |
| 21 | Caspase-6 |
| 22 | Caspase-7 |
| 23 | Caspase-8 |
| 24 | C-C Chemokine receptor type 2 |
| 25 | C-C Motif chemokine ligand 5 |
| 26 | CDC7/DBF4 (cell division cycle 7-related protein kinase/activator of S phase kinase) |
| 27 | CDGSH iron-sulfur domain-containing protein 1 |
| 28 | CDK9/cyclin T1 |
| 29 | C-X-C Motif chemokine ligand 1 |
| 30 | Cyclin-dependent kinase 1 |
| 31 | Cyclin-dependent kinase 9 |
| 32 | Cytidine deaminase |
| 33 | D-amino-acid oxidase |
| 34 | Dihydroorotate dehydrogenase |
| 35 | DNA excision repair protein ERCC-5 |
| 36 | DNA (apurinic or apyrimidinic site) lyase |
| 37 | Dual specificity mitogen-activated protein kinase kinase 1 |
| 38 | Dual specificity protein kinase CLK2 (by homology) |
| 39 | Dual specificity protein kinase CLK4 (by homology) |
| 40 | Dual specificity protein kinase CLK1 (by homology) |
| 41 | Dual-specificity tyrosine-phosphorylation regulated kinase 3 |
| 42 | Endothelin-converting enzyme 1 |
| 43 | Farnesyl diphosphate synthase |
| 44 | Fatty acid binding protein adipocyte |
| 45 | Fatty acid binding protein intestinal |
| 46 | Fatty acid binding protein muscle |
| 47 | Flap endonuclease 1 |
| 48 | Glutamate carboxypeptidase II |
| 49 | Glutamate receptor ionotropic kainate 1 |
| 50 | Glutathione S-transferase Mu 2 |
| 51 | Glutathione S-transferase Pi |
| 52 | G-protein coupled receptor 35 |
| 53 | G-protein coupled receptor 55 |
| 54 | Hydroxyacid oxidase 1 |
| 55 | Hydroxyacid oxidase 2 (by homology) |
| 56 | Hypoxia-inducible factor prolyl 4-hydroxylase |
| 57 | Intercellular adhesion molecule (ICAM-1), integrin alpha-L/beta-2 |
| 58 | Interferon alpha 1 |
| 59 | Kynurenine 3-monooxygenase |
| 60 | Liver glycogen phosphorylase |
| 61 | Lysine-specific demethylase 4A |
| 62 | Lysine-specific demethylase 4B |
| 63 | Lysine-specific demethylase 4C |
| 64 | Lysine-specific demethylase 4D |
| 65 | Lysine-specific demethylase 4D-like |
| 66 | Lysine-specific demethylase 5A |
| 67 | Lysine-specific demethylase 5B |
| 68 | Lysine-specific demethylase 5C |
| 69 | Lysine-specific demethylase 6B |
| 70 | Macrophage migration inhibitory factor |
| 71 | Matrix metalloproteinase 12 |
| 72 | Methionine aminopeptidase 2 |
| 73 | Monocarboxylate transporter 4 |
| 74 | Muscarinic acetylcholine receptor M1 |
| 75 | Muscle glycogen phosphorylase |
| 76 | NADPH oxidase 4 |
| 77 | Neuronal acetylcholine receptor; alpha4/beta2 |
| 78 | Neurotensin receptor 3 |
| 79 | Peptidyl-prolyl cis-trans isomerase NIMA-interacting 1 |
| 80 | Peroxisome proliferator-activated receptor gamma |
| 81 | Phosphodiesterase 10A |
| 82 | Phosphodiesterase 4B |
| 83 | Poly [ADP-ribose] polymerase 10 |
| 84 | Poly [ADP-ribose] polymerase 15 |
| 85 | Poly [ADP-ribose] polymerase-1 |
| 86 | Prostanoid EP1 receptor |
| 87 | Protein tyrosine kinase 2 beta |
| 88 | Renin |
| 89 | Ribonuclease H1 |
| 90 | Serine/threonine-protein kinase PIM1 |
| 91 | Solute carrier family 22 member 12 |
| 92 | Solute carrier organic anion transporter family member 1B1 |
| 93 | Squalene synthetase (by homology) |
| 94 | T1R1/T1R3_UNCURATED |
| 95 | Thrombin |
| 96 | Thromboxane-A synthase |
| 97 | Thymidine phosphorylase (by homology) |
| 98 | Toll-like receptor 4 |
| 99 | Voltage-gated potassium channel subunit Kv1.3 |
