# Supplementary material for: Emphasizing the role of oxidative stress and Sirt-1/Nrf2 and TLR-4/NF-κB in Tamarix aphylla mediated neuroprotective potential in rotenone-induced Parkinson’s disease: In silico and in vivo study
Source: PLoS One. 2026 Jan 6;21(1):e0339010. doi: 10.1371/journal.pone.0339010 (PMC12774373; doi:10.1371/journal.pone.0339010)
Supplement: S9 Table — (DOCX) [file pone.0339010.s009.docx]

**Table S9. Results of Swiss Target Prediction for Compound 6.**

| **No.** | **Name** |
| --- | --- |
| 1 | Acetylcholinesterase |
| 2 | Adenosine A1 receptor (by homology) |
| 3 | Adenosine A2a receptor (by homology) |
| 4 | Aldehyde reductase (by homology) |
| 5 | Aldo-keto reductase family 1 member C1 (by homology) |
| 6 | Aldo-keto reductase family 1 member C2 (by homology) |
| 7 | Aldo-keto reductase family 1 member C4 (by homology) |
| 8 | Aldo-keto-reductase family 1 member C3 (by homology) |
| 9 | Aldose reductase |
| 10 | ALK tyrosine kinase receptor |
| 11 | Arachidonate 12-lipoxygenase |
| 12 | Arachidonate 15-lipoxygenase |
| 13 | Arachidonate 5-lipoxygenase |
| 14 | Arginase-1 (by homology) |
| 15 | Aryl hydrocarbon receptor |
| 16 | ATP-binding cassette sub-family G member 2 |
| 17 | Beta amyloid A4 protein |
| 18 | Beta-secretase 1 |
| 19 | CaM kinase II beta |
| 20 | Carbonic anhydrase I |
| 21 | Carbonic anhydrase II |
| 22 | Carbonic anhydrase III |
| 23 | Carbonic anhydrase IV |
| 24 | Carbonic anhydrase IX |
| 25 | Carbonic anhydrase VA |
| 26 | Carbonic anhydrase VI |
| 27 | Carbonic anhydrase VII |
| 28 | Carbonic anhydrase XII |
| 29 | Carbonic anhydrase XIII (by homology) |
| 30 | Carbonic anhydrase XIV |
| 31 | Casein kinase II alpha |
| 32 | Caspase 1 |
| 33 | Caspase 9 |
| 34 | Caspase recruitment domain family member 8 |
| 35 | Cyclin-dependent kinase 1 |
| 36 | Cyclin-dependent kinase 1/cyclin B |
| 37 | Cyclin-dependent kinase 2 |
| 38 | Cyclin-dependent kinase 5/CDK5 activator 1 |
| 39 | Cyclin-dependent kinase 6 |
| 40 | Cytochrome P450 19A1 |
| 41 | Cytochrome P450 1B1 |
| 42 | Death-associated protein kinase 1 |
| 43 | DNA topoisomerase II alpha |
| 44 | DNA-(apurinic or apyrimidinic site) lyase |
| 45 | DNA-3-methyladenine glycosylase |
| 46 | Dopamine D4 receptor |
| 47 | Epidermal growth factor receptor erbB1 |
| 48 | Estradiol 17-beta-dehydrogenase 1 |
| 49 | Estradiol 17-beta-dehydrogenase 2 |
| 50 | Estrogen receptor beta |
| 51 | Estrogen-related receptor alpha |
| 52 | Focal adhesion kinase 1 |
| 53 | Glycogen synthase kinase-3 beta |
| 54 | Glyoxalase I |
| 55 | G-protein coupled receptor 35 |
| 56 | Hepatocyte growth factor receptor |
| 57 | Induced myeloid leukemia cell differentiation protein Mcl-1 |
| 58 | Insulin receptor |
| 59 | Insulin-like growth factor I receptor |
| 60 | Interleukin 1 alpha |
| 61 | Interleukin-8 receptor A |
| 62 | Liver glycogen phosphorylase |
| 63 | Lysine-specific demethylase 4D-like |
| 64 | Matrix metalloproteinase 13 |
| 65 | Matrix metalloproteinase 2 |
| 66 | Matrix metalloproteinase 3 |
| 67 | Matrix metalloproteinase 9 |
| 68 | Microtubule-associated protein tau |
| 69 | Monoamine oxidase A |
| 70 | Multidrug resistance-associated protein 1 |
| 71 | Myeloperoxidase |
| 72 | Myosin light chain kinase, smooth muscle |
| 73 | NADPH oxidase 4 |
| 74 | Nitric oxide synthase 2 |
| 75 | NUAK family SNF1-like kinase 1 |
| 76 | P-Glycoprotein 1 |
| 77 | Phospholipase A2 group 1B |
| 78 | PI3-Kinase p110-gamma subunit |
| 79 | PI3-Kinase p85-alpha subunit |
| 80 | Plasminogen |
| 81 | Protein kinase N1 |
| 82 | Receptor-type tyrosine-protein phosphatase S |
| 83 | Serine/threonine-protein kinase AKT |
| 84 | Serine/threonine-protein kinase aurora-B |
| 85 | Serine/threonine-protein kinase NEK2 |
| 86 | Serine/threonine-protein kinase NEK6 |
| 87 | Serine/threonine-protein kinase PIM1 |
| 88 | Serine/threonine-protein kinase PLK1 |
| 89 | Solute carrier family 22 member 12 |
| 90 | Telomerase reverse transcriptase |
| 91 | Thrombin |
| 92 | Tyrosinase |
| 93 | Tyrosine-protein kinase receptor FLT3 |
| 94 | Tyrosine-protein kinase receptor UFO |
| 95 | Tyrosine-protein kinase SRC |
| 96 | Tyrosine-protein kinase SYK |
| 97 | Vascular endothelial growth factor receptor 2 |
| 98 | Vasopressin V2 receptor |
| 99 | Xanthine dehydrogenase |
