# Supplementary material for: Emphasizing the role of oxidative stress and Sirt-1/Nrf2 and TLR-4/NF-κB in Tamarix aphylla mediated neuroprotective potential in rotenone-induced Parkinson’s disease: In silico and in vivo study
Source: PLoS One. 2026 Jan 6;21(1):e0339010. doi: 10.1371/journal.pone.0339010 (PMC12774373; doi:10.1371/journal.pone.0339010)
Supplement: S11 Table — (DOCX) [file pone.0339010.s011.docx]

**Table S11. Results of Swiss Target Prediction for Compound 8.**

| **NO.** | **Name** |
| --- | --- |
| 1 | Acetylcholinesterase |
| 2 | ADAMTS5 |
| 3 | Aldo-keto reductase family 1 member C1 |
| 4 | Aldo-keto reductase family 1 member C3 |
| 5 | Aldose reductase |
| 6 | Arachidonate 5-lipoxygenase |
| 7 | Beta amyloid A4 protein |
| 8 | Beta-glucuronidase |
| 9 | Beta-secretase 1 |
| 10 | Carbonic anhydrase I |
| 11 | Carbonic anhydrase II |
| 12 | Carbonic anhydrase III |
| 13 | Carbonic anhydrase IV |
| 14 | Carbonic anhydrase IX |
| 15 | Carbonic anhydrase VA |
| 16 | Carbonic anhydrase VB |
| 17 | Carbonic anhydrase VI |
| 18 | Carbonic anhydrase VII |
| 19 | Carbonic anhydrase XII |
| 20 | Carbonic anhydrase XIII |
| 21 | Carbonic anhydrase XIV |
| 22 | Carbonyl reductase [NADPH] 1 |
| 23 | Casein kinase II alpha |
| 24 | Caspase 8 |
| 25 | C-C Motif chemokine ligand 4 |
| 26 | CDK9/cyclin T1 |
| 27 | c-Jun N-terminal kinase 1 |
| 28 | c-Jun N-terminal kinase 2 |
| 29 | Cyclin-dependent kinase 1/cyclin B1 |
| 30 | Cyclin-dependent kinase 2/cyclin A |
| 31 | Cyclin-dependent kinase 2/cyclin E1 |
| 32 | Cyclin-dependent kinase 4/cyclin D1 |
| 33 | Cyclooxygenase-2 |
| 34 | Cytochrome P450 1A2 |
| 35 | Dihydroorotate dehydrogenase |
| 36 | DNA-(apurinic or apyrimidinic site) lyase |
| 37 | Dynamin-1 |
| 38 | Ephrin receptor |
| 39 | Epidermal growth factor receptor erbB1 |
| 40 | Estradiol 17-beta-dehydrogenase 3 |
| 41 | Estrogen receptor alpha |
| 42 | Estrogen receptor beta |
| 43 | Focal adhesion kinase 1 |
| 44 | G-Protein-coupled receptor kinase 6 |
| 45 | Glutathione reductase |
| 46 | Glycogen synthase kinase-3 beta |
| 47 | G-Protein coupled receptor 35 |
| 48 | Heat shock 70 kDa protein 1 |
| 49 | Hepatocyte growth factor receptor |
| 50 | Hypoxia-inducible factor 1 alpha |
| 51 | Insulin receptor |
| 52 | Insulin-like growth factor I receptor |
| 53 | Interleukin 37 |
| 54 | Kinesin-1 heavy chain/ tyrosine-protein kinase receptor RET |
| 55 | LDL-associated phospholipase A2 |
| 56 | Mediterranean Fever Gene |
| 57 | Mitogen-activated protein kinase kinase kinase 8 |
| 58 | Monoamine oxidase A |
| 59 | Nerve growth factor receptor Trk-A |
| 60 | Neuronal acetylcholine receptor protein alpha-7 subunit |
| 61 | NUAK family SNF1-like kinase 1 |
| 62 | Peroxidasin |
| 63 | Peroxisome proliferator-activated receptor gamma |
| 64 | Phosphodiesterase 5A |
| 65 | PI3-Kinase p110-alpha subunit |
| 66 | PI3-Kinase p110-beta subunit |
| 67 | PI3-Kinase p110-delta subunit |
| 68 | PI3-Kinase p110-gamma subunit |
| 69 | Plasminogen activator inhibitor-1 |
| 70 | Platelet-derived growth factor receptor alpha |
| 71 | Platelet-derived growth factor receptor beta |
| 72 | Poly [ADP-ribose] polymerase-1 |
| 73 | Protein tyrosine kinase 2 beta |
| 74 | Receptor protein-tyrosine kinase erbB-2 |
| 75 | Ribosomal protein S6 kinase alpha 3 |
| 76 | Serine/threonine-protein kinase AKT |
| 77 | Serine/threonine-protein kinase Aurora-A |
| 78 | Serine/threonine-protein kinase Aurora-B |
| 79 | Serine/threonine-protein kinase B-raf |
| 80 | Serine/threonine-protein kinase Chk1 |
| 81 | Serine/threonine-protein kinase mTOR |
| 82 | Serine/threonine-protein kinase PLK1 |
| 83 | Serine/threonine-protein kinase PLK4 |
| 84 | Serine/threonine-protein kinase RAF |
| 85 | Squalene monooxygenase (by homology) |
| 86 | Steroid 5-alpha-reductase 1 |
| 87 | Subtilisin/kexin type 7 |
| 88 | Troponin, cardiac muscle |
| 89 | Tyrosine-protein kinase ABL |
| 90 | Tyrosine-protein kinase FGR (by homology) |
| 91 | Tyrosine-protein kinase HCK |
| 92 | Tyrosine-protein kinase Lyn (by homology) |
| 93 | Tyrosine-protein kinase SRC |
| 94 | Tyrosine-protein kinase TIE-2 |
| 95 | Vascular endothelial growth factor receptor 1 |
| 96 | Vascular endothelial growth factor receptor 2 |
| 97 | Vascular endothelial growth factor receptor 3 |
| 98 | Voltage-gated potassium channel subunit Kv1.5 |
| 99 | Xanthine dehydrogenase |
