# Supplementary material for: Emphasizing the role of oxidative stress and Sirt-1/Nrf2 and TLR-4/NF-κB in Tamarix aphylla mediated neuroprotective potential in rotenone-induced Parkinson’s disease: In silico and in vivo study
Source: PLoS One. 2026 Jan 6;21(1):e0339010. doi: 10.1371/journal.pone.0339010 (PMC12774373; doi:10.1371/journal.pone.0339010)
Supplement: S12 Table — (DOCX) [file pone.0339010.s012.docx]

**Table S12. Results of Swiss Target Prediction for Compound 9.**

| **No.** | **Name** |
| --- | --- |
| 1 | 2'-Deoxynucleoside 5'-phosphate N-hydrolase 1 |
| 2 | Actin Beta |
| 3 | Adenosine A1 receptor |
| 4 | Adenosine A3 receptor |
| 5 | AICAR transformylase |
| 6 | Aldose reductase (by homology) |
| 7 | Aminopeptidase A |
| 8 | Angiotensin-converting enzyme |
| 9 | Asparagine synthetase |
| 10 | ATP-citrate synthase |
| 11 | Bifunctional protein NCOAT |
| 12 | Breast cancer type 1 susceptibility protein |
| 13 | Caspase 4 |
| 14 | Caspase-1 |
| 15 | Caspase-2 |
| 16 | Caspase-3 |
| 17 | Caspase-6 |
| 18 | Caspase-7 |
| 19 | Caspase-8 |
| 20 | Cathepsin D |
| 21 | Cathepsin E |
| 22 | Cathepsin S |
| 23 | Cyclooxygenase-1 |
| 24 | Dihydrofolate reductase |
| 25 | Disks large homolog 4 |
| 26 | DNA (cytosine-5)-methyltransferase 1 |
| 27 | DNA (cytosine-5)-methyltransferase 3B |
| 28 | DNA topoisomerase I |
| 29 | Endo-beta-N-acetylglucosaminidase |
| 30 | Endothelin-converting enzyme 1 |
| 31 | Eukaryotic translation initation factor |
| 32 | EZH2/SUZ12/EED/RBBP7/RBBP4 |
| 33 | Folylpoly-gamma-glutamate synthetase |
| 34 | G-Protein-coupled receptor kinase 6 |
| 35 | Galectin-3 |
| 36 | GAR transformylase |
| 37 | Glucosamine-fructose-6-phosphate aminotransferase [isomerizing] 1 |
| 38 | Glutamate carboxypeptidase II |
| 39 | Glutathione S-transferase Pi |
| 40 | Glyoxalase I |
| 41 | G-Protein coupled receptor 35 |
| 42 | Hematopoietic cell protein-tyrosine phosphatase 70Z-PEP |
| 43 | Hexokinase type I |
| 44 | Hexokinase type II |
| 45 | Histone-arginine methyltransferase CARM1 |
| 46 | Histone-lysine N-methyltransferase EZH1 |
| 47 | Histone-lysine N-methyltransferase MLL |
| 48 | Histone-lysine N-methyltransferase SETDB1 |
| 49 | Histone-lysine N-methyltransferase SUV39H1 |
| 50 | Histone-lysine N-methyltransferase, H3 lysine-79 specific |
| 51 | Histone-lysine N-methyltransferase, H3 lysine-9 specific 3 |
| 52 | Histone-lysine N-methyltransferase, H3 lysine-9 specific 5 |
| 53 | Hypoxanthine-guanine phosphoribosyltransferase |
| 54 | Indoleamine 2,3-dioxygenase |
| 55 | Indolethylamine N-methyltransferase |
| 56 | Inosine-5'-monophosphate dehydrogenase 2 |
| 57 | Interleukin 1 receptor antagonist |
| 58 | Interleukin 13 |
| 59 | Interleukin-8 receptor B |
| 60 | Leucine aminopeptidase |
| 61 | Leukocyte adhesion molecule-1 |
| 62 | Low affinity sodium-glucose cotransporter |
| 63 | Lysosomal alpha-glucosidase |
| 64 | Maltase-glucoamylase |
| 65 | Mannose receptor C-type 1 |
| 66 | MAP kinase p38 alpha |
| 67 | Metabotropic glutamate receptor 2 (by homology) |
| 68 | Muscle glycogen phosphorylase |
| 69 | NAALADase II |
| 70 | Neprilysin (by homology) |
| 71 | Nicotinamide N-methyltransferase |
| 72 | N-lysine methyltransferase SMYD2 |
| 73 | Nucleotide-binding oligomerization domain-containing protein 2 |
| 74 | PI3-Kinase p110-alpha subunit |
| 75 | PI3-Kinase p110-gamma subunit |
| 76 | Protein arginine N-methyltransferase 3 |
| 77 | Protein arginine N-methyltransferase 5 |
| 78 | Protein kinase C alpha |
| 79 | Protein tyrosine phosphatase type IVA 1 |
| 80 | Protein tyrosine phosphatase type IVA 2 |
| 81 | Protein-arginine N-methyltransferase 1 |
| 82 | Protein-tyrosine phosphatase 4A3 |
| 83 | P-selectin |
| 84 | Purinergic receptor P2Y1 |
| 85 | Serine/threonine-protein kinase aurora-A |
| 86 | Serine/threonine-protein kinase PIM1 |
| 87 | Serine/threonine-protein kinase PLK1 |
| 88 | Sialidase 2 |
| 89 | Sialidase 3 |
| 90 | Sialidase 4 |
| 91 | Steroid 5-alpha-reductase 1 |
| 92 | Thymidylate synthase |
| 93 | Transketolase |
| 94 | Tyrosine-protein kinase LCK |
| 95 | Tyrosine-protein kinase SRC |
| 96 | Tyrosine-protein kinase ZAP-70 |
| 97 | Tyrosyl-DNA phosphodiesterase 1 |
| 98 | Tyrosyl-tRNA synthetase |
| 99 | Vascular endothelial growth factor receptor 2 |
