# Supplementary material for: Emphasizing the role of oxidative stress and Sirt-1/Nrf2 and TLR-4/NF-κB in Tamarix aphylla mediated neuroprotective potential in rotenone-induced Parkinson’s disease: In silico and in vivo study
Source: PLoS One. 2026 Jan 6;21(1):e0339010. doi: 10.1371/journal.pone.0339010 (PMC12774373; doi:10.1371/journal.pone.0339010)
Supplement: S14 Table — (DOCX) [file pone.0339010.s014.docx]

**Table S14. Results of Swiss Target Prediction for Compound 11.**

| **No.** | **Name** |
| --- | --- |
| 1 | Acetylcholinesterase |
| 2 | Adenosine A1 receptor (by homology) |
| 3 | Adenosine A2a receptor (by homology) |
| 4 | Adenosine A3 receptor |
| 5 | Adrenergic receptor alpha-2 |
| 6 | Aldehyde dehydrogenase |
| 7 | Aldose reductase (by homology) |
| 8 | Alpha-2a adrenergic receptor |
| 9 | Arachidonate 5-lipoxygenase |
| 10 | ATP-binding cassette sub-family G member 2 |
| 11 | Beta amyloid A4 protein |
| 12 | Beta-adrenergic receptor kinase 2 |
| 13 | cAMP-dependent protein kinase alpha-catalytic subunit |
| 14 | Carbonic anhydrase II |
| 15 | Carbonic anhydrase IV |
| 16 | Carbonic anhydrase IX |
| 17 | Carbonic anhydrase VII |
| 18 | Carbonic anhydrase XII |
| 19 | Carbonic anhydrase XIII |
| 20 | Caspase 5 |
| 21 | Catechol O-methyltransferase |
| 22 | C-C Motif chemokine ligand 3 |
| 23 | CCR4-NOT transcription complex subunit 7 |
| 24 | C-X-C Motif chemokine ligand 10 |
| 25 | Cyclin-dependent kinase 1/cyclin B1 |
| 26 | Cyclin-dependent kinase 2/cyclin A |
| 27 | Cyclin-dependent kinase 2/cyclin E |
| 28 | Cyclin-dependent kinase 4 |
| 29 | Cyclin-dependent kinase 4/cyclin D1 |
| 30 | Cyclooxygenase-2 |
| 31 | Cytochrome b-245 beta chain |
| 32 | Cytochrome P450 1A1 |
| 33 | Cytochrome P450 1B1 |
| 34 | Dopamine D2 receptor (by homology) |
| 35 | Dual specificity protein phosphatase 3 |
| 36 | Egl nine homolog 1 |
| 37 | Epidermal growth factor receptor erbB1 |
| 38 | Equilibrative nucleoside transporter 1 |
| 39 | Fructose-1,6-bisphosphatase |
| 40 | G-Protein-coupled receptor kinase 4 |
| 41 | G-Protein-coupled receptor kinase 5 |
| 42 | G-Protein-coupled receptor kinase 7 |
| 43 | G-Protein coupled receptor kinase 2 |
| 44 | Heat shock protein HSP 90-alpha |
| 45 | Heat shock protein HSP 90-beta |
| 46 | Histone deacetylase 1 |
| 47 | Histone deacetylase 10 |
| 48 | Histone deacetylase 2 |
| 49 | Histone deacetylase 3 |
| 50 | Histone deacetylase 6 |
| 51 | Histone deacetylase 8 |
| 52 | Inhibitor of nuclear factor kappa B kinase beta subunit |
| 53 | Integrin alpha-4/beta-1 |
| 54 | Integrin alpha-5/beta-1 |
| 55 | Integrin alpha-IIb/beta-3 |
| 56 | Integrin alpha-V/beta-3 |
| 57 | Integrin alpha-V/beta-6 |
| 58 | Interleukin 1 receptor accessory protein |
| 59 | Interleukin-2 |
| 60 | Interleukin-8 receptor B |
| 61 | Kallikrein 1 |
| 62 | Kallikrein 2 |
| 63 | Lymphocyte differentiation antigen CD38 |
| 64 | Metastin receptor |
| 65 | Multidrug resistance-associated protein 1 |
| 66 | NADPH oxidase 4 |
| 67 | Neuromedin-U receptor 2 |
| 68 | P-Glycoprotein 1 |
| 69 | Phosphodiesterase 5A |
| 70 | PI3-Kinase p110-alpha subunit |
| 71 | Plasminogen |
| 72 | Plasminogen activator inhibitor-1 |
| 73 | Prostaglandin E synthase |
| 74 | Proteasome assembly chaperone 3 |
| 75 | Protein kinase C (PKC) |
| 76 | Protein kinase C alpha |
| 77 | Protein kinase C beta |
| 78 | Protein kinase C delta |
| 79 | Protein kinase C epsilon |
| 80 | Protein kinase C eta |
| 81 | Protein kinase C gamma |
| 82 | Quinone reductase 2 |
| 83 | Rhodopsin kinase |
| 84 | Ribosomal protein S6 kinase alpha 3 |
| 85 | Serine/threonine-protein kinase Chk1 |
| 86 | Serine/threonine-protein kinase Chk2 |
| 87 | Serine/threonine-protein kinase RAF |
| 88 | Sigma opioid receptor |
| 89 | Squalene monooxygenase (by homology) |
| 90 | SUMO-activating enzyme |
| 91 | Telomerase reverse transcriptase |
| 92 | Thrombin and coagulation factor X |
| 93 | TNF-alpha |
| 94 | Transitional endoplasmic reticulum ATPase |
| 95 | Troponin, cardiac muscle |
| 96 | Tumor suppressor p53/oncoprotein Mdm2 |
| 97 | Urokinase-type plasminogen activator |
| 98 | Voltage-gated potassium channel subunit Kv1.3 |
| 99 | Xanthine dehydrogenase |
