# Supplementary material for: Emphasizing the role of oxidative stress and Sirt-1/Nrf2 and TLR-4/NF-κB in Tamarix aphylla mediated neuroprotective potential in rotenone-induced Parkinson’s disease: In silico and in vivo study
Source: PLoS One. 2026 Jan 6;21(1):e0339010. doi: 10.1371/journal.pone.0339010 (PMC12774373; doi:10.1371/journal.pone.0339010)
Supplement: S15 Table — (DOCX) [file pone.0339010.s015.docx]

**Table S15. Results of Swiss Target Prediction for Compound 12.**

| **No.** | **Name** |
| --- | --- |
| 1 | Acetylcholinesterase |
| 2 | Adenosine A1 receptor (by homology) |
| 3 | Adenosine A2a receptor (by homology) |
| 4 | Adenosine A3 receptor |
| 5 | Adrenergic receptor alpha-2 |
| 6 | Adrenergic receptor beta |
| 7 | Aldehyde dehydrogenase |
| 8 | Aldose reductase |
| 9 | Alpha-2a adrenergic receptor |
| 10 | Arachidonate 5-lipoxygenase |
| 11 | ATP-binding cassette sub-family G member 2 |
| 12 | Beta amyloid A4 protein |
| 13 | Beta-1 adrenergic receptor |
| 14 | cAMP-dependent protein kinase alpha-catalytic subunit |
| 15 | Carbonic anhydrase II |
| 16 | Carbonic anhydrase IV |
| 17 | Carbonic anhydrase VII |
| 18 | Carbonic anhydrase XII |
| 19 | Carbonic anhydrase XIII |
| 20 | Catechol O-methyltransferase |
| 21 | C-C Motif chemokine ligand 2 |
| 22 | CCR4-NOT transcription complex subunit 7 |
| 23 | Cyclin-dependent kinase 1/cyclin B1 |
| 24 | Cyclooxygenase-2 |
| 25 | Cytochrome P450 19A1 |
| 26 | Cytochrome P450 1B1 |
| 27 | Death-associated protein kinase 1 |
| 28 | DNA topoisomerase II alpha |
| 29 | Dopamine D3 receptor |
| 30 | Dopamine D4 receptor |
| 31 | Dual specificity protein phosphatase 3 |
| 32 | Dynamin-2 |
| 33 | Egl nine homolog 1 |
| 34 | Equilibrative nucleoside transporter 1 |
| 35 | Estradiol 17-beta-dehydrogenase 2 |
| 36 | Focal adhesion kinase 1 |
| 37 | Gasdermin D |
| 38 | Glyceraldehyde-3-phosphate dehydrogenase |
| 39 | Glycogen synthase kinase-3 beta |
| 40 | Glyoxalase I |
| 41 | G-Protein coupled receptor 35 |
| 42 | Heat shock protein HSP 90-alpha |
| 43 | Heat shock protein HSP 90-beta |
| 44 | Induced myeloid leukemia cell differentiation protein Mcl-1 |
| 45 | Inhibitor of nuclear factor kappa B kinase beta subunit |
| 46 | Insulin receptor |
| 47 | Insulin-like growth factor I receptor |
| 48 | Integrin alpha-4/beta-1 |
| 49 | Integrin alpha-5/beta-1 |
| 50 | Integrin alpha-IIb/beta-3 |
| 51 | Integrin alpha-V/beta-3 |
| 52 | Integrin alpha-V/beta-6 |
| 53 | Interleukin 2 |
| 54 | Interleukin-2 |
| 55 | Liver glycogen phosphorylase |
| 56 | Lymphocyte differentiation antigen CD38 |
| 57 | Lysine-specific demethylase 4D-like |
| 58 | Matrix metalloproteinase 13 |
| 59 | Metastin receptor |
| 60 | Microtubule-associated protein tau |
| 61 | Monoamine oxidase A |
| 62 | Myeloperoxidase |
| 63 | Myosin light chain kinase, smooth muscle |
| 64 | NADPH oxidase 4 |
| 65 | Neuromedin-U receptor 2 |
| 66 | Phosphodiesterase 5A |
| 67 | PI3-Kinase p110-alpha subunit |
| 68 | PI3-Kinase p85-alpha subunit |
| 69 | Plasminogen |
| 70 | Plasminogen activator inhibitor-1 |
| 71 | Protein kinase C (PKC) |
| 72 | Protein kinase C alpha |
| 73 | Protein kinase C beta |
| 74 | Protein kinase C delta |
| 75 | Protein kinase C epsilon |
| 76 | Protein kinase C eta |
| 77 | Protein kinase C gamma |
| 78 | Quinone reductase 2 |
| 79 | Ribosomal protein S6 kinase alpha 3 |
| 80 | Serine/threonine-protein kinase aurora-B |
| 81 | Serine/threonine-protein kinase Chk1 |
| 82 | Serine/threonine-protein kinase Chk2 |
| 83 | Serine/threonine-protein kinase PIM1 |
| 84 | Serine/threonine-protein kinase RAF |
| 85 | Serine/threonine-protein kinase WEE1 |
| 86 | Squalene monooxygenase (by homology) |
| 87 | Telomerase reverse transcriptase |
| 88 | Thrombin and coagulation factor X |
| 89 | TNF-alpha |
| 90 | Toll-like receptor 9 |
| 91 | Transitional endoplasmic reticulum ATPase |
| 92 | Troponin, cardiac muscle |
| 93 | Tyrosine-protein kinase receptor FLT3 |
| 94 | Tyrosine-protein kinase SYK |
| 95 | Tyrosyl-DNA phosphodiesterase 1 |
| 96 | Vascular endothelial growth factor receptor 2 |
| 97 | Vasopressin V2 receptor |
| 98 | Voltage-gated potassium channel subunit Kv1.3 |
| 99 | Xanthine dehydrogenase |
