# Supplementary material for: Emphasizing the role of oxidative stress and Sirt-1/Nrf2 and TLR-4/NF-κB in Tamarix aphylla mediated neuroprotective potential in rotenone-induced Parkinson’s disease: In silico and in vivo study
Source: PLoS One. 2026 Jan 6;21(1):e0339010. doi: 10.1371/journal.pone.0339010 (PMC12774373; doi:10.1371/journal.pone.0339010)
Supplement: S16 Table — (DOCX) [file pone.0339010.s016.docx]

**Table S16. Results of Swiss Target Prediction for Compound 13.**

| **No.** | **Name** |
| --- | --- |
| 1 | Absent in melanoma 2 |
| 2 | Acetylcholinesterase |
| 3 | Adenosine A1 receptor (by homology) |
| 4 | Adrenergic receptor alpha-2 |
| 5 | Aldehyde dehydrogenase |
| 6 | Aldose reductase (by homology) |
| 7 | Alpha-2a adrenergic receptor |
| 8 | Androgen receptor |
| 9 | Apoptosis regulator Bcl-2 |
| 10 | Apoptosis regulator Bcl-X |
| 11 | Arachidonate 5-lipoxygenase |
| 12 | Bcl-2-related protein A1 |
| 13 | Beta-1 adrenergic receptor |
| 14 | Beta-adrenergic receptor kinase 2 |
| 15 | Beta-secretase 1 |
| 16 | cAMP-dependent protein kinase alpha-catalytic subunit |
| 17 | Cannabinoid receptor 2 |
| 18 | Carbonic anhydrase I |
| 19 | Carbonic anhydrase II |
| 20 | Carbonic anhydrase III |
| 21 | Carbonic anhydrase IV |
| 22 | Carbonic anhydrase IX |
| 23 | Carbonic anhydrase VB |
| 24 | Carbonic anhydrase VI |
| 25 | Carbonic anhydrase VII |
| 26 | Carbonic anhydrase XII |
| 27 | Carbonic anhydrase XIII |
| 28 | Carbonic anhydrase XIV |
| 29 | Catechol O-methyltransferase |
| 30 | Cyclooxygenase-2 |
| 31 | Cytochrome P450 1A2 |
| 32 | DNA topoisomerase I |
| 33 | Dopamine transporter (by homology) |
| 34 | Dual specificity protein phosphatase 3 |
| 35 | Dynamin-2 |
| 36 | Equilibrative nucleoside transporter 1 |
| 37 | Estrogen receptor alpha |
| 38 | Estrogen receptor beta |
| 39 | G-Protein-coupled receptor kinase 4 |
| 40 | G-Protein-coupled receptor kinase 5 |
| 41 | G-Protein-coupled receptor kinase 6 |
| 42 | G-Protein-coupled receptor kinase 7 |
| 43 | G-Protein coupled receptor kinase 2 |
| 44 | Heat shock protein HSP 90-alpha |
| 45 | Heat shock protein HSP 90-beta |
| 46 | Heme oxygenase 1 |
| 47 | Induced myeloid leukemia cell differentiation protein Mcl-1 |
| 48 | Inhibitor of nuclear factor kappa B kinase beta subunit |
| 49 | Integrin alpha-5/beta-1 |
| 50 | Integrin alpha-IIb/beta-3 |
| 51 | Integrin alpha-V/beta-3 |
| 52 | Integrin alpha-V/beta-6 |
| 53 | Interleukin 18 |
| 54 | Interleukin 4 |
| 55 | Interleukin-2 |
| 56 | Lymphocyte differentiation antigen CD38 |
| 57 | Metastin receptor |
| 58 | Monoamine oxidase A |
| 59 | Monoamine oxidase B |
| 60 | Mu opioid receptor |
| 61 | Multidrug resistance-associated protein 1 |
| 62 | NAD-dependent deacetylase sirtuin 1 |
| 63 | NADPH oxidase 4 |
| 64 | Neuromedin-U receptor 2 |
| 65 | P-Glycoprotein 1 (by homology) |
| 66 | Phosphodiesterase 4D |
| 67 | Phosphodiesterase 5A |
| 68 | Plasminogen activator inhibitor-1 |
| 69 | Protein kinase C (PKC) |
| 70 | Protein kinase C alpha |
| 71 | Protein kinase C beta |
| 72 | Protein kinase C delta |
| 73 | Protein kinase C epsilon |
| 74 | Protein kinase C eta |
| 75 | Protein kinase C gamma |
| 76 | Quinone reductase 2 |
| 77 | Rhodopsin kinase |
| 78 | Ribosomal protein S6 kinase alpha 3 |
| 79 | Serine/threonine-protein kinase Chk1 |
| 80 | Serine/threonine-protein kinase Chk2 |
| 81 | Serine/threonine-protein kinase RAF |
| 82 | Serine/threonine-protein kinase WEE1 |
| 83 | Serotonin transporter (by homology) |
| 84 | Sodium/glucose cotransporter 1 |
| 85 | Sodium/glucose cotransporter 2 |
| 86 | Squalene monooxygenase (by homology) |
| 87 | Thrombin |
| 88 | Thrombin and coagulation factor X |
| 89 | TNF-alpha |
| 90 | Transitional endoplasmic reticulum ATPase |
| 91 | Troponin, cardiac muscle |
| 92 | Tumor necrosis factor |
| 93 | Tyrosinase |
| 94 | Tyrosyl-DNA phosphodiesterase 1 |
| 95 | Voltage-gated potassium channel subunit Kv1.3 |
| 96 | Voltage-gated potassium channel subunit Kv1.5 |
| 97 | Xanthine dehydrogenase |
