# Supplementary material for: Emphasizing the role of oxidative stress and Sirt-1/Nrf2 and TLR-4/NF-κB in Tamarix aphylla mediated neuroprotective potential in rotenone-induced Parkinson’s disease: In silico and in vivo study
Source: PLoS One. 2026 Jan 6;21(1):e0339010. doi: 10.1371/journal.pone.0339010 (PMC12774373; doi:10.1371/journal.pone.0339010)
Supplement: S17 Table — (DOCX) [file pone.0339010.s017.docx]

**Table S17: GO Enrichment Entry.**

| **Category** | **Description** |
| --- | --- |
| GO Biological Process | Inflammatory response |
| GO Biological Process | Immune response |
| GO Biological Process | Immune system process |
| GO Biological Process | Regulation of cytokine production |
| GO Biological Process | Defense response |
| GO Biological Process | Response to other organisms |
| GO Biological Process | Biological process involved in interspecies interaction between organisms |
| GO Biological Process | Positive regulation of cytokine production |
| GO Biological Process | Response to cytokine |
| GO Biological Process | Defense response to other organisms |
| GO Biological Process | Cellular response to cytokine stimulus |
| GO Biological Process | Response to external stimulus |
| GO Biological Process | Response to stress |
| GO Biological Process | Positive regulation of gene expression |
| GO Biological Process | Response to bacterium |
| GO Biological Process | Cytokine-mediated signaling pathway |
| GO Biological Process | Regulation of inflammatory response |
| GO Biological Process | Response to organic substance |
| GO Biological Process | Regulation of response to external stimulus |
| GO Biological Process | Innate immune response |
| GO Biological Process | Response to lipopolysaccharide |
| GO Biological Process | Cellular response to organic substance |
| GO Biological Process | Regulation of defense response |
| GO Biological Process | Cellular response to chemical stimulus |
| GO Biological Process | Cellular response to lipopolysaccharide |
| GO Biological Process | Positive regulation of interleukin-1 beta production |
| GO Biological Process | Positive regulation of metabolic process |
| GO Biological Process | Regulation of immune system process |
| GO Biological Process | Positive regulation of macromolecule metabolic process |
| GO Biological Process | Positive regulation of multicellular organismal process |
| GO Biological Process | Positive regulation of immune system process |
| GO Biological Process | Regulation of multicellular organismal process |
| GO Biological Process | Regulation of response to stress |
| GO Biological Process | Regulation of interleukin-1 production |
| GO Biological Process | Response to chemical |
| GO Biological Process | Regulation of production of molecular mediator of immune response |
| GO Biological Process | Regulation of response to stimulus |
| GO Biological Process | Positive regulation of response to external stimulus |
| GO Biological Process | Positive regulation of inflammatory response |
| GO Biological Process | Positive regulation of response to stimulus |
| GO Biological Process | Positive regulation of defense response |
| GO Biological Process | Positive regulation of immune effector process |
| GO Biological Process | Response to lipid |
| GO Biological Process | Cellular response to stimulus |
| GO Biological Process | Regulation of immune effector process |
| GO Biological Process | Regulation of immune response |
| GO Biological Process | Response to oxygen-containing compound |
| GO Biological Process | Regulation of molecular function |
| GO Biological Process | Positive regulation of production of molecular mediator of immune response |
| GO Biological Process | Cellular response to lipid |
| GO Biological Process | Pyroptosis |
| GO Biological Process | Programmed cell death |
| GO Biological Process | Positive regulation of molecular function |
| GO Biological Process | Regulation of cytokine production involved in immune response |
| GO Biological Process | Positive regulation of biological process |
| GO Biological Process | Response to stimulus |
| GO Biological Process | Signal transduction |
| GO Biological Process | Positive regulation of protein metabolic process |
| GO Biological Process | Cellular response to oxygen-containing compound |
| GO Biological Process | Positive regulation of leukocyte activation |
| GO Biological Process | Cell communication |
| GO Biological Process | Regulation of leukocyte activation |
| GO Biological Process | Cell activation |
| GO Biological Process | Negative regulation of cytokine production |
| GO Biological Process | Regulation of interleukin-6 production |
| GO Biological Process | Response to virus |
| GO Biological Process | Regulation of chemokine production |
| GO Biological Process | Apoptotic process |
| GO Biological Process | Positive regulation of cysteine-type endopeptidase activity |
| GO Biological Process | Regulation of gene expression |
| GO Biological Process | Positive regulation of immune response |
| GO Biological Process | Positive regulation of catalytic activity |
| GO Biological Process | Negative regulation of gene expression |
| GO Biological Process | Regulation of macromolecule metabolic process |
| GO Biological Process | Regulation of protein metabolic process |
| GO Biological Process | Regulation of leukocyte proliferation |
| GO Biological Process | Regulation of multicellular organismal development |
| GO Biological Process | Regulation of cell population proliferation |
| GO Biological Process | Positive regulation of interleukin-6 production |
| GO Biological Process | Positive regulation of lymphocyte activation |
| GO Biological Process | Positive regulation of chemokine production |
| GO Biological Process | Leukocyte activation |
| GO Biological Process | Cell surface receptor signaling pathway |
| GO Biological Process | Regulation of leukocyte mediated immunity |
| GO Biological Process | Negative regulation of response to stimulus |
| GO Biological Process | Regulation of lymphocyte activation |
| GO Biological Process | Positive regulation of cellular process |
| GO Biological Process | Regulation of DNA-binding transcription factor activity |
| GO Biological Process | Myeloid leukocyte activation |
| GO Biological Process | Positive regulation of nitrogen compound metabolic process |
| GO Biological Process | Defense response to bacterium |
| GO Biological Process | Positive regulation of proteolysis |
| GO Biological Process | Regulation of cell death |
| GO Biological Process | Positive regulation of signal transduction |
| GO Biological Process | Regulation of tumor necrosis factor production |
| GO Biological Process | Positive regulation of leukocyte proliferation |
| GO Biological Process | Regulation of catalytic activity |
| GO Biological Process | Regulation of signal transduction |
| GO Biological Process | Regulation of leukocyte migration |
| GO Biological Process | Regulation of programmed cell death |
| GO Biological Process | Positive regulation of hydrolase activity |
| GO Biological Process | Regulation of mononuclear cell proliferation |
| GO Biological Process | Positive regulation of lymphocyte proliferation |
| GO Biological Process | Regulation of adaptive immune response |
| GO Biological Process | Defense response to virus |
| GO Biological Process | Regulation of leukocyte differentiation |
| GO Biological Process | Regulation of I-kappaB kinase/NF-kappaB signaling |
| GO Biological Process | Regulation of cell communication |
| GO Biological Process | Regulation of signaling |
| GO Biological Process | Regulation of apoptotic process |
| GO Biological Process | Positive regulation of DNA-binding transcription factor activity |
| GO Biological Process | Regulation of hemopoiesis |
| GO Biological Process | Response to interferon-gamma |
| GO Biological Process | Positive regulation of cysteine-type endopeptidase activity involved in apoptotic process |
| GO Biological Process | Negative regulation of multicellular organismal process |
| GO Biological Process | Positive regulation of tyrosine phosphorylation of STAT protein |
| GO Biological Process | Regulation of leukocyte cell-cell adhesion |
| GO Biological Process | Regulation of cell differentiation |
| GO Biological Process | Regulation of lymphocyte proliferation |
| GO Biological Process | Pattern recognition receptor signaling pathway |
| GO Biological Process | Positive regulation of tumor necrosis factor production |
| GO Biological Process | Positive regulation of cell population proliferation |
| GO Biological Process | Positive regulation of peptidyl-tyrosine phosphorylation |
| GO Biological Process | Regulation of proteolysis |
| GO Biological Process | Cellular response to interferon-gamma |
| GO Biological Process | Apoptotic signaling pathway |
| GO Biological Process | Regulation of cell-cell adhesion |
| GO Biological Process | Regulation of protein phosphorylation |
| GO Biological Process | Regulation of developmental process |
| GO Biological Process | Positive regulation of intracellular signal transduction |
| GO Biological Process | Regulation of endopeptidase activity |
| GO Biological Process | Positive regulation of cytokine production involved in immune response |
| GO Biological Process | Regulation of response to cytokine stimulus |
| GO Biological Process | Regulation of interferon-gamma production |
| GO Biological Process | Activation of cysteine-type endopeptidase activity involved in apoptotic process |
| GO Biological Process | Regulation of interleukin-8 production |
| GO Biological Process | Defense response to Gram-positive bacterium |
| GO Biological Process | Regulation of adaptive immune response based on somatic recombination of immune receptors built from immunoglobulin superfamily domains |
| GO Biological Process | Regulation of ERK1 and ERK2 cascade |
| GO Biological Process | Immune effector process |
| GO Biological Process | Regulation of T cell activation |
| GO Biological Process | Positive regulation of protein phosphorylation |
| GO Biological Process | Response to organic cyclic compound |
| GO Biological Process | Leukocyte activation involved in immune response |
| GO Biological Process | Leukocyte migration |
| GO Biological Process | Cellular response to interleukin-1 |
| GO Biological Process | Positive regulation of cellular metabolic process |
| GO Biological Process | Negative regulation of signal transduction |
| GO Biological Process | Regulation of T cell cytokine production |
| GO Biological Process | Regulation of smooth muscle cell proliferation |
| GO Biological Process | Negative regulation of cellular process |
| GO Biological Process | Positive regulation of cell-cell adhesion |
| GO Biological Process | Lipopolysaccharide-mediated signaling pathway |
| GO Biological Process | Regulation of phosphate metabolic process |
| GO Biological Process | Regulation of hydrolase activity |
| GO Biological Process | Negative regulation of biological process |
| GO Biological Process | Regulation of intracellular signal transduction |
| GO Biological Process | Negative regulation of cell differentiation |
| GO Biological Process | Positive regulation of leukocyte migration |
| GO Biological Process | Positive regulation of phosphate metabolic process |
| GO Biological Process | Regulation of cytokine-mediated signaling pathway |
| GO Biological Process | Positive regulation of leukocyte cell-cell adhesion |
| GO Biological Process | Positive regulation of NF-kappaB transcription factor activity |
| GO Biological Process | Positive regulation of developmental process |
| GO Biological Process | Regulation of immunoglobulin production |
| GO Biological Process | Positive regulation of adaptive immune response |
| GO Biological Process | Positive regulation of cell migration |
| GO Biological Process | Regulation of cellular process |
| GO Biological Process | Regulation of lymphocyte mediated immunity |
| GO Biological Process | Positive regulation of smooth muscle cell proliferation |
| GO Biological Process | Regulation of biological process |
| GO Biological Process | Positive regulation of immunoglobulin production |
| GO Biological Process | Cellular response to virus |
| GO Biological Process | Positive regulation of leukocyte differentiation |
| GO Biological Process | Regulation of MAPK cascade |
| GO Biological Process | Regulation of cell adhesion |
| GO Biological Process | Regulation of cytokine production involved in inflammatory response |
| GO Biological Process | Positive regulation of biosynthetic process |
| GO Biological Process | Regulation of cellular metabolic process |
| GO Biological Process | Positive regulation of T cell activation |
| GO Biological Process | Regulation of neuron death |
| GO Biological Process | Myeloid leukocyte migration |
| GO Biological Process | Positive regulation of cell death |
| GO Biological Process | Regulation of cell migration |
| GO Biological Process | Regulation of nitrogen compound metabolic process |
| GO Biological Process | Regulation of type 2 immune response |
| GO Biological Process | Positive regulation of MHC class II biosynthetic process |
| GO Biological Process | Positive regulation of neuroinflammatory response |
| GO Biological Process | Leukocyte activation involved in inflammatory response |
| GO Biological Process | Regulation of neuroinflammatory response |
| GO Biological Process | Macrophage activation |
| GO Biological Process | Granulocyte migration |
| GO Biological Process | Leukocyte chemotaxis |
| GO Biological Process | Positive regulation of programmed cell death |
| GO Biological Process | Positive regulation of interleukin-8 production |
| GO Biological Process | Glial cell activation |
| GO Biological Process | Regulation of primary metabolic process |
| GO Biological Process | Positive regulation of cell differentiation |
| GO Biological Process | Cellular response to mechanical stimulus |
| GO Biological Process | Positive regulation of macromolecule biosynthetic process |
| GO Biological Process | Inflammasome complex assembly |
| GO Biological Process | Regulation of biosynthetic process |
| GO Biological Process | Positive regulation of interferon-gamma production |
| GO Biological Process | Positive regulation of MAPK cascade |
| GO Biological Process | Neutrophil chemotaxis |
| GO Biological Process | Leukocyte differentiation |
| GO Biological Process | Negative regulation of macromolecule metabolic process |
| GO Biological Process | Cellular response to abiotic stimulus |
| GO Biological Process | Negative regulation of cell population proliferation |
| GO Biological Process | Positive regulation of apoptotic process |
| GO Biological Process | Positive regulation of cellular biosynthetic process |
| GO Biological Process | Positive regulation of I-kappaB kinase/NF-kappaB signaling |
| GO Biological Process | Positive regulation of T cell cytokine production |
| GO Biological Process | Negative regulation of NF-kappaB transcription factor activity |
| GO Biological Process | Positive regulation of leukocyte mediated immunity |
| GO Biological Process | Regulation of interleukin-23 production |
| GO Biological Process | Regulation of cellular biosynthetic process |
| GO Biological Process | Positive regulation of B cell activation |
| GO Biological Process | Response to tumor necrosis factor |
| GO Biological Process | Humoral immune response |
| GO Biological Process | Negative regulation of molecular function |
| GO Biological Process | Regulation of macrophage activation |
| GO Biological Process | Regulation of macromolecule biosynthetic process |
| GO Biological Process | Regulation of lymphocyte differentiation |
| GO Biological Process | Regulation of response to biotic stimulus |
| GO Biological Process | Regulation of interleukin-18 production |
| GO Biological Process | Microglial cell activation |
| GO Biological Process | Response to mechanical stimulus |
| GO Biological Process | Regulation of T-helper 2 cell cytokine production |
| GO Biological Process | Positive regulation of T cell proliferation |
| GO Biological Process | Positive regulation of adaptive immune response based on somatic recombination of immune receptors built from immunoglobulin superfamily domains |
| GO Biological Process | Regulation of B cell proliferation |
| GO Biological Process | Extrinsic apoptotic signaling pathway |
| GO Biological Process | Positive regulation of lymphocyte mediated immunity |
| GO Biological Process | Response to abiotic stimulus |
| GO Biological Process | Cellular response to external stimulus |
| GO Biological Process | Negative regulation of DNA-binding transcription factor activity |
| GO Biological Process | Cellular response to tumor necrosis factor |
| GO Biological Process | Positive regulation of interleukin-10 production |
| GO Biological Process | Positive regulation of mononuclear cell migration |
| GO Biological Process | Regulation of interleukin-17 production |
| GO Biological Process | Regulation of transport |
| GO Biological Process | Negative regulation of immune effector process |
| GO Biological Process | Regulation of myeloid leukocyte differentiation |
| GO Biological Process | Regulation of mononuclear cell migration |
| GO Biological Process | Positive regulation of type 2 immune response |
| GO Biological Process | Positive regulation of nitric-oxide synthase biosynthetic process |
| GO Biological Process | Positive regulation of stress-activated MAPK cascade |
| GO Biological Process | Regulation of T cell proliferation |
| GO Biological Process | Regulation of leukocyte chemotaxis |
| GO Biological Process | Acute inflammatory response |
| GO Biological Process | Negative regulation of immune system process |
| GO Biological Process | Chemokine-mediated signaling pathway |
| GO Biological Process | Positive regulation of B cell proliferation |
| GO Biological Process | Positive regulation of receptor signaling pathway via JAK-STAT |
| GO Biological Process | Hemopoiesis |
| GO Biological Process | Activation of cysteine-type endopeptidase activity |
| GO Biological Process | Negative regulation of production of molecular mediator of immune response |
| GO Biological Process | Negative regulation of I-kappaB kinase/NF-kappaB signaling |
| GO Biological Process | Regulation of biological quality |
| GO Biological Process | Positive regulation of transport |
| GO Biological Process | Regulation of interleukin-13 production |
| GO Biological Process | Regulation of cellular catabolic process |
| GO Biological Process | Negative regulation of interleukin-6 production |
| GO Biological Process | Intracellular signal transduction |
| GO Biological Process | Regulation of transcription, DNA-templated |
| GO Biological Process | Positive regulation of nucleobase-containing compound metabolic process |
| GO Biological Process | Positive regulation of ion transport |
| GO Biological Process | Regulation of angiogenesis |
| GO Biological Process | Negative regulation of inflammatory response |
| GO Biological Process | Positive regulation of ERK1 and ERK2 cascade |
| GO Biological Process | Regulation of myeloid cell differentiation |
| GO Biological Process | Positive regulation of interleukin-23 production |
| GO Biological Process | Positive regulation of cytokine production involved in inflammatory response |
| GO Biological Process | Positive regulation of JNK cascade |
| GO Biological Process | Regulation of localization |
| GO Biological Process | Defense response to Gram-negative bacterium |
| GO Biological Process | MAPK cascade |
| GO Biological Process | Negative regulation of response to external stimulus |
| GO Biological Process | Positive regulation of leukocyte chemotaxis |
| GO Biological Process | Regulation of natural killer cell chemotaxis |
| GO Biological Process | Regulation of catabolic process |
| GO Biological Process | Regulation of NIK/NF-kappaB signaling |
| GO Biological Process | Positive regulation of macrophage activation |
| GO Biological Process | Regulation of lymphocyte chemotaxis |
| GO Biological Process | Regulation of nucleobase-containing compound metabolic process |
| GO Biological Process | Cellular response to organic cyclic compound |
| GO Biological Process | Regulation of epithelial cell apoptotic process |
| GO Biological Process | Negative regulation of intracellular signal transduction |
| GO Biological Process | Positive regulation of peptidyl-serine phosphorylation |
| GO Biological Process | Regulation of interleukin-12 production |
| GO Biological Process | Regulation of chronic inflammatory response |
| GO Biological Process | Natural killer cell activation |
| GO Biological Process | Regulation of protein secretion |
| GO Biological Process | Positive regulation of NIK/NF-kappaB signaling |
| GO Biological Process | Mononuclear cell migration |
| GO Biological Process | Regulation of T cell differentiation |
| GO Biological Process | Response to nitrogen compound |
| GO Biological Process | Regulation of signaling receptor activity |
| GO Biological Process | Granulocyte activation |
| GO Biological Process | Regulation of lymphocyte migration |
| GO Biological Process | Positive regulation of transcription, DNA-templated |
| GO Biological Process | Regulation of osteoclast differentiation |
| GO Biological Process | Regulation of autophagy |
| GO Biological Process | Negative regulation of immune response |
| GO Biological Process | Negative regulation of cytokine production involved in immune response |
| GO Biological Process | Negative regulation of interleukin-1 production |
| GO Biological Process | Type 2 immune response |
| GO Biological Process | Positive regulation of response to biotic stimulus |
| GO Biological Process | B cell activation |
| GO Biological Process | Positive regulation of transcription by RNA polymerase II |
| GO Biological Process | Response to ethanol |
| GO Biological Process | Adaptive immune response |
| GO Biological Process | Positive regulation of protein-containing complex assembly |
| GO Biological Process | Cell killing |
| GO Biological Process | Positive regulation of lymphocyte differentiation |
| GO Biological Process | Inflammatory response to antigenic stimulus |
| GO Biological Process | Regulation of ion transport |
| GO Biological Process | Positive regulation of interleukin-13 production |
| GO Biological Process | Multicellular organismal homeostasis |
| GO Biological Process | Myeloid leukocyte differentiation |
| GO Biological Process | Protein processing |
| GO Biological Process | Acute-phase response |
| GO Biological Process | Type I interferon signaling pathway |
| GO Biological Process | Positive regulation of kinase activity |
| GO Biological Process | Monocyte chemotaxis |
| GO Biological Process | Positive regulation of lymphocyte migration |
| GO Biological Process | Negative regulation of cell death |
| GO Biological Process | Positive regulation of cellular component biogenesis |
| GO Biological Process | Positive regulation of innate immune response |
| GO Biological Process | Regulation of secretion |
| GO Biological Process | Leukocyte proliferation |
| GO Biological Process | Eosinophil chemotaxis |
| GO Biological Process | Positive regulation of calcidiol 1-monooxygenase activity |
| GO Biological Process | Positive regulation of neuron death |
| GO Biological Process | Response to endogenous stimulus |
| GO Biological Process | Positive regulation of cellular component organization |
| GO Biological Process | Cytokine production |
| GO Biological Process | Regulation of extrinsic apoptotic signaling pathway |
| GO Biological Process | Regulation of cellular respiration |
| GO Biological Process | Positive regulation of cellular catabolic process |
| GO Biological Process | Regulation of protein-containing complex assembly |
| GO Biological Process | Negative regulation of programmed cell death |
| GO Biological Process | Cellular process |
| GO Biological Process | Positive regulation of protein kinase activity |
| GO Biological Process | Regulation of acute inflammatory response |
| GO Biological Process | Regulation of kinase activity |
| GO Biological Process | Negative regulation of extrinsic apoptotic signaling pathway |
| GO Biological Process | Receptor signaling pathway via JAK-STAT |
| GO Biological Process | Lymphocyte chemotaxis |
| GO Biological Process | Regulation of endothelial cell apoptotic process |
| GO Biological Process | Regulation of NLRP3 inflammasome complex assembly |
| GO Biological Process | Regulation of innate immune response |
| GO Biological Process | Regulation of protein kinase activity |
| GO Biological Process | System development |
| GO Biological Process | Positive regulation of myeloid cell differentiation |
| GO Biological Process | B cell proliferation |
| GO Biological Process | Protein kinase B signaling |
| GO Biological Process | Positive regulation of peptidyl-serine phosphorylation of STAT protein |
| GO Biological Process | Positive regulation of angiogenesis |
| GO Biological Process | Regulation of protein kinase B signaling |
| GO Biological Process | Regulation of tumor necrosis factor-mediated signaling pathway |
| GO Biological Process | Regulation of CD4-positive, alpha-beta T cell differentiation |
| GO Biological Process | Regulation of type I interferon production |
| GO Biological Process | Regulation of cellular component organization |
| GO Biological Process | Antiviral innate immune response |
| GO Biological Process | Regulation of secretion by cell |
| GO Biological Process | Response to alcohol |
| GO Biological Process | Negative regulation of leukocyte differentiation |
| GO Biological Process | Negative regulation of adaptive immune response based on somatic recombination of immune receptors built from immunoglobulin superfamily domains |
| GO Biological Process | Regulation of cellular component biogenesis |
| GO Biological Process | Animal organ development |
| GO Biological Process | Biological process involved in interaction with symbiont |
| GO Biological Process | Positive regulation of myeloid leukocyte differentiation |
| GO Biological Process | Biological process involved in symbiotic interaction |
| GO Biological Process | Lymphocyte activation |
| GO Biological Process | Regulation of nitric oxide biosynthetic process |
| GO Biological Process | Regulation of heterotypic cell-cell adhesion |
| GO Biological Process | Myeloid cell differentiation |
| GO Biological Process | Cell population proliferation |
| GO Biological Process | Regulation of cellular response to stress |
| GO Biological Process | Myeloid cell activation involved in immune response |
| GO Biological Process | Regulation of membrane protein ectodomain proteolysis |
| GO Biological Process | Positive regulation of macrophage cytokine production |
| GO Biological Process | Positive regulation of T cell differentiation |
| GO Biological Process | Regulation of apoptotic signaling pathway |
| GO Biological Process | Positive regulation of response to cytokine stimulus |
| GO Biological Process | Osmosensory signaling pathway |
| GO Biological Process | Positive regulation of natural killer cell chemotaxis |
| GO Biological Process | Activation of innate immune response |
| GO Biological Process | Positive regulation of interleukin-17 production |
| GO Biological Process | Negative regulation of signaling receptor activity |
| GO Biological Process | Protein homooligomerization |
| GO Biological Process | Lymphocyte proliferation |
| GO Biological Process | Negative regulation of apoptotic process |
| GO Biological Process | I-KappaB kinase/NF-kappaB signaling |
| GO Biological Process | Macrophage differentiation |
| GO Biological Process | Neutrophil activation |
| GO Biological Process | Regulation of protein serine/threonine kinase activity |
| GO Biological Process | Wound healing involved in inflammatory response |
| GO Biological Process | Vascular endothelial growth factor production |
| GO Biological Process | Liver regeneration |
| GO Biological Process | Lymphocyte activation involved in immune response |
| GO Biological Process | B cell differentiation |
| GO Biological Process | Cell motility |
| GO Biological Process | Negative regulation of ERK1 and ERK2 cascade |
| GO Biological Process | Regulation of T-helper 1 type immune response |
| GO Biological Process | Positive regulation of interleukin-18 production |
| GO Biological Process | Homeostatic process |
| GO Biological Process | Regulation of MHC class I biosynthetic process |
| GO Biological Process | Positive regulation of calcium ion transport |
| GO Biological Process | Positive regulation of T-helper 1 cell cytokine production |
| GO Biological Process | Regulation of interleukin-1-mediated signaling pathway |
| GO Biological Process | Negative regulation of cytokine-mediated signaling pathway |
| GO Biological Process | T Cell activation involved in immune response |
| GO Biological Process | Intrinsic apoptotic signaling pathway in response to DNA damage |
| GO Biological Process | Positive regulation of phagocytosis |
| GO Biological Process | Regulation of endothelial cell proliferation |
| GO Biological Process | Positive regulation of phosphatidylinositol 3-kinase signaling |
| GO Biological Process | Self proteolysis |
| GO Biological Process | Positive regulation of T-helper 2 cell cytokine production |
| GO Biological Process | Response to organonitrogen compound |
| GO Biological Process | Astrocyte development |
| GO Biological Process | Regulation of sensory perception of pain |
| GO Biological Process | Extrinsic apoptotic signaling pathway in absence of ligand |
| GO Biological Process | Negative regulation of extrinsic apoptotic signaling pathway in absence of ligand |
| GO Biological Process | Positive regulation of autophagy |
| GO Biological Process | Positive regulation of small molecule metabolic process |
| GO Biological Process | Positive regulation of protein serine/threonine kinase activity |
| GO Biological Process | Mononuclear cell differentiation |
| GO Biological Process | Regulation of epithelial cell differentiation |
| GO Biological Process | Negative regulation of apoptotic signaling pathway |
| GO Biological Process | Regulation of insulin secretion |
| GO Biological Process | Anatomical structure development |
| GO Biological Process | Positive regulation of podosome assembly |
| GO Biological Process | Regulation of T-helper cell differentiation |
| GO Biological Process | Positive regulation of interleukin-12 production |
| GO Biological Process | Regulation of transmembrane transport |
| GO Biological Process | Gliogenesis |
| GO Biological Process | Positive regulation of nitric oxide biosynthetic process |
| GO Biological Process | Negative regulation of interferon-gamma production |
| GO Biological Process | Intrinsic apoptotic signaling pathway |
| GO Biological Process | Response to corticosteroid |
| GO Biological Process | Macrophage chemotaxis |
| GO Biological Process | Cellular response to interleukin-17 |
| GO Biological Process | Regulation of lipid localization |
| GO Biological Process | Regulation of defense response to virus by host |
| GO Biological Process | Negative regulation of MAPK cascade |
| GO Biological Process | Cellular response to stress |
| GO Biological Process | Negative regulation of T cell differentiation |
| GO Biological Process | Positive regulation of cellular respiration |
| GO Biological Process | Regulation of neutrophil migration |
| GO Biological Process | Negative regulation of epithelial cell differentiation |
| GO Biological Process | Regulation of oxidoreductase activity |
| GO Biological Process | Reactive oxygen species metabolic process |
| GO Biological Process | Regulation of calcium ion transport |
| GO Biological Process | Positive regulation of granulocyte macrophage colony-stimulating factor production |
| GO Biological Process | Positive regulation of heterotypic cell-cell adhesion |
| GO Biological Process | Negative regulation by host of viral transcription |
| GO Biological Process | Cell-cell signaling |
| GO Biological Process | Regulation of lipid storage |
| GO Biological Process | Regulation of anatomical structure morphogenesis |
| GO Biological Process | Regulation of monooxygenase activity |
| GO Biological Process | Epithelial cell apoptotic process |
| GO Biological Process | Lymphocyte differentiation |
| GO Biological Process | Negative regulation of smooth muscle cell proliferation |
| GO Biological Process | Astrocyte activation |
| GO Biological Process | Positive regulation of membrane protein ectodomain proteolysis |
| GO Biological Process | Positive regulation of protein kinase B signaling |
| GO Biological Process | Negative regulation of cell-cell adhesion |
| GO Biological Process | Positive regulation of MAP kinase activity |
| GO Biological Process | Positive regulation of glial cell proliferation |
| GO Biological Process | Regulation of chemokine (C-X-C motif) ligand 2 production |
| GO Biological Process | Negative regulation of leukocyte activation |
| GO Biological Process | Positive regulation of cytokine-mediated signaling pathway |
| GO Biological Process | JNK cascade |
| GO Biological Process | Regulation of metal ion transport |
| GO Biological Process | Myeloid leukocyte mediated immunity |
| GO Biological Process | Response to inorganic substance |
| GO Biological Process | T Cell activation |
| GO Biological Process | Regulation of amyloid-beta clearance |
| GO Biological Process | Negative regulation of epithelial cell apoptotic process |
| GO Biological Process | Toll-like receptor signaling pathway |
| GO Biological Process | Maintenance of gastrointestinal epithelium |
| GO Biological Process | Regulation of interleukin-5 production |
| GO Biological Process | Negative regulation of tumor necrosis factor production |
| GO Biological Process | Positive regulation of DNA metabolic process |
| GO Biological Process | Interleukin-1-mediated signaling pathway |
| GO Biological Process | Negative regulation of phosphate metabolic process |
| GO Biological Process | MyD88-Dependent toll-like receptor signaling pathway |
| GO Biological Process | Regulation of immunoglobulin mediated immune response |
| GO Biological Process | Negative regulation of lipid storage |
| GO Biological Process | Positive regulation of type I interferon production |
| GO Biological Process | Positive regulation of vitamin D biosynthetic process |
| GO Biological Process | Pyroptosome complex assembly |
| GO Biological Process | Positive regulation of humoral immune response |
| GO Biological Process | Positive regulation of steroid biosynthetic process |
| GO Biological Process | Regulation of neuron apoptotic process |
| GO Biological Process | Negative regulation of T cell activation |
| GO Biological Process | Regulation of transcription by RNA polymerase II |
| GO Biological Process | Positive regulation of gliogenesis |
| GO Biological Process | Angiogenesis |
| GO Biological Process | Negative regulation of neuron death |
| GO Biological Process | Negative regulation of protein phosphorylation |
| GO Biological Process | Negative regulation of protein metabolic process |
| GO Biological Process | Multicellular organismal process |
| GO Biological Process | Negative regulation of chemokine production |
| GO Biological Process | Positive regulation of reactive oxygen species metabolic process |
| GO Biological Process | Regulation of steroid biosynthetic process |
| GO Biological Process | Regulation of viral life cycle |
| GO Biological Process | Myeloid dendritic cell activation |
| GO Biological Process | Neutrophil mediated immunity |
| GO Biological Process | Response to toxic substance |
| GO Biological Process | Killing of cells of another organism |
| GO Biological Process | Wound healing |
| GO Biological Process | Fever generation |
| GO Biological Process | Chronic inflammatory response to antigenic stimulus |
| GO Biological Process | Regulation of chronic inflammatory response to antigenic stimulus |
| GO Biological Process | Response to fungus |
| GO Biological Process | interleukin-33-mediated signaling pathway |
| GO Biological Process | Positive regulation of plasma cell differentiation |
| GO Biological Process | Regulation of oxidative stress-induced cell death |
| GO Biological Process | Negative regulation of complement-dependent cytotoxicity |
| GO Biological Process | Regulation of reactive oxygen species metabolic process |
| GO Biological Process | Positive regulation of interleukin-18-mediated signaling pathway |
| GO Biological Process | Natural killer cell activation involved in immune response |
| GO Biological Process | Regulation of nervous system process |
| GO Biological Process | Regulation of organic acid transport |
| GO Biological Process | Positive regulation of osteoclast differentiation |
| GO Biological Process | Response to glucocorticoid |
| GO Biological Process | Tissue homeostasis |
| GO Biological Process | Positive regulation of acute inflammatory response |
| GO Biological Process | Positive regulation of interleukin-4 production |
| GO Biological Process | Positive regulation of activated T cell proliferation |
| GO Biological Process | Regulation of myoblast differentiation |
| GO Biological Process | Negative regu4lation of myoblast differentiation |
| GO Biological Process | Modulation by host of symbiont process |
| GO Biological Process | Response to osmotic stress |
| GO Biological Process | Positive regulation of vascular endothelial growth factor production |
| GO Biological Process | Negative regulation of interleukin-1 beta production |
| GO Biological Process | Killing by host of symbiont cells |
| GO Biological Process | Smooth muscle adaptation |
| GO Biological Process | Cellular homeostasis |
| GO Biological Process | Sequestering of triglyceride |
| GO Biological Process | NLRP3 inflammasome complex assembly |
| GO Biological Process | Negative regulation of T-helper 2 cell cytokine production |
| GO Biological Process | Regulation of macrophage derived foam cell differentiation |
| GO Biological Process | Regulation of interferon-alpha production |
| GO Biological Process | Regulation of oxidative stress-induced neuron death |
| GO Biological Process | Positive regulation of leukocyte apoptotic process |
| GO Biological Process | Negative regulation of endothelial cell apoptotic process |
| GO Biological Process | Regulation of ion transmembrane transport |
| GO Biological Process | Regulation of mast cell degranulation |
| GO Biological Process | Regulation of viral genome replication |
| GO Biological Process | Regulation of B cell differentiation |
| GO Biological Process | Positive regulation of lipid biosynthetic process |
| GO Biological Process | Negative regulation of protein modification process |
| GO Biological Process | Positive regulation of CD4-positive, alpha-beta T cell differentiation |
| GO Biological Process | Negative regulation of osteoclast differentiation |
| GO Biological Process | Negative regulation of chronic inflammatory response |
| GO Biological Process | Cell differentiation |
| GO Biological Process | Intestinal epithelial structure maintenance |
| GO Biological Process | Negative regulation of interleukin-1-mediated signaling pathway |
| GO Biological Process | Positive regulation of defense response to virus by host |
| GO Biological Process | Modulation of process of another organism |
| GO Biological Process | Cellular response to chemical stress |
| GO Biological Process | Positive regulation of cation transmembrane transport |
| GO Biological Process | Regulation of endothelial cell differentiation |
| GO Biological Process | Regulation of lipid biosynthetic process |
| GO Biological Process | T Cell migration |
| GO Biological Process | Positive regulation of neutrophil migration |
| GO Biological Process | Positive regulation of T cell migration |
| GO Biological Process | Regulation of hormone levels |
| GO Biological Process | Regulation of anion transport |
| GO Biological Process | Positive regulation of nervous system development |
| GO Biological Process | Response to hypoxia |
| GO Biological Process | Negative regulation of viral process |
| GO Biological Process | Regulation of leukocyte apoptotic process |
| GO Biological Process | Detection of biotic stimulus |
| GO Biological Process | Hydrogen peroxide metabolic process |
| GO Biological Process | Peptidyl-cysteine S-nitrosylation |
| GO Biological Process | Positive regulation of immature T cell proliferation in thymus |
| GO Biological Process | Positive regulation of MHC class I biosynthetic process |
| GO Biological Process | Negative regulation of nitrogen compound metabolic process |
| GO Biological Process | Positive regulation of killing of cells of another organism |
| GO Biological Process | Positive regulation of secretion by cell |
| GO Biological Process | Positive regulation of NMDA glutamate receptor activity |
| GO Biological Process | Regulation of chemokine (C-X-C motif) ligand 1 production |
| GO Biological Process | Regulation of isotype switching |
| GO Biological Process | Regulation of DNA metabolic process |
| GO Biological Process | Regulation of protein localization |
| GO Biological Process | Negative regulation of leukocyte proliferation |
| GO Biological Process | Regulation of vesicle-mediated transport |
| GO Biological Process | Regulation of nitric-oxide synthase activity |
| GO Biological Process | Cellular response to amyloid-beta |
| GO Biological Process | Regulation of cell killing |
| GO Biological Process | Positive regulation of fever generation |
| GO Biological Process | Astrocyte cell migration |
| GO Biological Process | Positive regulation of T-helper 2 cell differentiation |
| GO Biological Process | Negative regulation of NLRP3 inflammasome complex assembly |
| GO Biological Process | Organ or tissue specific immune response |
| GO Biological Process | Superoxide metabolic process |
| GO Biological Process | Response to xenobiotic stimulus |
| GO Biological Process | Regulation of inflammatory response to antigenic stimulus |
| GO Biological Process | Positive regulation of immunoglobulin mediated immune response |
| GO Biological Process | Positive regulation of calcium-mediated signaling |
| GO Biological Process | Positive regulation of epithelial cell apoptotic process |
| GO Biological Process | Positive regulation of protein transport |
| GO Biological Process | Negative regulation of endothelial cell proliferation |
| GO Biological Process | Positive regulation of cell development |
| GO Biological Process | Negative regulation of cellular metabolic process |
| GO Biological Process | Organonitrogen compound metabolic process |
| GO Biological Process | Proteolysis |
| GO Biological Process | Regulation of cell adhesion molecule production |
| GO Biological Process | Positive regulation of oxidative stress-induced neuron death |
| GO Biological Process | Response to peptide |
| GO Biological Process | Regulation of cell junction assembly |
| GO Biological Process | Positive regulation of mitotic nuclear division |
| GO Biological Process | Antimicrobial humoral immune response mediated by antimicrobial peptide |
| GO Biological Process | Positive regulation of organic acid transport |
| GO Biological Process | Positive regulation of signaling receptor activity |
| GO Biological Process | Neutrophil-mediated killing of bacterium |
| GO Biological Process | Negative regulation of amyloid-beta clearance |
| GO Biological Process | Cellular response to heat |
| GO Biological Process | Response to nicotine |
| GO Biological Process | Positive regulation of purine nucleotide metabolic process |
| GO Biological Process | Leukocyte mediated immunity |
| GO Biological Process | Cellular response to endogenous stimulus |
| GO Biological Process | Negative regulation of leukocyte migration |
| GO Biological Process | Icosanoid biosynthetic process |
| GO Biological Process | Response to hormone |
| GO Biological Process | Negative regulation of catabolic process |
| GO Biological Process | Response to exogenous dsRNA |
| GO Biological Process | Positive regulation of anion transport |
| GO Biological Process | Regulation of prostaglandin biosynthetic process |
| GO Biological Process | Response to cobalt ion |
| GO Biological Process | Negative regulation of heterotypic cell-cell adhesion |
| GO Biological Process | Positive regulation of isotype switching to IgG isotypes |
| GO Biological Process | Regulation of gap junction assembly |
| GO Biological Process | Positive regulation of chemokine (C-X-C motif) ligand 2 production |
| GO Biological Process | Negative regulation of fat cell differentiation |
| GO Biological Process | Regulation of granulocyte chemotaxis |
| GO Biological Process | Positive regulation of interleukin-5 production |
| GO Biological Process | Positive regulation of tissue remodeling |
| GO Biological Process | Endothelial cell apoptotic process |
| GO Biological Process | Cellular response to oxidized low-density lipoprotein particle stimulus |
| GO Biological Process | Negative regulation of T-helper 17 cell differentiation |
| GO Biological Process | Positive regulation of neuron apoptotic process |
| GO Biological Process | Positive regulation of neurogenesis |
| GO Biological Process | B cell activation involved in immune response |
| GO Biological Process | Regulation of epithelial cell migration |
| GO Biological Process | Positive regulation of apoptotic signaling pathway |
| GO Biological Process | Leukocyte mediated cytotoxicity |
| GO Biological Process | Leukocyte cell-cell adhesion |
| GO Biological Process | Tumor necrosis factor-mediated signaling pathway |
| GO Biological Process | Negative regulation of viral genome replication |
| GO Biological Process | Regulation of DNA recombination |
| GO Biological Process | I-KappaB phosphorylation |
| GO Biological Process | Positive regulation of NLRP3 inflammasome complex assembly |
| GO Biological Process | Positive regulation of tumor necrosis factor-mediated signaling pathway |
| GO Biological Process | Response to extracellular stimulus |
| GO Biological Process | Circulatory system process |
| GO Biological Process | Regulation of mitotic cell cycle |
| GO Biological Process | Regulation of interferon-beta production |
| GO Biological Process | Response to metal ion |
| GO Biological Process | Negative regulation of acute inflammatory response |
| GO Biological Process | Response to peptidoglycan |
| GO Biological Process | Protein metabolic process |
| GO Biological Process | Regulation of cell development |
| GO Biological Process | Regulation of protein catabolic process |
| GO Biological Process | Positive regulation of organelle organization |
| GO Biological Process | Response to oxidative stress |
| GO Biological Process | Regulation of vascular associated smooth muscle cell proliferation |
| GO Biological Process | Positive regulation of prostaglandin secretion |
| GO Biological Process | Secretion |
| GO Biological Process | Regulation of lymphocyte apoptotic process |
| GO Biological Process | Regulation of cation transmembrane transport |
| GO Biological Process | Regulation of cold-induced thermogenesis |
| GO Biological Process | Response to nutrient |
| GO Biological Process | Negative regulation of interleukin-17 production |
| GO Biological Process | Positive regulation of mast cell degranulation |
| GO Biological Process | Positive regulation of calcium ion import |
| GO Biological Process | Reactive oxygen species biosynthetic process |
| GO Biological Process | Regulation of muscle cell apoptotic process |
| GO Biological Process | Negative regulation of leukocyte mediated immunity |
| GO Biological Process | Response to activity |
| GO Biological Process | Regulation of dendritic cell cytokine production |
| GO Biological Process | T Cell chemotaxis |
| GO Biological Process | Regulation of establishment of endothelial barrier |
| GO Biological Process | Negative regulation of miRNA maturation |
| GO Biological Process | Regulation of T cell chemotaxis |
| GO Biological Process | Regulation of amino acid import across plasma membrane |
| GO Biological Process | Positive regulation of homotypic cell-cell adhesion |
| GO Biological Process | Blood circulation |
| GO Biological Process | Regulation of macroautophagy |
| GO Biological Process | Positive regulation of DNA recombination |
| GO Biological Process | Positive regulation of macrophage derived foam cell differentiation |
| GO Biological Process | Negative regulation of glucose transmembrane transport |
| GO Biological Process | Negative regulation of interleukin-12 production |
| GO Biological Process | Ectopic germ cell programmed cell death |
| GO Biological Process | Regulation of microglial cell activation |
| GO Biological Process | Positive regulation of macroautophagy |
| GO Biological Process | Regulation of transmembrane transporter activity |
| GO Biological Process | Circulatory system development |
| GO Biological Process | Regulation of tissue remodeling |
| GO Biological Process | Regulation of system process |
| GO Biological Process | Macrophage activation involved in immune response |
| GO Biological Process | Negative regulation of interleukin-8 production |
| GO Biological Process | Positive regulation of lymphocyte apoptotic process |
| GO Biological Process | Response to reactive oxygen species |
| GO Biological Process | Adaptive immune response based on somatic recombination of immune receptors built from immunoglobulin superfamily domains |
| GO Biological Process | Negative regulation of cell migration |
| GO Biological Process | Negative regulation of nervous system process |
| GO Biological Process | Macromolecule metabolic process |
| GO Biological Process | Protein-containing complex assembly |
| GO Biological Process | Necroptotic process |
| GO Biological Process | Detection of other organisms |
| GO Biological Process | Regulation of bicellular tight junction assembly |
| GO Biological Process | Response to temperature stimulus |
| GO Biological Process | Positive regulation of cellular amide metabolic process |
| GO Biological Process | Nitric oxide biosynthetic process |
| GO Biological Process | Myeloid dendritic cell differentiation |
| GO Biological Process | Regulation of myoblast fusion |
| GO Biological Process | Positive regulation of amyloid-beta formation |
| GO Biological Process | Cellular response to fibroblast growth factor stimulus |
| GO Biological Process | Regulation of B cell apoptotic process |
| GO Biological Process | Negative regulation of ATP-dependent activity |
| GO Biological Process | T-Helper 1 type immune response |
| GO Biological Process | Superoxide anion generation |
| GO Biological Process | Negative regulation of cell development |
| GO Biological Process | Negative regulation of catalytic activity |
| GO Biological Process | Embryonic placenta development |
| GO Biological Process | Regulation of smooth muscle cell apoptotic process |
| GO Biological Process | Cellular response to interferon-beta |
| GO Biological Process | Positive regulation of regulatory T cell differentiation |
| GO Biological Process | Negative regulation of heart contraction |
| GO Biological Process | Positive regulation of exocytosis |
| GO Biological Process | Positive regulation of monocyte chemotaxis |
| GO Biological Process | Positive regulation of leukocyte adhesion to vascular endothelial cell |
| GO Biological Process | Cell adhesion |
| GO Biological Process | Positive regulation of lipid transport |
| GO Biological Process | Granulocyte differentiation |
| GO Biological Process | Negative regulation of cytokine production involved in inflammatory response |
| GO Biological Process | Regulation of blood vessel endothelial cell migration |
| GO Biological Process | Neuron apoptotic process |
| GO Biological Process | Regulation of blood pressure |
| GO Biological Process | Response to nutrient levels |
| GO Biological Process | Positive regulation of interferon-alpha production |
| GO Biological Process | Dendritic cell migration |
| GO Biological Process | Hydrogen peroxide catabolic process |
| GO Biological Process | Negative regulation of vascular associated smooth muscle cell proliferation |
| GO Biological Process | Negative regulation of mononuclear cell proliferation |
| GO Cellular Component | Inflammasome complex |
| GO Cellular Component | Extracellular region |
| GO Cellular Component | Extracellular space |
| GO Cellular Component | NLRP3 inflammasome complex |
| GO Cellular Component | NLRP1 inflammasome complex |
| GO Cellular Component | AIM2 inflammasome complex |
| GO Cellular Component | IPAF inflammasome complex |
| GO Cellular Component | Caspase complex |
| GO Cellular Component | NLRP6 inflammasome complex |
| GO Molecular Function | Cytokine receptor binding |
| GO Molecular Function | Cytokine activity |
| GO Molecular Function | Signaling receptor regulator activity |
| GO Molecular Function | Signaling receptor binding |
| GO Molecular Function | Molecular function regulator activity |
| GO Molecular Function | Growth factor receptor binding |
| GO Molecular Function | Protein binding |
| GO Molecular Function | Interleukin-1 receptor binding |
| GO Molecular Function | Cysteine-type endopeptidase activity involved in apoptotic process |
| GO Molecular Function | Binding |
| GO Molecular Function | Chemokine activity |
| GO Molecular Function | Cysteine-type endopeptidase activity involved in apoptotic signaling pathway |
| GO Molecular Function | Identical protein binding |
| GO Molecular Function | Cysteine-type endopeptidase activator activity involved in apoptotic process |
| GO Molecular Function | Peptidase activator activity |
| GO Molecular Function | Growth factor activity |
| GO Molecular Function | Enzyme activator activity |
| GO Molecular Function | CCR1 chemokine receptor binding |
| GO Molecular Function | Pattern recognition receptor activity |
| GO Molecular Function | CCR5 chemokine receptor binding |
| GO Molecular Function | G-Protein-coupled receptor binding |
| GO Molecular Function | Interleukin-1 binding |
| GO Molecular Function | Heme binding |
| GO Molecular Function | Peptidase regulator activity |
| GO Molecular Function | Endopeptidase activity |
| GO Molecular Function | Protein domain specific binding |
| GO Molecular Function | Endopeptidase activator activity |
| GO Molecular Function | CCR chemokine receptor binding |
| GO Molecular Function | NAD(P)+ nucleosidase activity |
| GO Molecular Function | CARD domain binding |
| GO Molecular Function | NAD+ nucleotidase, cyclic ADP-ribose generating |
| GO Molecular Function | Type I interferon receptor binding |
| GO Molecular Function | CXCR chemokine receptor binding |
| GO Molecular Function | Cysteine-type endopeptidase activator activity |
| GO Molecular Function | Interleukin-1 receptor antagonist activity |
| GO Molecular Function | Enzyme regulator activity |
| GO Molecular Function | Protein self-association |
| GO Molecular Function | Protein dimerization activity |
| GO Molecular Function | Receptor antagonist activity |
| GO Molecular Function | Interleukin-6 receptor binding |
| GO Molecular Function | Flavin adenine dinucleotide binding |
| GO Molecular Function | Chemoattractant activity |
| GO Molecular Function | Interleukin-1 receptor activity |
| GO Molecular Function | Protein homodimerization activity |
| GO Molecular Function | Cysteine-type endopeptidase activity involved in execution phase of apoptosis |
| GO Molecular Function | Tumor necrosis factor receptor superfamily binding |

| KEGG Pathways | Salmonella infection |
| --- | --- |
| KEGG Pathways | Legionellosis |
| KEGG Pathways | Rheumatoid arthritis |
| KEGG Pathways | Inflammatory bowel disease |
| KEGG Pathways | Cytosolic DNA-sensing pathway |
| KEGG Pathways | Malaria |
| KEGG Pathways | Pathogenic Escherichia coli infection |
| KEGG Pathways | Necroptosis |
| KEGG Pathways | Viral protein interaction with cytokine and cytokine receptor |
| KEGG Pathways | Shigellosis |
| KEGG Pathways | Pertussis |
| KEGG Pathways | Measles |
| KEGG Pathways | JAK-STAT signaling pathway |
| KEGG Pathways | Human cytomegalovirus infection |
| KEGG Pathways | Amoebiasis |
