# Supplementary material for: Emphasizing the role of oxidative stress and Sirt-1/Nrf2 and TLR-4/NF-κB in Tamarix aphylla mediated neuroprotective potential in rotenone-induced Parkinson’s disease: In silico and in vivo study
Source: PLoS One. 2026 Jan 6;21(1):e0339010. doi: 10.1371/journal.pone.0339010 (PMC12774373; doi:10.1371/journal.pone.0339010)
Supplement: S18 Table — (DOCX) [file pone.0339010.s018.docx]

**Table S18. KEGG Enrichment Entry.**

| **Category** | **Description** |
| --- | --- |
| KEGG Pathways | NOD-like receptor signaling pathway |
| KEGG Pathways | Cytokine-cytokine receptor interaction |
| KEGG Pathways | Influenza A |
| KEGG Pathways | Yersinia infection |
| KEGG Pathways | Chagas disease |
| KEGG Pathways | Toll-like receptor signaling pathway |
| KEGG Pathways | IL-17 signaling pathway |
| KEGG Pathways | Tuberculosis |
| KEGG Pathways | Salmonella infection |
| KEGG Pathways | Legionellosis |
| KEGG Pathways | Rheumatoid arthritis |
| KEGG Pathways | Inflammatory bowel disease |
| KEGG Pathways | Cytosolic DNA-sensing pathway |
| KEGG Pathways | Malaria |
| KEGG Pathways | Pathogenic Escherichia coli infection |
| KEGG Pathways | Necroptosis |
| KEGG Pathways | Viral protein interaction with cytokine and cytokine receptor |
| KEGG Pathways | Shigellosis |
| KEGG Pathways | Pertussis |
| KEGG Pathways | Measles |
| KEGG Pathways | JAK-STAT signaling pathway |
| KEGG Pathways | Human cytomegalovirus infection |
| KEGG Pathways | Amoebiasis |
| KEGG Pathways | Leishmaniasis |
| KEGG Pathways | African trypanosomiasis |
| KEGG Pathways | TNF signaling pathway |
| KEGG Pathways | Hepatitis B |
| KEGG Pathways | Hematopoietic cell lineage |
| KEGG Pathways | C-Type lectin receptor signaling pathway |
| KEGG Pathways | Herpes simplex virus 1 infection |
| KEGG Pathways | Kaposi sarcoma-associated herpesvirus infection |
| KEGG Pathways | Epstein-Barr virus infection |
| KEGG Pathways | Pathways in cancer |
| KEGG Pathways | AGE-RAGE signaling pathway in diabetic complications |
| KEGG Pathways | NF-Kappa B signaling pathway |
| KEGG Pathways | Toxoplasmosis |
| KEGG Pathways | Hepatitis C |
| KEGG Pathways | Allograft rejection |
| KEGG Pathways | Graft-versus-host disease |
| KEGG Pathways | RIG-I-like receptor signaling pathway |
| KEGG Pathways | Fluid shear stress and atherosclerosis |
| KEGG Pathways | Autoimmune thyroid disease |
| KEGG Pathways | Th17 cell differentiation |
| KEGG Pathways | T Cell receptor signaling pathway |
| KEGG Pathways | HIF-1 signaling pathway |
| KEGG Pathways | Asthma |
| KEGG Pathways | Osteoclast differentiation |
| KEGG Pathways | Natural killer cell mediated cytotoxicity |
| KEGG Pathways | PI3K-Akt signaling pathway |
| KEGG Pathways | Alzheimer disease |
| KEGG Pathways | Type I diabetes mellitus |
| KEGG Pathways | Human immunodeficiency virus 1 infection |
| KEGG Pathways | Intestinal immune network for IgA production |
| KEGG Pathways | Chemokine signaling pathway |
| KEGG Pathways | Fc epsilon RI signaling pathway |
| KEGG Pathways | Non-alcoholic fatty liver disease |
| KEGG Pathways | Th1 and Th2 cell differentiation |
| KEGG Pathways | Prion disease |
| KEGG Pathways | Antifolate resistance |
| KEGG Pathways | PD-L1 expression and PD-1 checkpoint pathway in cancer |
| KEGG Pathways | Human papillomavirus infection |
| KEGG Pathways | Human T cell leukemia virus 1 infection |
| KEGG Pathways | Viral myocarditis |
| KEGG Pathways | Apoptosis |
| KEGG Pathways | Phagosome |
| KEGG Pathways | Epithelial cell signaling in Helicobacter pylori infection |
| KEGG Pathways | Cellular senescence |
| KEGG Pathways | MAPK signaling pathway |
| KEGG Pathways | Hypertrophic cardiomyopathy |
| KEGG Pathways | Systemic lupus erythematosus |
| KEGG Pathways | Apoptosis - multiple species |
| KEGG Pathways | Amyotrophic lateral sclerosis |
| KEGG Pathways | FoxO signaling pathway |
| KEGG Pathways | Ferroptosis |
