# Supplementary material for: Emphasizing the role of oxidative stress and Sirt-1/Nrf2 and TLR-4/NF-κB in Tamarix aphylla mediated neuroprotective potential in rotenone-induced Parkinson’s disease: In silico and in vivo study
Source: PLoS One. 2026 Jan 6;21(1):e0339010. doi: 10.1371/journal.pone.0339010 (PMC12774373; doi:10.1371/journal.pone.0339010)
Supplement: S19 Table — (DOCX) [file pone.0339010.s019.docx]

**Table S19. Binding Energies and RMSD of the 13 Compounds into the Active Pocket Site of the SIRT1 Catalytic Domain (PDB: 4i5i).**

| **NO** | **S (kcal/mol)** | **RMSD_refine** |
| --- | --- | --- |
| 1 | -5.27458 | 1.38488388 |
| 2 | -5.97551918 | 1.42401814 |
| 3 | -5.8754549 | 1.61728275 |
| 4 | -5.94139242 | 1.05822444 |
| 5 | -6.3869772 | 1.18472898 |
| 6 | -6.42374039 | 1.21052182 |
| 7 | -6.38610697 | 1.61750984 |
| 8 | -6.50890446 | 1.33429134 |
| 9 | -6.97040892 | 0.929638445 |
| 10 | -6.63670635 | 1.18752587 |
| 11 | -7.7294302 | 1.09942734 |
| 12 | -7.98590326 | 0.726437182 |
| 13 | -7.38594723 | 1.07457232 |
| Co-ligand | -6.82321644 | 0.798828483 |
| Standard control (resveratrol) | -7.12138753 | 0.824170619 |
